# miRepress: modelling gene expression regulation by microRNA with non-conventional binding sites

**DOI:** 10.1038/srep22334

**Published:** 2016-02-29

**Authors:** Suman Ghosal, Shekhar Saha, Shaoli Das, Rituparno Sen, Swagata Goswami, Siddhartha S. Jana, Jayprokas Chakrabarti

**Affiliations:** 1 Computational Biology Group, Indian Association for the Cultivation of Science, Kolkata, West Bengal, 700032, India.; 2 Department of Biological Chemistry, Indian Association for the Cultivation of Science, Kolkata, West Bengal, 700032, India; 3 Gyanxet, BF 286 Salt Lake, Kolkata, West Bengal, 700064, India

## Abstract

Some earlier studies have reported an alternative mode of microRNA-target interaction. We detected target regions within mRNA transcripts from AGO PAR-CLIP that did not contain any conventional microRNA seed pairing but only had non-conventional binding sites with microRNA 3′ end. Our study from 7 set of data that measured global protein fold change after microRNA transfection pointed towards the association of target protein fold change with 6-mer and 7-mer target sites involving microRNA 3′ end. We developed a model to predict the degree of microRNA target regulation in terms of protein fold changes from the number of different conventional and non-conventional target sites present in the target, and found significant correlation of its output with protein expression changes. We validated the effect of non-conventional interactions with target by modulating the abundance of microRNA in a human breast cancer cell line MCF-7. The validation was done using luciferase assay and immunoblot analysis for our predicted non-conventional microRNA-target pair WNT1 (3′ UTR) and miR-367-5p and immunoblot analysis for another predicted non-conventional microRNA-target pair MYH10 (coding region) and miR-181a-5p. Both experiments showed inhibition of targets by transfection of microRNA mimics that were predicted to have only non-conventional sites.

microRNAs (miRNA) have been in focus the past decade^1^,^2^,^3^,^4^. In eukaryotic genome, a large part of the protein coding transcripts are post-transcriptionally regulated by miRNA-directed translational repression or mRNA decay^5^. miRNAs are identified as key players in many diseases including cancers and many experimental and computational studies are directed towards finding association of more miRNAs with diseases^6^,^7^,^8^,^9^,^10^. The molecular mechanism underlying miRNA-mediated target repression and the role of miRNA-target base pairing interaction in determining the pattern of target regulation have always been much debated issue^11^. While most of the plant miRNAs are seen to regulate their targets by endonucleolytic cleavage resulting from a mostly perfect complementary base pairing^12^, animal miRNAs predominantly work by translationally repressing their targets by an imperfect base pairing interaction^13^,^14^. There exist examples, though, of near perfect complementary base pairing interactions^15^ and target mRNA degradation or repression (like in plants) in case of animal miRNAs^16^,^17^. Generally, the interaction of a few bases in the 5′ end of miRNA (base position 2–7 or 2–8), i.e. the so called seed region, with the 3′ UTR of the target mRNA is considered to be important for target recognition by miRNA, as this type of interaction was seen to dominate the experimentally identified miRNA-target pairs^18^,^19^. However, recent studies pointed towards other types of miRNA target sites including bulges in the seed position and complementary sites from miRNA 3′ end. Hannon and colleagues have shown the prevalence of noncanonical miRNA-target interactions with bulged sites and compensatory sites from miRNA 3′ end^20^,^21^. There are also evidences of interactions with target sites in parts of mRNAs other than the 3′ UTR^22^,^23^,^24^,^25^,^26^. There have been reports of mammalian miRNAs regulating targets in a plant miRNA-like manner with a near perfect complementarity with its target involving central pairing (target pairing with the 9^th^–12^th^ nt of miRNA), resulting in mRNA cleavage or translational repression^16^,^17^. There are also reports of miRNA 3′ ends interacting with target mRNAs 5′ UTRs^27^. Interestingly, this study pointed towards the possibility of a dual end pairing interaction of miRNA-target, with miRNA 5′ end pairing with mRNA 3′ UTR and miRNA 3′ end pairing with mRNA 5′ UTR, leading to a stronger target repression (reflected by protein fold changes upon miRNA transfection). Crosslinking, ligation, and sequencing of hybrids (CLASH) analysis identified noncanonical binding motifs in AGO1 bound miRNA-mRNA pairs, including non-seed binding involving miRNA 3′ end^26^. Owing to the capability of an miRNA to have multiple target sites on a single mRNA, it is thought that the target repression level increases with the number of target sites present in the 3′ UTR of the target mRNA. And not just the number of target sites, target repression level has been seen to correlate with also the type of the target sites; here the target site type being determined by the number of bases in the seed region of miRNA (6-mer < 7-merA1 < 7-merm8 < 8mer)^28^. However, these studies are limited to the conventional miRNA-mRNA interaction pattern of miRNA 5′ end interacting with mRNA 3′ UTR. Now with emerging evidences of new classes of miRNA target sites in the coding region and the 5′ UTRs of mRNA, and the indication of possible roles of these non-conventional target sites in determining target repression level, we set to identify the roles of all possible miRNA target site types considering both miRNA 5′ end and 3′ end interactions with all three target regions; in mRNA 5′ UTR, coding region and 3′ UTR, both conserved and non-conserved, on the translational repression of the target, measured by protein-fold change upon miRNA transfection^29^,^30^. Interestingly, we found significant correlation of protein-fold change with non-conventional sites like miRNA 3′ end interaction with mRNA 3′ UTR and coding region. Importantly, miRNA targets in the 5′ UTR and coding region of mRNAs have been reported in other works^26^ and the compensatory interaction of miRNA 3′ end with mRNA has been reported earlier^26^,^27^ but, we detected miRNA targets showing repression (as measured by protein fold change upon miRNA transfection) by only non-conventional miRNA 3′ end interactions with target’s 3′ UTR or coding region. We performed computational predictions of human miRNA targets exclusively on a dataset of AGO-interacting regions within human mRNA transcripts from HEK293 cells^23^ and found considerably large number of AGO interacting sites, mapping into the genomic loci of a large number of mRNAs, which contained only non-conventional miRNA binding motif. Further, we experimentally verified our prediction about the effect of non-conventional miRNA 3′ end binding sites on the target mRNA 3′ UTR. Luciferase assay of WNT1 3′ UTR co-transfected with miR-367-5p, which contained no conventional binding site with miRNA 5′end seed match, but had two predicted non-conventional sites with miRNA 3′end matching, confirmed our prediction for the significance of miRNA 3′ end interaction with target mRNA 3′ UTR. We found that hsa-miR-367-5p could reduce the luminescence signal in cells transfected with luciferase gene tagged with 3′-UTR of WNT1 compared with non-specific control and this inhibition of luminescence signal is perturbed when treated with inhibitor of hsa-miR-367-5p. Also, treatment with mutant hsa-miR-367-5p, where the predicted target region (miRNA base 14–19) was mutated, showed the same result as in case of the inhibitor. We further analyzed the changes in the endogenous protein level of WNT1 in MCF7 cells after transfection of has-miR-367-5p by performing immunoblot analysis. The result shows reduction in endogenous WNT1 protein level after miR-367-5p transfection, compared to non-specific control. Further, to validate the significance of the nonconventional miRNA binding sites in the coding region of target, we transfected MCF-7 with exogenous GFP tagged MYH10 coding region (CDS) and mimic of has-miR-181a-5p. The coding region of MYH10 contains one non-conventional non-seed target site but no conventional target for that miRNA. Interestingly, at 48 hours past transfection, GFP signal was reduced greatly in has-miR-181a-5p transfected compared to non-specific control. We also performed immunoblot analysis of MYH10 in presence of miR-181a-5p. The result again confirmed our hypothesis as the protein level of MYH10 was successfully reduced after miR-181a-5p transfection. Following this finding, we next tried to develop a model to predict the degree of miRNA target regulation in terms of protein fold changes from the number of different conventional and non-conventional target sites present in the target. In this model we included 9 types of target sites which showed good correlation (p < 0.05) with predicted target’s protein fold change out of total 54 types of sites considered in our study. Using these 9 interaction types as feature vector, we trained an artificial neural network with a set of miRNA-target pairs from the target sets of three miRNAs, miR-1, miR-124 and miR-181a^29^. We measured the correlation of the neural network regression output and protein fold changes on a separate set of miRNA-target pairs from the targets of 7 different miRNAs collected from two datasets^29^,^30^. Our model showed sufficiently good correlation with the target protein-fold changes. We believe this model is a good resource for evaluating relation of miRNA-target interaction patterns with the translational repression of the target in mammals.

## Results

### miRNAs can target transcripts with non-conventional sites as verified from PAR-CLIP dataset

**Figure 1:**
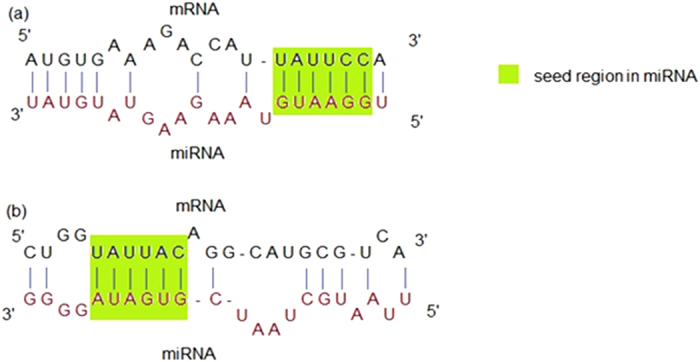
(**a**) miRNA 5′ end (position 2–7 for 6-mer seed) interaction with target mRNA (**b**) miRNA 3′ end (position 13–18 for 6-mer seed) interaction with target mRNA.

To determine if miRNAs can contain non-conventional, that is non seed-motif binding sites (as depicted in Fig. 1b compared to conventional binding in 1a) on cellular mRNA transcripts, we performed computational predictions of human miRNA targets exclusively on a dataset of AGO-interacting regions within human mRNA transcripts from HEK293 cells. The AGO PAR-CLIP dataset on HEK293 cells was collected from Hafner study^23^. From a set of 17319 non-overlapping AGO interacting sites within human transcriptome, 4877 sites were detected with only non-conventional miRNA binding sites. These non-conventional binding involved a pairing with the 13^th^–19^th^ nucleotide position of an miRNA instead of the conventional pairing with the 2^nd^–8^th^ nucleotide position. We mapped these sites onto the genomic coordinates of human mRNAs (collected from UCSC genome browser). 1247 non-conventional binding sites for 683 miRNAs were found on 630 different mRNA transcripts that do not contain any conventional seed sequence matching with any human miRNA (see Supplementary table S1). This result suggests that miRNAs can target transcripts not only by conventional miRNA 5′ end seed match, but also by non-conventional pairing with bases from miRNA 3′ end.

### Investigating the effects of conventional and non-conventional miRNA target sites on protein-fold change

To investigate the effects of different types of miRNA target sites on the translational repression of the target, we calculated the number of all possible types of target sites (6-mer, 7-merA1, 7-merm8 and 8-mer and 11-mer central pairing including base 9-12 of miRNA) considering both miRNA 5′ end and 3′ end interactions on all three regions of mRNA; 5′ UTR, open reading frame (ORF) and 3′ UTR. We considered conserved and non-conserved target sites separately. A total of 54 types of target sites (see Table 1) were calculated for each miRNA-mRNA pair from the protein-fold change datasets from Baek^29^. Table 1 gives a listing of all types of miRNA-target interaction patterns considered in our study. For all types of target sites, we calculated the correlation of the target’s protein fold change with the number of target sites for each type separately. For target sites harbouring central pairing like plant miRNAs, we did not find good correlation with the protein-fold changes in these datasets for human and mouse. This result may indicate possibly these types of central pairing interactions are not significantly related to the translational repression of the target in mammals.

### Correlation of the number of conserved and non-conserved conventional target sites (interaction with miRNA 5′ end) with protein expression fold changes

**Figure 2:**
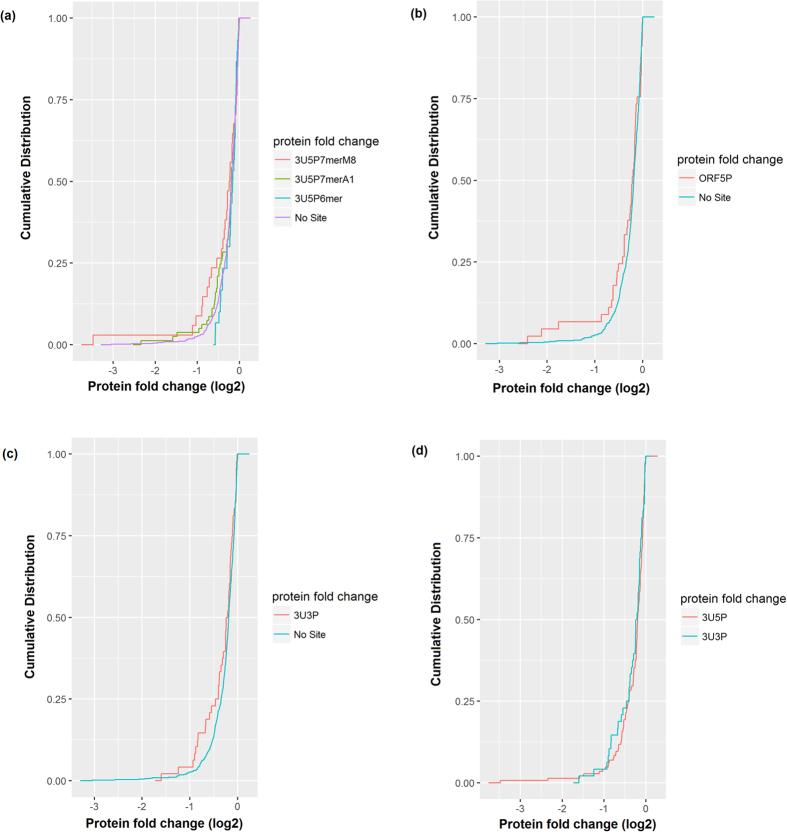
(**a**) The cumulative distribution plot of protein fold changes (log2) after miRNA transfection for targets with one miRNA 5′ end 6-mer (3U5P6mer, red line) or 7-merA1 (3U5P7merA1, green line) or 7-merm8 (3U5P7merM8, cyan line) site in the target’s 3′ UTR versus that of the targets with no miRNA site (purple line) in the target. Here 6-mer sites are exclusively those sites which contain a 6-mer seed match in miRNA position 2–7. Similarly, 7-merA1 sites contains the nucleotide ‘A’ in the target opposite of the 1^st^ nucleotide in the miRNA sequence followed by 6-mer seed match in miRNA position 2–7 and 7-merM8 sites contains 7-mer seed matches in miRNA position 2–8. The 6-mer sites do not include in the larger 7-merA1 or 7-merM8 sites. All number of sites for all types (6-mer, 7-merA1 and 7-merM8) combines the number of conserved and non-conserved sites in the respective category. Targets with only one 7-merA1 site and targets with only one 7-merm8 site showed more repression in protein level than that of the targets with no miRNA site. But the targets with only one 6-mer site did not show more repression in protein level than the targets with no site, signifying that only one 6-mer miRNA site did not significantly affect target repression in protein level. (**b**) The cumulative distribution plot of protein fold changes (log2) after miRNA transfection for targets with one miRNA 5′ end site in the target’s ORF (ORF5P, red line) versus that of the targets with no miRNA site (cyan line) in the target. (**c**) The cumulative distribution plot of protein fold changes (log2) after miRNA transfection for targets with one miRNA 3′ end site in the target’s 3′ UTR (3U3P, red line) versus that of the targets with no miRNA site (cyan line) in the target. (**d**) The cumulative distribution plot of protein fold changes (log2) after miRNA transfection for targets with one miRNA 5′ end site in the target’s 3′ UTR (3U5P, red line) versus that of the targets with one miRNA 3′ end site in the target’s 3′ UTR (3U3P, cyan line).

Our study identified high correlation with protein fold changes for all types of conventional miR 5′ end- mRNA 3′ UTR interactions (6-mer, 7-merA1, 7-merm8 and 8-mer). And it reflected the same increasing significance for 6-mer > 7-merA1 > 7-merm8 interactions. Figure 2a shows the cumulative distribution of protein fold changes (log2) of targets with only one miRNA 5′ end 6-mer or only one miRNA 5′ end 7-merA1 or only one miRNA 5′ end 7-merm8 site in their 3′ UTR versus that of the targets with no miRNA target sites. However, the target transcripts with only one miRNA 5′ end 6-mer site in the 3′ UTR, but no other sites, did not show more repression in the protein level than the transcripts with no-site. Which indicates only a single 6-mer miRNA site did not significantly affect target repression in protein level. But the situation changes when multiple numbers of 6-mer sites are involved. For conserved target sites, only the 6-mer seed region interaction showed correlation with protein fold changes, while for other seed motifs, conserved target sites lost significance for determining protein fold change levels.

Conventional target sites (miRNA 5′ end interactions) on mRNA coding region and 5′ UTR are also significantly related to protein fold changes. Apart from conventional target sites in mRNA 3′ UTRs, number of 6-mer target sites on mRNA coding region and in 5′ UTR (considering miRNA 5′ end interactions) showed good correlation with the target’s protein fold changes, when only non-conserved target sites were considered. Conserved target sites in these non-conventional target regions, on the other hand showed little significance in determining protein fold changes. Figure 2b shows the cumulative distribution of protein fold changes (log2) of targets with one miRNA 5′ end 6-mer site in their ORF versus that of the targets with no miRNA target sites.

### Non-conserved non-conventional target sites (miRNA 3′ interactions) on mRNA 3′ UTR and coding region are significantly correlated to protein fold changes

Following the studies of Lee^27^, we set to investigate the possibility of non-conventional miRNA-target interactions, which involve pairing of the target mRNA with miRNA 3′end, for all three regions in the target mRNA (5′ UTR, ORF and 3′ UTR). Interestingly, we found significant correlation of the number of non-conserved non-conventional target sites in the 3′ UTR and coding region with the protein fold changes of the target, especially for 6-mer and 7-merm8 target sites (p values < 10^−2^ for 6-mer and 7-merm8 sites in 3′ UTR and p value < 0.05 for 6-mer sites in ORF). Figure 2c shows the cumulative distribution of protein fold changes (log2) of targets with one miRNA 3′ end site in their 3′ UTR versus that of the targets with no miRNA binding site and Fig. 2d shows the cumulative distribution of protein fold changes (log2) of targets with one miRNA 5′ end site in their 3′ UTR versus that of the targets with one miRNA 3′ end site in their 3′ UTR. Notably, we detected several miRNA-targets from Baek and Selbach datasets (73 miRNA-target interactions) with only these non-conventional target sites that displayed significant changes in protein expression. Supplementary Table S2 lists some of the examples for this kind of target sites with the respective log 2 fold changes in protein expression. However, this type of non-conventional miRNA-target pattern was not widespread for conserved target sites (which is consistent with the study by Lee^27^), and these conserved target sites are non significant for determining protein fold change levels.

### ANN regression of a set of 9 most important miRNA-target interaction patterns to determine target repression level

From the set of 54 all possible miRNA-target interaction patterns, we isolated 9 miRNA-target interaction types based on the correlation of the number of sites with protein fold changes. Table 2 gives the list of these 9 interaction types with their respective correlation with protein-fold changes and P-values for the correlations. For these 9 interaction types, we regressed the number of target sites against the protein-fold changes for all the miRNA-target pairs. We used an artificial neural network (ANN) trained with a training set of 1501 miRNA-target pairs (Supplementary table S3a), randomly selected from the target sets of miR-1, miR-124 and miR-181a^29^. The ANN regression model was evaluated by 10-fold cross-validation where it achieved root mean squared error (RMSE) 0.29. We compared the ANN regression model with two other regression models; sequential minimal optimization (SMO) and MLPR regression models. Our ANN regression model showed lower RMSE on 10-fold cross validation (Supplementary table S3b). We measured the correlation of the neural network regression output and protein fold changes on 1508 miRNA-target pairs in human from the targets of 7 different miRNAs from two sets of data. The first set was an independent set (entirely non-overlapping with the training set) of 1376 miR-target pairs from the target sets of 3 human miRNAs, miR-1, miR-124 and miR-181a from Baek^29^ (Supplementary table S3c). The test set gave significant correlation of the target protein fold change with the neural network output (r = 0.1, p < 0.01). For further evaluation of the ANN regression model we shuffled the training and testing datasets 100 times, each time randomly drawing miRNA-target pairs from the Baek dataset and including in either the training set or the testing set but keeping the total number of pairs in training and testing set same as before (i.e. 1501 pairs in the training set and 1376 pairs in the testing set). Each time we trained and tested the ANN regression model separately, keeping the training parameters same and noted the average correlation coefficient of the ANN output with the protein fold change. The average correlation coefficient after 100 times shuffling was also statistically significant (r = 0.058, p < 0.05), thereby proving the performance of the ANN model. The second test set contained 153 miRNA-target pairs from the target sets of 4 miRNAs, let-7b, miR-16, miR-155 and miR-30 from Selbach^30^ (Supplementary table S3d). This dataset also gave significant correlation (P < 0.05 on Selbach dataset) of protein fold changes with the neural network regression output. To check the significance of nonconventional miRNA-target sites in mouse, we used miR-223 depleted dataset from Baek study^29^, where we calculated miR-223 binding sites for 768 mRNAs that were significantly upregulated after miR-223 knockdown (compared to mock). We used our ANN regression model to calculate the repression score from 9 types of conventional and nonconventional miRNA-target sites and found correlation of protein fold change with the output. Importantly, with our model it is possible to predict protein repression levels for targets lacking conventional miRNA seed region (miRNA 5′ end) interactions. As reported in the previous section, among the total test set, we found 113 miRNA-target interactions with only non-conventional sites (miRNA 3′ end interactions). For these special miRNA-target pairs we also found significant correlation of our algorithm score with their respective protein fold changes (p value < 0.05).

### Validation of non-conventional binding of hsa-miR-367-5p in the 3′-UTR of WNT1 by luciferase assay

**Figure 3:**
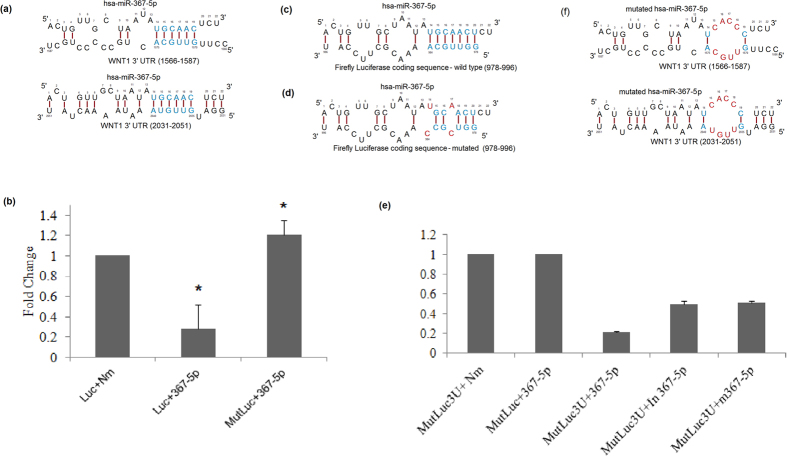
Inhibition of luciferase signal by hsa-367-5p. (**a**) Predicted interaction of hsa-miR-367-5p and WNT1 3′ UTR sequence. miR-367-5p has no conventional 5′ end seed matched site in WNT1 3′ UTR, but it contains two non-conventional 3′ end matched binding site in the positions 1566-1575 and 2035-2040 in the WNT1 mRNA 3′ UTR sequence. (**b**) MCF-7 cells were co-transfected with mimic of hsa-367-5p miRNA or non-specific control RNA oligo (Nm) and plasmid DNA encoding wild-type luciferase gene or mutant luciferase gene. Note that wild-type, not the mutant, luciferase gene was inhibited by hsa-367-3p miRNA. The luminescence signal in wild-type luciferase was reduced by approximately 71%. (**c**) Predicted interaction of hsa-miR-367-5p and luciferase coding sequence. miR-367-5p has no conventional 5′ end seed matched site in luciferase gene, but it contains one non-conventional 3′ end matched binding site in the 978–984 region in the luciferase gene coding sequence. (**d**) The predicted binding site of hsa-miR-367-5p on firefly luciferase coding sequence is mutated (GGUUGCA- > GGUCGC) so that hsa-miR-367-5p could no longer bind and repress luciferase gene. (**e**) MCF-7 cells were co-transfected with mimic hsa-miR-367-5p miRNA or inhibitor of hsa-miR-367-5p or mutant hsa-miR-367-5p or non-specific control RNA oligo and plasmid DNA encoding mutant luciferase gene tagged with 3′-UTR of WNT1. The luminescence signal was reduced approximately 79% in case of co-transfection of mimic has-miR-367-5p. Note that luciferase signal was reduced in the presence of hsa-367-5p miRNA and that ~30% luciferase was recovered in presence of has-miR-367-5p inhibitor or mutant hsa-miR-367-5p. *p < 0.05, Nm vs hsa-miR-367-5p. (**f**) hsa-miR-367-5p is mutated in its predicted binding site in its 3′ end i.e. base 14–19 (UGCAAC- > UCACCC) so that the binding of mutant hsa-miR-367-5p 3′ end with the two sites in WNT1 3′ UTR is perturbed.

Our computational algorithm suggested that, miRNA hsa-miR-367-5p has two non-conventional binding sites at the 3′-UTR of WNT1 gene in positions 1566–1575 and 2035–2040 in the WNT1 mRNA 3′ UTR sequence (Fig. 3a). Our algorithm predicted a repression score of 0.799 for miR-367-5p mediated repression of WNT1. To validate the role of the non-conventional binding sites, we used luciferase reporter gene assay system in MCF-7 cell line, in which mimicking form of miRNA hsa-367-5p was co-transfected with plasmid DNA encoding luciferase gene tagged with 3′-UTR of WNT1. Cells transfected with plasmid DNA encoding only luciferase gene and a non-specific control RNA oligo was considered as a positive control for luciferase signal.

In this assay system, to ensure no interference from luciferase gene alone, we first co-transfected mimic of miRNA hsa-367-5p with plasmid DNA encoding luciferase gene. We found 3.5 ± 0.23 fold reductions in the luminescence intensity in hsa-367-5p treated cells compared with negative oligo treated cells (Fig. 3b), suggesting the possibility of existence of binding site of hsa-367-5p in the coding region of luciferase. Our searching algorithm suggests the existence of a non-conventional binding site at 978–984 nt in the coding region of luciferase gene; interestingly no conventional site was found (Fig. 3c). Our algorithm indicated a predicted repression score of 0.61 for firefly luciferase gene mediated by miR-367-5p. We mutated two nucleotide positions (T981C and A984C) in the region where seed region of hsa-367-5p is predicted to bind (Fig. 3d), and co-transfected the mutated luciferase gene (T981C and A984C) with hsa-367-5p in MCF-7 cells. Importantly, hsa-367-5p was unable to reduce the expression of mutant luciferase gene (Fig. 3b), suggesting that a non-conventional site existed in the coding region of luciferase gene, and abolition of the binding site by introducing mutation interfered with binding to hsa-367-5p. So, we used mutant version of luciferase construct to validate the existence of non-conventional site in the 3′-UTR of WNT1. We found that hsa-367-5p could reduce 4.8 ± 0.01 fold luminescence signal in cells transfected with mutated luciferase gene tagged with 3′-UTR of WNT1 compared with mutated luciferase gene alone (Fig. 3e). These results suggest that luciferase gene tagged with 3′-UTR of WNT1 construct has non-conventional binding sites located in the 3′-UTR of WNT1 and also in the coding region of luciferase gene.

We validated the binding specificity of has-miR-367-5p in the 3′ UTR of WNT1 in two ways. First, we added inhibitor of has-miR-367-5p to MCF7 cells that were previously co-transfected with hsa-miR-367-5p and construct of WNT1 3′ UTR tagged with mutated luciferase (the mutated luciferase does not contain any binding site for has-miR-367-5p). Second, we added mutant has-miR-367-5p to the same MCF7 cells. The mutant hsa-miR-367-5p was made by replacing the 14^th^–19^th^ base positions i.e., where it was predicted to bind the target WNT1 noncanonically, from UGCAAC to UCACCC. Thus the binding of the mutant hsa-miR-367-5p with WNT1 3′ UTR by complementarity of the target with the miRNA base 14–19 is impaired (Fig. 3f). We found that the inhibitor of hsa-miR-367-5p and the mutant hsa-miR-367-5p perturb hsa-miR-367-5p mediated repression of WNT1 3′ UTR in similar way (Fig. 3e). While after adding the inhibitor of hsa-miR-367-5p the luminescence signal increased 2.3 fold compared to mimic hsa-miR-367-5p treated cells, adding the mutant hsa-miR-367-5p increased 2.5 fold luminescence signal. In both cases there were ~30% restoration of luminescence signal compared to mimic hsa-miR-367-5p treated cells. These results indicate that non-seed interaction through bases 14–19 plays a role in the binding of hsa-miR-367-5p to the target WNT1 3′ UTR.

### Validation of non-conventional binding of hsa-miR-367-5p in the 3′-UTR of WNT1 and has-miR-181a-5p in the coding region of MYH10 by immunoblot analysis

**Figure 4:**
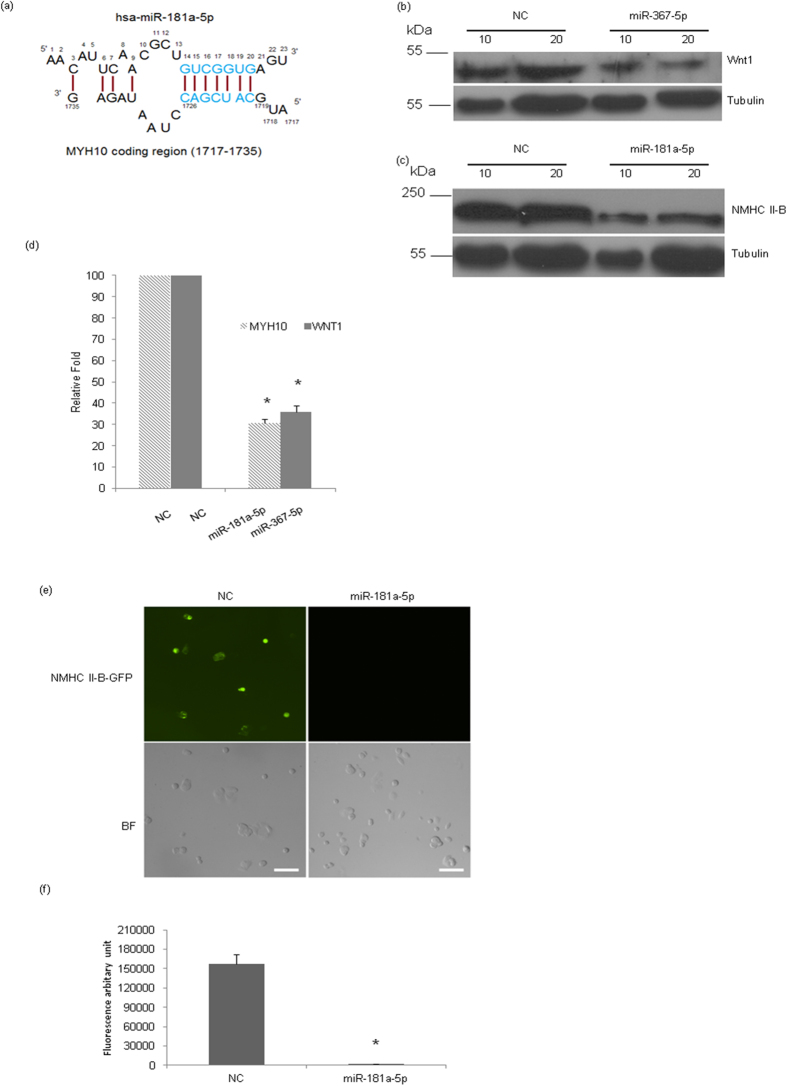
Inhibition of Wnt1 and NMHC II-B expression by miRNA. (**a**) Predicted interaction of hsa-miR-181a-5p and MYH10 coding sequence. miR-181a-5p has no conventional 5′ end seed matched site in MYH10, but it contains one non-conventional 3′ end matched binding site in the 1720–1726 region in the MYH10 gene coding sequence. (**b**) MCF-7 cells were transfected with mimic miR-367-5p, miR-181a-5p, or non-specific control (NC). After 72 hours of post transfection cell lysates were prepared and two different amounts of cell lysates were loaded for each sample and immunoblotted with the indicated antibodies. Tubulin was used as a loading control. (**d**) The band intensity was calculated by ImageJ software by considering the band intensity of non-specific control sample as “100”. (**e**) MCF-7 cells were co-transfected with GFP tagged NMHC II-B and non-specific control or hsa-miR-181a-5p. 48 hours post transfection, fluorescence images were captured. Top left panel, the green fluorescence signal is coming from GFP tagged NMHC II-B, transfected with non-specific control miRNA, right panel, the inhibition of fluorescence intensity transfected with has-miR-181a-5p mimic miRNA. Bottom panel is showing their corresponding bright field images. (**f**) Fluorescence intensity was calculated using ImageJ software and shown in (**f**). *p < 0.05, NC vs 181-5p, Scale bar ‘−’ 20 μm.

For further validation of the significance of miRNA 3′ end mediated interaction with the target 3′ UTR, we checked the endogenous protein level of WNT1 after transfection of has-miR-367-5p in MCF-7 cells, compared to non-specific control. At 72 hours after transfection of miR-367-5p, the expression level of WNT1 was reduced to 30% compared to non-specific control (Fig. 4b,d).

To validate our prediction on the significance of non-conventional miRNA binding sites in the coding region of the target, we used another pair of miRNA-target, hsa-181a-5p and MYH10 (Non-muscle myosin II B or NMHC II B). Our algorithm predicted a repression score of 0.791 for miR-181a-5p mediated repression of MYH10. MYH10 contains one non-conventional target site for hsa-miR-181a-5p in its coding region (position 1717–1735 on MYH10 mRNA, Fig. 4a), but no conventional target site in its coding region. At 72 hours post transfection, MCF7 cells transfected with hsa-miR-181a-5p mimic showed almost 70% inhibition (Fig. 4c,d). We also transfected ectopically GFP tagged MYH10 (NMHC II-B) coding region and mimic of has-miR-181a-5p in MCF-7 cells. GFP does not contain any target site for hsa-miR-181a-5p. Interestingly, at 48 hours past transfection (Fig. 4e,f), GFP signal was reduced to 10% in hsa-miR-181a-5p transfected cells compared to non-specific control. Taken together, these data suggest that miRNAs can interact with its target mRNA by non-conventional (other than seed-matched) binding sites and reduce the target’s protein expression level.

### Comparison of our model with the TargetScan context score method for prediction of target repression level

To compare our model’s efficiency in predicting the target repression level with the widely accepted context score model of TargetScan^28^, we measured our algorithm score for 233 miRNA-target pair (subset of our total dataset) for which we also collected context score (downloaded from TargetScan database). Our algorithm score was significantly correlated with the measured protein fold change as well as context score for this dataset (p < 0.05 in both cases). Moreover, our model is able to predict repression levels for targets with only miRNA 3′ end interactions that cannot be detected by TargetScan context score model. Supplementary table S2 lists some of these targets in our dataset that shows significant changes in protein level upon miRNA transfection, but without any conventional miRNA 5′ end interaction sites and hence could not be detected by Targetscan model. But our model successfully predicts the protein repression levels for these targets.

### miRepress; an in-silico tool for prediction of target repression level for a given miRNA-mRNA pair

Based on our model for predicting target repression level, we developed an in-silico tool “miRepress” that predicts target repression level in terms of protein fold change from the presence of different types of miRNA interaction sites. It takes as the target mRNA name, in terms of gene id or RefSeq accession of the mRNA and outputs the predicted repression level along with the list of predicted interactions. Presently miRepress works for human miRNA-mRNA pairs.

## Discussion

miRNA mediated target repression has been an issue, while the true nature of miRNA-target mRNA interaction in mammals is still somewhat elusive. The seed-interaction model is widely followed in miRNA target identification as there has been found an enrichment of this type of interaction in experimentally validated mammalian miRNA targets. But, clearly, that does not take account of all miRNA-target interactions, as there are evidences of other non-seed interactions in mammalian systems. For the first time we analyzed the impact of all possible miRNA target sites over all three regions of the target mRNA (summing up to 54 different types of miRNA-target interactions) on the target’s repression at the protein level. We considered both miRNA 5′ and 3′ end interactions with varying seed region length (6-mer, 7-merA1, 7-merm8 and 8-mer) and found significant correlation with measured protein-fold changes upon miRNA transfection for 3 types of miRNA 3′ end interactions, mainly in target mRNA’s 3′ UTR and coding region. Based on a validation on 7 different miRNA transfection data, we propose that these types of non-conventional interactions have contribution in determining target repression level. This conclusion of ours is consistent with a previous reports that pointed towards the role of non-canonical miRNA sites on target repression in human^20^,^25^,^27^. We identified novel miRNA-target interaction types comprising miRNA 3′ end pairing with the target mRNA’s 3′ UTR and coding region. To determine if miRNAs can contain non-conventional, that is sites other than seed-motif binding sites on cellular mRNA transcripts, we performed computational predictions of human miRNA targets exclusively on a dataset of AGO-interacting regions within human mRNA transcripts from HEK293 cells. The dataset was collected from the AGO PAR-CLIP experiments of Hafner^23^. We found considerably large number of AGO interacting sites which contained only non-conventional miRNA binding motif. These non-conventional binding involved a pairing with the 13^th^–19^th^ or 14^th^–19^th^ nucleotide position of an miRNA instead of the conventional pairing with the 2^nd^–8^th^ nucleotide position. When these sites were mapped into the genomic co-ordinates of human mRNA transcripts, 1247 non-conventional binding sites for 683 miRNAs were found on 630 different mRNA transcripts that did not contain any conventional seed sequence matching with any human miRNA. This result suggests that miRNAs can target transcripts not only by conventional miRNA 5′ end seed match, but also by non-conventional pairing with bases from miRNA 3′ end.

Further, we experimentally verified our prediction about the effect of non-conventional miRNA 3′ end binding sites on the target mRNA 3′ UTR. Our computational algorithm suggested that, miRNA hsa-miR-367-5p has two non-conventional binding sites at 3′-UTR of WNT1 gene (Fig. 3a), and predicted repression score for WNT1 by miR-367-5p was 0.799. To validate the existence of a non-conventional binding site, we used luciferase reporter gene assay system in MCF-7 cell line, in which miRNA hsa-miR-367-5p mimic was co-transfected with plasmid DNA encoding luciferase gene tagged with 3′-UTR of WNT1. Remarkably, we found that the coding sequence of firefly luciferase contains a non-conventional 7-merm8 binding site with the 3′ end of miR-367-5p (Fig. 3c) and our algorithm predicted a repression score of 0.61 for luciferase sequence mediated by miR-367-5p. We found out that co-transfection of wild-type luciferase with miR-367-5p results in approximately 3.5 fold reduction in luminescence signal compared to the nonspecific control. The effect of this non-conventional binding site was removed when we used a version of luciferase that was mutated in the region where seed region of hsa-367-5p is predicted to bind (Fig. 3d,b). But this observation suggested the repressive role of miRNA 3′ end interaction with target mRNA coding sequence, which was again in sync with our prediction. However, when we used mutant version of luciferase construct to validate the existence of non-conventional site in the 3′-UTR of WNT1, we found that hsa-367-5p could reduce approximately 4.8 fold luminescence signal in cells transfected with luciferase gene tagged with 3′-UTR of WNT1 compared with non-specific control (Fig. 3e). This observation from the experiment again was in sync with the algorithm prediction as WNT1 showed more repression by miR-367-5p compared to the wild-type luciferase (4.8 fold in WNT1 compared to 3.5 fold in wild-type luciferase, Fig. 3b,e) as predicted by the algorithm score (0.79 in WNT1 compared to 0.61 in wild-type luciferase). When treated with inhibitor of miR-367-5p or mutant miR-367-5p, the luciferase luminescence signal restored by ~30% after 48 hours of transfection (Fig. 3e). This showed the effect of miRNA 3′ end interaction with target mRNA 3′ UTR. However, it may be possible that there is some residual effect of the discrete bulged base pairings from the 5′ end of the mutant miR-367-5p. As seen from Fig. 3f, the 2^nd^ target site on WNT1 3′ UTR (position 2031–2052) of miR-367-5p has a bulged pairing in its base position 1–6 with two mismatches and another bulged pairing in its base position 4–10 with two mismatches (Fig. 3f). Hannon and colleagues previously reported about functional G-bulged sites (at miRNA position 5-6) in the targets of miR-124 in mouse brain^20^. Also Helwak and colleagues have observed from AGO1-CLASH data, a pattern of miRNA target site that involves a less stable distributed base pairing^25^. Considering these observations we surmise that the bulged pairings (position 1–6 and 4–10) in one of the hsa-miR-367-5p target sites in WNT1 3′ UTR may have some effect and that may explain why the luciferase luminescence signal was not fully restored but restored by ~30% in the mutant miR-367-5p transfected case. Further examinations are needed in this direction and we may have to consider the effects of these new types of sites containing mismatched pairing in the miRNA base position 2–10.

For further proof of the effect of non-conventional miRNA binding on target protein expression, we performed immunoblot analysis on endogenous WNT1 and MYH10 in presence of miR-367-5p and miR-181a-5p respectively. As stated earlier, miR-367-5p has two non-conventional target site on WNT1 3′ UTR. MYH10 contains one non-conventional target site for miR-181a-5p in its coding region, but it does not contain any conventional seed-matched binding site for that miRNA in its coding region. In both cases, at 72 hours past transfection of mimics of miR-367-5p and miR-181a-5p respectively, MCF7 cells showed almost 70% inhibition compared to non-specific control. The repression score for the target-miRNA pairs WNT1-miR-367-5p and MYH10-miR-181a-5p, as predicted by our algorithm, was 0.799 and 0.791 respectively. In both cases the algorithm predicted repression and the experimentally verified repression were more or less similar, though in the experiment, MYH10 showed slightly more repression than WNT1 as opposed to the algorithm prediction (Fig. 4d). We may have to look further for the effects of other mismatched base-pairing interactions, as stated in the previous paragraph, for a more accurate prediction of target repression. Also, we checked that miR-181a-5p can reduce the protein level of exogenous MYH10. We transfected MCF-7 with exogenous MYH10 coding sequence (CDS) tagged GFP and mimic of hsa-miR-181a-5p. Interestingly, at 48 hours past transfection, GFP signal was reduced by 80% in has-miR-181a-5p transfected compared to non-specific control. These results indicate that miRNAs can interact with target mRNA by non-conventional binding sites and repress the target protein level.

Based on our findings, we developed a new model to predict target repression at the protein level that uses 9 different miRNA-target interaction sites including 3 non-conventional sites mentioned earlier. We regressed the number of these 9 different miRNA target sites against the protein-fold change using an artificial neural network. Our model was validated with two datasets of protein fold change upon miRNA transfection using 7 different miRNAs and our algorithm score was significantly correlated with the target protein repression level. Also, our algorithm score, when tested on a common dataset, is found to be significantly correlated with the widely used context score model^28^ that uses conventional target sites for prediction of target repression. In future our model can be improved by considering other important features around miRNA target sites (as considered in the context score model) for presently it uses only the information about number of different types of target sites to fairly predict the target repression level. Also, the target repression level is not solely dependent upon the single miRNA-target interactions. There are likely to be other determinants like target sites for co-expressed miRNAs or the target mRNA level, or more complex factors like presence of competing endogenous RNAs. We are considering these factors in the next version of our model. Our study can also be beneficial for prediction of off-targets of synthetic siRNAs used in cell culture or *in-vivo*. Exogenous siRNAs are known to target unintended transcripts in a miRNA-like manner, which results in unwanted toxicity^31^. Prediction and removal of these off-target effects are a major concern in synthetic siRNA designing and many studies, including our previous study, are directed towards this issue^32^,^33^. We speculate that consideration for non-conventional targets may be beneficial for identifying previously unidentified off-targets of synthetic siRNAs. For ease of use we have developed a tool, miRepress that predicts target repression level for a single miRNA and single target and made it available online (http://gyanxet-beta.com/mirepress.jsp). We believe our model is a valuable resource for researchers studying the impact on miRNAs on target at protein level in the light of new miRNA target site types.

## Methods

### AGO PAR-CLIP dataset from HEK293 cells

We collected AGO PAR-CLIP dataset from Hafner *et al*.^23^. The dataset contained FASTA sequences of mRNA fragments bound by miRNPs from HEK293 cell lines stably expressing FLAG/HA-tagged AGO or TNRC6 family proteins. 17,319 sequence clusters, each of length 41 nucleotides and centred over the predominant cross-linking sites were listed in the dataset. It included sequence reads from all AGO PAR-CLIP experiments (AGO 1–4).

### Protein-fold change data upon miRNA transfection

We used the protein-fold change data from the study of Baek, where they measured protein-fold changes upon transfection of three different miRNAs separately; miR-1, miR-124 and miR-181a on HeLa cells^29^. We used records with a negative value of log 2 fold change, and records with Refseq mRNA accessions only; which resulted in 1172 records from miR-1 transfected set, 793 records from miR-124 transfected and 913 records from miR-181a transfected set.

Another data source for protein fold change upon miRNA transfection was used from pSILAC project; from where we collected protein fold changes upon transfection of miR-155, miR-16, miR-30 and let-7b into HeLa cells^30^. The total set was filtered similarly as the Baek dataset. Finally we had 200 records from miR-155 transfected, 98 from miR-16 transfected, 101 from miR-30 transfected and 100 from let-7b transfected sets.

### Prediction of all possible miRNA-target sites

For prediction of the number of all possible miRNA-target sites on the target mRNA, we considered the three regions (5′ UTR, CDS and 3′ UTR) separately. For each region we used a 25 base window on the target to run a modified version of the SmithWaterman alignment (as used in the miRanda algorithm) to find complementary alignment of the 25 base target region with the miRNA. For prediction of different types of miRNA target sites (6-mer, 7-mer, 7-merA1 and 8-mer), we changed the seed region definition in each case^34^. For prediction of miRNA 3′ end interactions, we considered the base position 14–19 from miRNA 5′ end instead of position 2–7 used for predicting miRNA 5′ end interactions (for 6-mer seed type), and predicted interaction in similar way as described above.

### Consideration for target site conservation

We treated conserved and non-conserved target sites separately. We searched for target region conserved among human, chimp, mouse, rat and dog. Genome wide conservation data generated using multiz 46-way alignment (for 46 vertebrate species)^35^ was downloaded from UCSC genome browser^36^. Genomic regions (within human genome) conserved within human, chimp, mouse, rat and dog are then extracted and mapped within the coordinates of human mRNAs (downloaded from UCSC genome browser) to get the location of the conserved regions within human mRNAs. Conserved regions of length 8 bases or more are only considered.

### Artificial neural network (ANN) regression model to predict protein-fold changes with the number of different target sites

From a total of 54 target site types, we identified 9 different target site types (from both conventional and non-conventional sites), that showed significant correlation with the protein fold changes. An artificial neural network was trained with the numbers of these 9 different target sites for 1501 miRNA-mRNA target pairs from Baek dataset. The architecture of the neural network was 9 × 4 × 1, i.e. 9 input nodes, 4 nodes in the hidden layer and one output node; with learning rate 0.01. We downloaded the nen package for neural network implementation in Java to perform the regression (http://aarnet.dl.sourceforge.net/project/nen/nen.jar). We compared performance of ANN regression with SMO regression and MLPR regression in WEKA tool for testing machine learning algorithms^37^.

### Cell Culture and Transfection

MCF-7 cells (ATCC, Manassas, USA) were grown in Dulbecco’s modified Eagle’s medium (DMEM) supplemented with 10% FBS, 100 ug/ml Penicillin/Streptomycin and human insulin. Cells were maintained at 37 °C in a humidified chamber in presence of 5% CO2. 33 pmole of has-miR-367-5p mimic miRNA (purchased from Sigma Aldrich) or 100 nmole of inhibitor of hsa-miR-367-5p (purchased from Life Technologies, catalogue no. 4427975) or 100 nmole of mutant hsa-miR-367-5p (purchased from Life Technologies) or non-specific control RNA oligo (purchased from Sigma Aldrich, catalogue no. HMC0003) was co-transfected with a mixture of 500 ng of Firefly luciferase tagged with WNT1 3′UTR and 100 ng of Renilla luciferase plasmid DNAs or 33 pmole has-miR-181a-5p in 2 × 10^5^ MCF-7 cells using Lipofectamine^TM^2000 (Invitrogen, Carlsbad, USA) transfection reagent). At 48 h post transfection, cell lysates were prepared for detecting luciferase signal using dual luciferase assay kit (Promega, Madison, USA) in a luminometer (Thermo Fisher, Waltham, USA). Renilla luciferase was used as an internal control to normalize the Firefly luciferase signal. Fold change by hsa-367-5p miRNA was calculated by considering luminescence value from non-specific control RNA oligo treated cell lysate as “1”. Results were expressed as the mean ± SEM. One way ANOVA analysis followed by Bonferroni test was carried to determine the statistical significance. The differences were considered to be significant if *p* < 0.05.

### Point Mutation

Firefly luciferase gene vector and luciferase with 3′-UTR of WNT1 vector were a kind gift from Dr. Jong In Yook (Seoul, Korea). Mutation at position T981 and A984 was carried out using site directed mutagenesis. Mutation to C (Cytosine) at both places did not alter amino acid sequence. Mutation was verified with DNA sequencing.

### Electrophoresis and Immunoblotting

Cells were directly lysed with 2X Laemmli buffer containing 10% β-mercaptoethanol and boiled for 5 min. Two different amounts of samples were loaded for each sample and proteins were separated on 8% resolving and 4% stacking Tris-Glycine SDS-PAGE, and transferred to PVDF membrane, and blocked with 5% non-fat skim milk at room temperature for 1 hour. The membranes were incubated with rabbit polyclonal NMHC II-B (1:2000, Sigma), rabbit polyclonal WNT1 (1:1000, Santa Cruz) or mouse polyclonal GAPDH (1:4000, Santa Cruz) at 4 °C for overnight. The blot was washed with phosphate buffer saline (PBS) containing 0.05% Tween-20 (Sigma) and incubated with horse radish peroxidase-conjugated secondary antibodies against rabbit or mouse at room temperature for 2 hours and developed with SuperSignal^TM^ West Femto luminal enhancer solution (Thermo Scientific). The Luminescence signal was captured on Biomax MR Film (Eastman, Kodak, USA).

## Additional Information

**How to cite this article**: Ghosal, S. *et al*. miRepress: modelling gene expression regulation by microRNA with non-conventional binding sites. *Sci. Rep.* **6**, 22334; doi: 10.1038/srep22334 (2016).

## Supplementary Material

Supplementary Information

## Figures and Tables

**Table 1:** All 54 types of miRNA-target sites considered in our study.

Serial no.	Target region within mRNA	miRNA seed region	Seed-type
1.	3′ UTR	miRNA 5′ end	6-mer conserved
2.			6-mer non-conserved
3.			7-merA1 conserved
4.			7-mer A1 nonconserved
5.			7-merm8 conserved
6.			7-merm8 non-conserved
7.			8-mer conserved
8.			8-mer non-conserved
9.		miRNA 3′ end	6-mer conserved
10.			6-mer non-conserved
11.			7-merA1 conserved
12.			7-mer A1 nonconserved
13.			7-merm8 conserved
14.			7-merm8 non-conserved
15.			8-mer conserved
16.			8-mer non-conserved
17.	Open Reading Frame	miRNA 5′ end	6-mer conserved
18.			6-mer non-conserved
19.			7-merA1 conserved
20.			7-mer A1 nonconserved
21.			7-merm8 conserved
22.			7-merm8 non-conserved
23.			8-mer conserved
24.			8-mer non-conserved
25.		miRNA 3′ end	6-mer conserved
26.			6-mer non-conserved
27.			7-merA1 conserved
28.			7-mer A1 nonconserved
29.			7-merm8 conserved
30.			7-merm8 non-conserved
31.			8-mer conserved
32.			8-mer non-conserved
33.	5′ UTR	miRNA 5′ end	6-mer conserved
34.			6-mer non-conserved
35.			7-merA1 conserved
36.			7-mer A1 nonconserved
37.			7-merm8 conserved
38.			7-merm8 non-conserved
39.			8-mer conserved
40.			8-mer non-conserved
41.		miRNA 3′ end	6-mer conserved
42.			6-mer non-conserved
43.			7-merA1 conserved
44.			7-mer A1 nonconserved
45.			7-merm8 conserved
46.			7-merm8 non-conserved
47.			8-mer conserved
48.			8-mer non-conserved
49.	3′ UTR	miRNA central region (covering base position 9–12)	11-mer conserved
50.			11-mer non-conserved
51.	Open Reading Frame		11-mer conserved
52.			11-mer non-conserved
53.	5′ UTR		11-mer conserved
54.			11-mer non-conserved

**Table 2:** 9 miRNA-target interaction types chosen for final regression.

miRNA-target interaction type	Correlation coefficients of the number of target sites with target protein fold change	Significance (p-value) of number of sites in determining protein fold change
1. miRNA 5′ end- mRNA 3′ UTR 6-mer (3U5P6-mer) conserved	0.05	P < 0.05
2. miRNA 5′ end- mRNA 3′ UTR 6-mer (3U5p6-mer) non-conserved	0.093	P < 10^−4^
3. miRNA 5′ end- mRNA 3′ UTR 7-merA1 (3U5P7-merA1) non-conserved	0.125	P < 10^−6^
4. miRNA 5′ end- mRNA 3′ UTR 7-merm8 (3U5P7-merM8) non-conserved	0.14	P < 10^−6^
5. miRNA 3′ end- mRNA 3′ UTR 6-mer (3U3P6-mer) non-conserved	0.09	P < 10^−4^
6. miRNA 3′ end- mRNA 3′ UTR 7-merm8 (3U3P7-merM8) non-conserved	0.068	P < 10^−2^
7. miRNA 5′ end- mRNA ORF 6-mer (ORF5P6-mer) non-conserved	0.096	P < 10^−4^
8. miRNA 3′ end- mRNA ORF 6-mer (ORF3P6-mer) non-conserved	0.049	P < 0.05
9. miRNA 5′ end- mRNA 5′ UTR 6-mer (5U5P6-mer) non-conserved	0.091	P < 10^−4^
