# Supplementary material for: miRepress: modelling gene expression regulation by microRNA with non-conventional binding sites
Source: Sci Rep. 2016 Feb 29;6:22334. doi: 10.1038/srep22334 (PMC4770313; doi:10.1038/srep22334)
Supplement: Supplementary Information [file srep22334-s1.pdf]

Ghosal S., Saha S., Das S., Sen R., Goswami S., Jana S.S., Chakrabarti J. miRepress: modelling gene expression regulation by microRNA with non-conventional binding sites

Supplementary table S1. human miRNA targets identified from AGO PAR-CLIP dataset from HEK293 cells (Hafner et al, 2010), that contains no conventional seed matched binding sites, but only nonconventional binding sites involving miRNA 3' end.

mRNA RefSeq acc	miRNA name	sites_6-mer-3p conserved	sites_6-mer-3p not conserved	sites_7-merA1-3p conserved	sites_7-merA1-3p not conserved	sites_7-merM8-3p conserved	sites_7-merM8-3p not conserved	sites_8-mer-3p conserved	sites_8-mer-3p not conserved
NM_003737	hsa-miR-6716-5p	0	0	0	1	0	0	0	0
NM_003486	hsa-miR-539-5p	0	0	0	0	0	1	0	0
NM_017786	hsa-miR-590-5p	0	0	0	0	0	0	0	1
NR_033436	hsa-miR-218-1-3p	0	0	0	1	0	0	0	0
NM_001100912	hsa-miR-3166	0	0	0	0	0	0	0	1
NM_014256	hsa-miR-4528	0	0	0	0	0	1	0	0
NM_014757	hsa-miR-3116	0	0	0	0	0	0	0	1
NM_014757	hsa-miR-4784	0	0	0	1	0	0	0	0
NM_001005291	hsa-miR-4639-5p	0	0	0	0	0	0	0	1
NM_005605	hsa-miR-383	0	0	0	0	0	1	0	0
NM_005605	hsa-miR-4701-	0	1	0	0	0	0	0	0

	3p								
NM_001080411	hsa-miR-3680-3p	0	0	0	0	0	1	0	0
NM_001080411	hsa-miR-4671-3p	0	0	0	1	0	0	0	0
NM_002501	hsa-miR-939-3p	0	0	0	0	0	0	0	1
NM_024941	hsa-miR-1298	0	0	0	0	0	0	0	1
NM_017984	hsa-miR-4254	0	0	0	0	0	0	0	1
NM_174921	hsa-miR-5187-5p	0	0	0	1	0	0	0	0
NM_032270	hsa-miR-4459	0	0	0	0	0	1	0	0
NM_032270	hsa-miR-4793-5p	0	0	0	1	0	0	0	0
NM_032270	hsa-miR-5003-5p	0	0	0	0	0	0	0	1
NM_001099784	hsa-miR-5002-3p	0	1	0	0	0	0	0	0
NM_001099784	hsa-miR-6506-3p	0	0	0	0	0	1	0	0
NM_021961	hsa-miR-4471	0	0	0	0	0	1	0	0
NM_004521	hsa-miR-3655	0	1	0	0	0	0	0	0
NM_004521	hsa-miR-4729	0	0	0	0	0	0	0	1
NM_00452	hsa-	0	0	0	0	0	0	0	1

1	miR-4778-3p								
NM_018268	hsa-miR-4725-5p	0	0	0	0	0	0	0	1
NM_007170	hsa-miR-5589-5p	0	0	0	1	0	0	0	0
NM_014188	hsa-miR-15a-5p	0	0	0	1	0	0	0	0
NM_014188	hsa-miR-670	0	0	0	0	0	0	0	1
NM_014188	hsa-miR-5580-5p	0	0	0	0	0	0	0	1
NM_005385	hsa-miR-302b-5p	0	0	0	0	0	0	0	1
NM_005385	hsa-miR-4639-3p	0	0	0	1	0	0	0	0
NM_003567	hsa-miR-9-5p	0	0	0	0	0	0	0	1
NM_003567	hsa-miR-587	0	1	0	0	0	0	0	0
NM_003567	hsa-miR-1231	0	0	0	1	0	0	0	0
NM_002039	hsa-miR-4666a-3p	0	0	0	0	0	0	0	1
NR_039997	hsa-miR-3912	0	0	0	0	0	1	0	0
NR_039997	hsa-miR-3150b-3p	0	0	0	1	0	0	0	0
NR_039997	hsa-miR-	0	0	0	0	0	0	0	1

	4471								
NR_037719	hsa-miR-148b-5p	0	0	0	0	0	0	0	1
NM_198581	hsa-miR-199a-3p	0	0	0	0	0	0	0	1
NM_198581	hsa-miR-199b-3p	0	0	0	0	0	0	0	1
NM_198581	hsa-miR-324-5p	0	0	0	0	0	0	0	1
NM_198581	hsa-miR-6075	0	0	0	0	0	0	0	1
NM_004560	hsa-miR-497-5p	0	1	0	0	0	0	0	0
NM_004560	hsa-miR-548d-5p	0	0	0	1	0	0	0	0
NM_004560	hsa-miR-4668-3p	0	0	0	0	0	1	0	0
NM_004560	hsa-miR-548ay-5p	0	0	0	1	0	0	0	0
NM_001024628	hsa-miR-122-5p	0	0	0	0	0	0	0	1
NM_015062	hsa-miR-3184-5p	0	0	0	0	0	0	0	1
NM_018685	hsa-miR-198	0	0	0	0	0	0	0	1
NM_001199292	hsa-miR-15a-3p	0	0	0	0	0	0	0	1
NM_001199292	hsa-miR-16-1-3p	0	0	0	0	0	0	0	1
NM_00119	hsa-	0	0	0	0	0	0	0	1

9292	miR-195-3p								
NM_080833	hsa-miR-10a-5p	0	0	0	0	0	0	0	1
NM_080833	hsa-miR-10b-5p	0	0	0	0	0	0	0	1
NM_080833	hsa-miR-4798-5p	0	0	0	0	0	1	0	0
NM_001483	hsa-miR-642a-5p	0	0	0	1	0	0	0	0
NM_001483	hsa-miR-3613-5p	0	0	0	1	0	0	0	0
NM_003603	hsa-miR-365a-5p	0	0	0	1	0	0	0	0
NM_0010177969	hsa-miR-5089-5p	0	0	0	1	0	0	0	0
NM_203315	hsa-miR-4761-3p	0	1	0	0	0	0	0	0
NM_001253693	hsa-miR-450b-3p	0	0	0	0	0	1	0	0
NM_001539	hsa-miR-16-5p	0	0	0	0	0	0	0	1
NM_152881	hsa-miR-33a-5p	0	0	0	1	0	0	0	0
NM_003592	hsa-miR-4684-3p	0	0	0	0	0	0	0	1
NM_153443	hsa-miR-5580-5p	0	0	0	0	0	1	0	0
NM_01459	hsa-	0	0	0	0	0	0	0	1

1	miR-1200								
NM_012255	hsa-miR-3619-5p	0	0	0	0	0	0	0	1
NM_001193582	hsa-let-7g-5p	0	0	0	1	0	0	0	0
NM_031468	hsa-miR-3657	0	0	0	1	0	0	0	0
NM_031468	hsa-miR-4766-5p	0	0	0	1	0	0	0	0
NM_000401	hsa-miR-5699	0	0	0	1	0	0	0	0
NM_014109	hsa-miR-1293	0	0	0	0	0	0	0	1
NM_014109	hsa-miR-4748	0	0	0	0	0	0	0	1
NM_015156	hsa-miR-554	0	0	0	0	0	0	0	1
NM_015156	hsa-miR-4638-3p	0	0	0	0	0	0	0	1
NM_015074	hsa-miR-100-3p	0	1	0	0	0	0	0	0
NM_015074	hsa-miR-362-5p	0	0	0	0	0	1	0	0
NM_015074	hsa-miR-1228-5p	0	0	0	1	0	0	0	0
NM_002222	hsa-miR-5008-5p	0	0	0	0	0	0	0	1
NM_173563	hsa-miR-144-5p	0	0	0	1	0	0	0	0
NM_173563	hsa-miR-145-3p	0	0	0	1	0	0	0	0

NM_173563	hsa-miR-3922-5p	0	0	0	1	0	0	0	0
NM_173563	hsa-miR-4725-5p	0	0	0	1	0	0	0	0
NM_001184896	hsa-miR-4632-5p	0	0	0	0	0	0	0	1
NM_014311	hsa-miR-1304-5p	0	0	0	1	0	0	0	0
NM_015373	hsa-miR-1301	0	0	0	1	0	0	0	0
NM_007170	hsa-miR-182-3p	0	0	0	1	0	0	0	0
NM_015466	hsa-miR-3621	0	0	0	0	0	0	0	1
NM_015466	hsa-miR-3909	0	0	0	0	0	1	0	0
NM_001043352	hsa-miR-617	0	0	0	0	0	0	0	1
NM_032448	hsa-miR-3130-5p	0	0	0	1	0	0	0	0
NM_001253726	hsa-miR-617	0	0	0	0	0	1	0	0
NM_001253726	hsa-miR-765	0	0	0	0	0	0	0	1
NM_001253726	hsa-miR-1193	0	0	0	0	0	0	0	1
NM_000646	hsa-miR-3614-3p	0	0	0	0	0	0	0	1
NM_020719	hsa-miR-1255b-	0	0	0	0	0	1	0	0

	5p								
NM_001134364	hsa-miR-199a-3p	0	0	0	0	0	0	0	1
NM_001134364	hsa-miR-199b-3p	0	0	0	0	0	0	0	1
NM_170692	hsa-miR-1323	0	1	0	0	0	0	0	0
NM_170692	hsa-miR-3660	0	0	0	1	0	0	0	0
NM_003432	hsa-miR-125b-2-3p	0	0	0	0	0	0	0	1
NM_003432	hsa-miR-4433-5p	0	0	0	1	0	0	0	0
NM_001190462	hsa-miR-3658	0	0	0	0	0	1	0	0
NM_001190462	hsa-miR-4669	0	0	0	0	0	0	0	1
NM_001100411	hsa-miR-3140-5p	0	1	0	0	0	0	0	0
NM_006773	hsa-miR-33b-3p	0	0	0	1	0	0	0	0
NM_006773	hsa-miR-1238-5p	0	0	0	1	0	0	0	0
NM_006773	hsa-miR-1203	0	1	0	0	0	0	0	0
NM_006773	hsa-miR-1912	0	1	0	0	0	0	0	0
NM_006773	hsa-miR-3130-5p	0	0	0	1	0	0	0	0
NM_00677	hsa-	0	0	0	0	0	1	0	0

3	miR-3157-5p								
NM_153603	hsa-miR-3167	0	1	0	0	0	0	0	0
NM_153603	hsa-miR-6512-3p	0	0	0	0	0	0	0	1
NM_181718	hsa-miR-548ar-3p	0	0	0	0	0	0	0	1
NM_001170536	hsa-miR-3119	0	1	0	0	0	0	0	0
NM_002335	hsa-miR-187-3p	0	0	0	1	0	0	0	0
NM_002335	hsa-miR-33b-3p	0	0	0	0	0	1	0	0
NM_002335	hsa-miR-1203	0	0	0	1	0	0	0	0
NM_002335	hsa-miR-3157-5p	0	0	0	1	0	0	0	0
NM_002335	hsa-miR-3175	0	0	0	0	0	0	0	1
NM_001204193	hsa-miR-495-5p	0	0	0	0	0	0	0	1
NM_001204193	hsa-miR-6718-5p	0	0	0	1	0	0	0	0
NM_001135032	hsa-miR-4433-5p	0	0	0	1	0	0	0	0
NM_022762	hsa-miR-219-5p	0	0	0	1	0	0	0	0
NM_022762	hsa-miR-6075	0	0	0	1	0	0	0	0
NM_00117	hsa-	0	0	0	0	0	1	0	0

0765	miR-1178-3p								
NM_020245	hsa-miR-892a	0	0	0	1	0	0	0	0
NM_001136225	hsa-miR-190a	0	0	0	0	0	0	0	1
NM_003567	hsa-miR-6073	0	0	0	0	0	0	0	1
NM_003622	hsa-miR-212-5p	0	0	0	0	0	1	0	0
NM_001142279	hsa-miR-3614-5p	0	0	0	0	0	1	0	0
NM_001142279	hsa-miR-3689f	0	0	0	0	0	0	0	1
NM_001102597	hsa-miR-486-3p	0	0	0	0	0	0	0	1
NM_001102597	hsa-miR-532-5p	0	1	0	0	0	0	0	0
NM_001102597	hsa-miR-3661	0	0	0	0	0	0	0	1
NM_001195308	hsa-miR-1908	0	0	0	1	0	0	0	0
NM_001195308	hsa-miR-3674	0	0	0	0	0	0	0	1
NM_002501	hsa-miR-199a-3p	0	0	0	0	0	1	0	0
NM_002501	hsa-miR-199b-3p	0	0	0	0	0	1	0	0
NM_002501	hsa-miR-432-5p	0	0	0	0	0	0	0	1
NM_002501	hsa-miR-1204	0	1	0	0	0	0	0	0

NM_002501	hsa-miR-4733-3p	0	1	0	0	0	0	0	0
NM_032194	hsa-miR-3200-3p	0	0	0	1	0	0	0	0
NM_032194	hsa-miR-4692	0	0	0	1	0	0	0	0
NM_032194	hsa-miR-5001-3p	0	0	0	1	0	0	0	0
NM_182758	hsa-miR-4655-3p	0	1	0	0	0	0	0	0
NR_003587	hsa-miR-1972	0	0	0	0	0	0	0	1
NM_021622	hsa-miR-3186-3p	0	0	0	1	0	0	0	0
NM_021622	hsa-miR-4793-3p	0	0	0	0	0	0	0	1
NM_016480	hsa-miR-1203	0	0	0	1	0	0	0	0
NM_001024855	hsa-miR-6716-3p	0	0	0	0	0	1	0	0
NM_002525	hsa-miR-4709-3p	0	0	0	0	0	1	0	0
NM_012102	hsa-miR-33a-5p	0	0	0	1	0	0	0	0
NM_001099281	hsa-miR-548t-5p	0	0	0	0	0	0	0	1
NM_005509	hsa-miR-324-5p	0	0	0	0	0	0	0	1
NM_00550	hsa-	0	1	0	0	0	0	0	0

9	miR-3124-5p								
NM_001130823	hsa-miR-5096	0	0	0	1	0	0	0	0
NM_006951	hsa-miR-4757-5p	0	0	0	1	0	0	0	0
NM_153255	hsa-miR-4540	0	0	0	1	0	0	0	0
NM_033017	hsa-miR-3194-5p	0	0	0	1	0	0	0	0
NM_005109	hsa-miR-1228-5p	0	0	0	0	0	0	0	1
NM_005109	hsa-miR-4689	0	0	0	1	0	0	0	0
NM_014603	hsa-miR-196a-5p	0	0	0	0	0	0	0	1
NM_014603	hsa-miR-196b-5p	0	0	0	0	0	0	0	1
NM_014603	hsa-miR-670	0	1	0	0	0	0	0	0
NM_001113397	hsa-miR-890	0	1	0	0	0	0	0	0
NM_001193617	hsa-miR-545-3p	0	0	0	0	0	0	0	1
NM_001193617	hsa-miR-3689b-3p	0	0	0	0	0	0	0	1
NM_001193617	hsa-miR-3689c	0	0	0	0	0	0	0	1
NM_012434	hsa-miR-578	0	0	0	0	0	0	0	1

NM_001029858	hsa-miR-4701-3p	0	0	0	0	0	0	0	1
NM_003205	hsa-miR-517a-3p	0	0	0	0	0	0	0	1
NM_003205	hsa-miR-517b-3p	0	0	0	0	0	0	0	1
NM_003205	hsa-miR-517c-3p	0	0	0	1	0	0	0	0
NM_004866	hsa-miR-660-3p	0	1	0	0	0	0	0	0
NM_004866	hsa-miR-4803	0	0	0	1	0	0	0	0
NM_025216	hsa-miR-5002-3p	0	1	0	0	0	0	0	0
NM_198594	hsa-miR-3180-3p	0	0	0	1	0	0	0	0
NM_198594	hsa-miR-5008-5p	0	0	0	0	0	0	0	1
NM_004690	hsa-miR-1229-3p	0	0	0	0	0	0	0	1
NM_178314	hsa-miR-4425	0	0	0	0	0	1	0	0
NM_002342	hsa-miR-145-5p	0	0	0	0	0	0	0	1
NM_001144952	hsa-miR-5003-3p	0	0	0	1	0	0	0	0
NM_001145443	hsa-miR-299-5p	0	0	0	1	0	0	0	0

NM_001153	hsa-miR-34a-5p	0	0	0	0	0	0	0	1
NM_012255	hsa-miR-4662a-5p	0	0	0	0	0	0	0	1
NM_014992	hsa-miR-499a-5p	0	1	0	0	0	0	0	0
NM_006363	hsa-miR-2681-3p	0	0	0	0	0	0	0	1
NM_001042784	hsa-miR-5696	0	0	0	1	0	0	0	0
NM_012465	hsa-miR-506-5p	0	0	0	0	0	0	0	1
NM_012465	hsa-miR-4326	0	0	0	1	0	0	0	0
NM_003036	hsa-miR-29c-3p	0	0	0	0	0	1	0	0
NM_001134336	hsa-miR-3683	0	0	0	0	0	0	0	1
NM_001198800	hsa-miR-617	0	0	0	1	0	0	0	0
NM_002817	hsa-miR-551a	0	0	0	0	0	0	0	1
NM_002817	hsa-miR-551b-3p	0	0	0	0	0	0	0	1
NM_019024	hsa-miR-377-3p	0	0	0	0	0	0	0	1
NM_012215	hsa-miR-1203	0	0	0	1	0	0	0	0
NM_006587	hsa-miR-194-3p	0	1	0	0	0	0	0	0
NM_005445	hsa-miR-	0	0	0	0	0	0	0	1

	586								
NM_005445	hsa-miR-4540	0	0	0	1	0	0	0	0
NM_001031695	hsa-miR-6165	0	0	0	1	0	0	0	0
NM_001134395	hsa-miR-711	0	0	0	0	0	0	0	1
NM_001134395	hsa-miR-3936	0	0	0	0	0	1	0	0
NR_029375	hsa-miR-524-3p	0	1	0	0	0	0	0	0
NR_029375	hsa-miR-3675-5p	0	0	0	1	0	0	0	0
NR_029375	hsa-miR-3150b-3p	0	0	0	1	0	0	0	0
NM_014904	hsa-miR-3688-3p	0	0	0	0	0	0	0	1
NM_130798	hsa-miR-219-2-3p	0	0	0	1	0	0	0	0
NM_130798	hsa-miR-423-5p	0	0	0	0	0	0	0	1
NM_001761	hsa-miR-4298	0	0	0	0	0	0	0	1
NM_001761	hsa-miR-4269	0	0	0	1	0	0	0	0
NM_001761	hsa-miR-4656	0	0	0	0	0	0	0	1
NM_001253726	hsa-miR-554	0	1	0	0	0	0	0	0
NM_001253726	hsa-miR-662	0	1	0	0	0	0	0	0
NM_00125	hsa-	0	0	0	0	0	0	0	1

3726	miR-4268								
NM_173515	hsa-miR-1229-5p	0	0	0	0	0	0	0	1
NR_001578	hsa-miR-143-5p	0	0	0	0	0	0	0	1
NM_000535	hsa-miR-146a-3p	0	0	0	1	0	0	0	0
NM_001081955	hsa-let-7a-5p	0	0	0	0	0	0	0	1
NM_001081955	hsa-let-7f-5p	0	0	0	0	0	0	0	1
NM_001081955	hsa-miR-4671-3p	0	0	0	1	0	0	0	0
NM_001081955	hsa-miR-4789-3p	0	0	0	0	0	0	0	1
NM_052904	hsa-miR-595	0	0	0	0	0	0	0	1
NM_052904	hsa-miR-4778-3p	0	0	0	1	0	0	0	0
NM_207420	hsa-miR-1305	0	0	0	1	0	0	0	0
NM_207420	hsa-miR-642b-3p	0	0	0	0	0	0	0	1
NM_001004	hsa-miR-5187-5p	0	0	0	0	0	1	0	0
NM_030627	hsa-miR-5187-5p	0	0	0	0	0	0	0	1
NM_014777	hsa-miR-3655	0	0	0	1	0	0	0	0
NM_15251	hsa-	0	0	0	1	0	0	0	0

2	miR-4268								
NM_001144952	hsa-miR-3160-5p	0	0	0	0	0	0	0	1
NM_001168551	hsa-miR-4778-3p	0	1	0	0	0	0	0	0
NM_016203	hsa-miR-31-3p	0	0	0	0	0	1	0	0
NM_015269	hsa-miR-3161	0	0	0	0	0	0	0	1
NM_007037	hsa-let-7c	0	0	0	0	0	1	0	0
NM_007037	hsa-miR-206	0	0	0	1	0	0	0	0
NM_007037	hsa-miR-5591-5p	0	0	0	0	0	1	0	0
NM_002726	hsa-miR-3622a-3p	0	0	0	0	0	0	0	1
NM_004606	hsa-miR-614	0	0	0	1	0	0	0	0
NM_004606	hsa-miR-4778-3p	0	1	0	0	0	0	0	0
NM_015666	hsa-miR-4664-3p	0	1	0	0	0	0	0	0
NM_001698	hsa-miR-4635	0	0	0	1	0	0	0	0
NM_001698	hsa-miR-4664-5p	0	1	0	0	0	0	0	0
NM_153281	hsa-miR-1268b	0	0	0	0	0	0	0	1
NM_01811	hsa-	0	0	0	0	0	0	0	1

7	miR-1267								
NM_018117	hsa-miR-4671-3p	0	0	0	1	0	0	0	0
NM_012102	hsa-miR-664b-3p	0	0	0	1	0	0	0	0
NM_000305	hsa-miR-29c-5p	0	0	0	0	0	0	0	1
NM_003972	hsa-miR-597	0	0	0	1	0	0	0	0
NM_003972	hsa-miR-4758-3p	0	0	0	0	0	1	0	0
NM_003972	hsa-miR-5002-3p	0	0	0	1	0	0	0	0
NM_018361	hsa-miR-499a-5p	0	1	0	0	0	0	0	0
NM_001047160	hsa-miR-337-5p	0	0	0	0	0	0	0	1
NM_015047	hsa-miR-3682-3p	0	0	0	0	0	0	0	1
NM_024874	hsa-miR-296-5p	0	0	0	0	0	0	0	1
NR_024476	hsa-miR-4760-5p	0	0	0	0	0	0	0	1
NR_027646	hsa-miR-652-3p	0	0	0	1	0	0	0	0
NR_027646	hsa-miR-676-3p	0	0	0	1	0	0	0	0
NR_027646	hsa-miR-4750-	0	0	0	1	0	0	0	0

	5p								
NR_027646	hsa-miR-5196-5p	0	0	0	0	0	0	0	1
NM_001031712	hsa-miR-26b-5p	0	0	0	0	0	0	0	1
NM_012447	hsa-miR-601	0	1	0	0	0	0	0	0
NM_012447	hsa-miR-2116-5p	0	1	0	0	0	0	0	0
NM_014946	hsa-miR-337-5p	0	0	0	1	0	0	0	0
NM_014946	hsa-miR-629-5p	0	0	0	1	0	0	0	0
NM_013441	hsa-miR-5009-5p	0	0	0	0	0	0	0	1
NM_147163	hsa-miR-132-5p	0	0	0	1	0	0	0	0
NM_052834	hsa-miR-1204	0	0	0	0	0	0	0	1
NM_006391	hsa-miR-378a-5p	0	0	0	1	0	0	0	0
NM_006391	hsa-miR-764	0	1	0	0	0	0	0	0
NM_006391	hsa-miR-3605-3p	0	0	0	0	0	0	0	1
NM_006391	hsa-miR-4765	0	0	0	0	0	0	0	1
NM_177939	hsa-miR-3689a-5p	0	0	0	0	0	0	0	1
NM_177939	hsa-miR-	0	0	0	0	0	0	0	1

	3689b-5p								
NM_177939	hsa-miR-3689e	0	0	0	0	0	0	0	1
NM_001080509	hsa-miR-890	0	1	0	0	0	0	0	0
NM_001080509	hsa-miR-4640-5p	0	0	0	0	0	0	0	1
NM_001127890	hsa-miR-184	0	0	0	0	0	0	0	1
NM_001127890	hsa-miR-5697	0	0	0	0	0	0	0	1
NR_046082	hsa-miR-767-3p	0	0	0	0	0	1	0	0
NR_046082	hsa-miR-6513-5p	0	0	0	0	0	0	0	1
NM_001227	hsa-miR-1238-3p	0	0	0	1	0	0	0	0
NM_001227	hsa-miR-6069	0	0	0	1	0	0	0	0
NM_001205254	hsa-let-7a-2-3p	0	0	0	0	0	0	0	1
NM_002039	hsa-miR-2277-3p	0	1	0	0	0	0	0	0
NM_018711	hsa-miR-5591-5p	0	1	0	0	0	0	0	0
NR_046315	hsa-miR-1972	0	0	0	1	0	0	0	0
NM_032623	hsa-miR-183-5p	0	0	0	0	0	0	0	1
NM_003100	hsa-miR-3137	0	0	0	0	0	0	0	1

NM_003100	hsa-miR-4731-5p	0	0	0	1	0	0	0	0
NM_022124	hsa-miR-1292-5p	0	0	0	0	0	0	0	1
NM_001204367	hsa-miR-675-5p	0	0	0	0	0	0	0	1
NM_001204367	hsa-miR-6075	0	0	0	1	0	0	0	0
NM_001204367	hsa-miR-6499-3p	0	0	0	0	0	0	0	1
NM_183242	hsa-miR-676-3p	0	0	0	1	0	0	0	0
NM_015090	hsa-miR-1266	0	0	0	1	0	0	0	0
NM_001017969	hsa-miR-205-3p	0	0	0	1	0	0	0	0
NM_138400	hsa-miR-675-5p	0	0	0	0	0	0	0	1
NM_153259	hsa-miR-1304-5p	0	0	0	0	0	1	0	0
NM_001190918	hsa-miR-3171	0	0	0	0	0	0	0	1
NM_001190918	hsa-miR-548ar-3p	0	0	0	0	0	1	0	0
NM_003726	hsa-miR-4691-3p	0	0	0	0	0	0	0	1
NM_001170765	hsa-miR-670	0	0	0	0	0	0	0	1
NM_001170765	hsa-miR-4701-	0	0	0	1	0	0	0	0

	3p								
NM_001170765	hsa-miR-4759	0	0	0	0	0	0	0	1
NM_198856	hsa-miR-297	0	0	0	1	0	0	0	0
NM_002967	hsa-miR-877-3p	0	0	0	1	0	0	0	0
NM_016463	hsa-miR-142-3p	0	0	0	0	0	1	0	0
NM_001199292	hsa-miR-3137	0	0	0	0	0	0	0	1
NM_014801	hsa-miR-4727-3p	0	0	0	1	0	0	0	0
NM_001080547	hsa-miR-583	0	0	0	0	0	0	0	1
NM_001080547	hsa-miR-5589-5p	0	0	0	0	0	0	0	1
NM_020389	hsa-miR-302a-5p	0	0	0	0	0	0	0	1
NM_020389	hsa-miR-374a-3p	0	0	0	0	0	0	0	1
NM_001104631	hsa-miR-4704-3p	0	0	0	0	0	0	0	1
NM_015062	hsa-miR-199a-3p	0	0	0	0	0	0	0	1
NM_015062	hsa-miR-199b-3p	0	0	0	0	0	0	0	1
NM_015062	hsa-miR-432-5p	0	0	0	0	0	0	0	1
NM_15241	hsa-	0	0	0	1	0	0	0	0

6	miR-384								
NM_152416	hsa-miR-4423-5p	0	0	0	0	0	0	0	1
NM_152416	hsa-miR-4725-5p	0	0	0	1	0	0	0	0
NM_016516	hsa-miR-4436a	0	0	0	1	0	0	0	0
NM_001621	hsa-miR-3912	0	0	0	0	0	0	0	1
NM_001621	hsa-miR-3939	0	0	0	0	0	0	0	1
NM_004272	hsa-miR-3682-3p	0	0	0	0	0	0	0	1
NM_001170765	hsa-miR-215	0	0	0	0	0	1	0	0
NM_018150	hsa-miR-1262	0	0	0	1	0	0	0	0
NM_206909	hsa-miR-342-5p	0	0	0	0	0	0	0	1
NM_206909	hsa-miR-3135b	0	0	0	1	0	0	0	0
NM_206909	hsa-miR-4684-3p	0	0	0	0	0	0	0	1
NM_001142279	hsa-miR-4778-3p	0	0	0	1	0	0	0	0
NR_003367	hsa-miR-4759	0	0	0	0	0	0	0	1
NM_001164468	hsa-miR-6720-3p	0	0	0	0	0	0	0	1
NM_00125	hsa-	0	0	0	1	0	0	0	0

6763	miR-6502-3p								
NM_024657	hsa-miR-223-5p	0	0	0	0	0	0	0	1
NM_024657	hsa-miR-571	0	1	0	0	0	0	0	0
NM_024657	hsa-miR-4420	0	0	0	0	0	0	0	1
NM_139241	hsa-miR-2277-3p	0	0	0	1	0	0	0	0
NM_139241	hsa-miR-3689f	0	0	0	1	0	0	0	0
NM_032043	hsa-let-7a-3p	0	0	0	0	0	0	0	1
NM_153832	hsa-miR-23a-5p	0	0	0	1	0	0	0	0
NM_153832	hsa-miR-3660	0	0	0	1	0	0	0	0
NM_153832	hsa-miR-5194	0	0	0	0	0	0	0	1
NM_003385	hsa-miR-181a-3p	0	0	0	1	0	0	0	0
NR_024368	hsa-miR-6076	0	0	0	1	0	0	0	0
NM_006838	hsa-miR-197-3p	0	0	0	1	0	0	0	0
NM_001144964	hsa-miR-5694	0	1	0	0	0	0	0	0
NM_001486	hsa-miR-337-5p	0	0	0	0	0	0	0	1
NM_001708	hsa-miR-25-5p	0	0	0	0	0	0	0	1
NM_177531	hsa-miR-	0	0	0	0	0	0	0	1

	1915-5p								
NM_152913	hsa-miR-338-3p	0	0	0	0	0	1	0	0
NM_001145658	hsa-miR-3605-5p	0	0	0	0	0	0	0	1
NM_021807	hsa-miR-4680-3p	0	0	0	1	0	0	0	0
NM_012310	hsa-miR-150-5p	0	1	0	0	0	0	0	0
NM_012310	hsa-miR-485-5p	0	0	0	1	0	0	0	0
NM_012310	hsa-miR-889	0	1	0	0	0	0	0	0
NR_003587	hsa-miR-3655	0	0	0	0	0	1	0	0
NM_175914	hsa-miR-214-5p	0	0	0	1	0	0	0	0
NM_175914	hsa-miR-3675-5p	0	0	0	0	0	0	0	1
NM_018430	hsa-miR-2681-5p	0	1	0	0	0	0	0	0
NM_001007527	hsa-miR-4321	0	0	0	1	0	0	0	0
NR_028041	hsa-miR-548y	0	0	0	1	0	0	0	0
NR_028041	hsa-miR-4712-3p	0	0	0	1	0	0	0	0
NR_028041	hsa-miR-548aq-5p	0	0	0	1	0	0	0	0
NM_00114	hsa-	0	0	0	0	0	0	0	1

4952	miR-3689a-5p								
NM_001144952	hsa-miR-3689b-5p	0	0	0	0	0	0	0	1
NM_001144952	hsa-miR-3689e	0	0	0	0	0	0	0	1
NM_152881	hsa-miR-591	0	0	0	1	0	0	0	0
NM_152881	hsa-miR-5003-3p	0	1	0	0	0	0	0	0
NM_032682	hsa-miR-2392	0	0	0	0	0	0	0	1
NM_000535	hsa-miR-2964a-5p	0	0	0	0	0	0	0	1
NM_018438	hsa-miR-4536-5p	0	1	0	0	0	0	0	0
NM_018438	hsa-miR-4790-3p	0	0	0	1	0	0	0	0
NM_198182	hsa-miR-490-3p	0	0	0	0	0	1	0	0
NM_198182	hsa-miR-3622a-3p	0	0	0	1	0	0	0	0
NM_152881	hsa-miR-3194-5p	0	1	0	0	0	0	0	0
NM_020400	hsa-miR-5193	0	0	0	1	0	0	0	0
NM_000618	hsa-miR-3140-5p	0	0	0	0	0	0	0	1
NM_00125	hsa-	0	0	0	0	0	1	0	0

6741	miR-6509-3p								
NM_015032	hsa-miR-219-5p	0	0	0	1	0	0	0	0
NM_006781	hsa-miR-4527	0	0	0	0	0	1	0	0
NM_015687	hsa-miR-4727-5p	0	1	0	0	0	0	0	0
NM_003477	hsa-miR-450b-3p	0	1	0	0	0	0	0	0
NM_003477	hsa-miR-4704-5p	0	0	0	0	0	0	0	1
NM_025160	hsa-miR-33b-5p	0	0	0	1	0	0	0	0
NM_052956	hsa-miR-101-3p	0	0	0	1	0	0	0	0
NR_003574	hsa-miR-141-5p	0	0	0	1	0	0	0	0
NR_003574	hsa-miR-1265	0	0	0	1	0	0	0	0
NM_005244	hsa-miR-196a-5p	0	0	0	0	0	0	0	1
NM_005244	hsa-miR-196b-5p	0	0	0	0	0	0	0	1
NM_005244	hsa-miR-1294	0	1	0	0	0	0	0	0
NM_005244	hsa-miR-3681-5p	0	0	0	0	0	0	0	1
NM_005244	hsa-miR-4680-	0	0	0	0	0	1	0	0

	3p								
NM_002854	hsa-miR-302a-3p	0	0	0	0	0	1	0	0
NM_002854	hsa-miR-890	0	0	0	0	0	0	0	1
NM_032154	hsa-miR-92a-2-5p	0	0	0	0	0	0	0	1
NM_032154	hsa-miR-5586-5p	0	0	0	1	0	0	0	0
NM_152896	hsa-miR-335-3p	0	0	0	0	0	0	0	1
NM_198460	hsa-miR-4461	0	0	0	0	0	1	0	0
NM_080841	hsa-miR-571	0	1	0	0	0	0	0	0
NM_080841	hsa-miR-4704-5p	0	0	0	0	0	0	0	1
NM_199451	hsa-miR-32-5p	0	1	0	0	0	0	0	0
NM_000947	hsa-miR-1252	0	0	0	0	0	0	0	1
NM_012121	hsa-miR-1184	0	0	0	0	0	0	0	1
NM_012121	hsa-miR-3169	0	0	0	0	0	0	0	1
NM_016480	hsa-miR-363-3p	0	0	0	1	0	0	0	0
NM_016480	hsa-miR-324-3p	0	0	0	1	0	0	0	0
NM_016480	hsa-miR-20b-3p	0	0	0	0	0	0	0	1
NM_01648	hsa-	0	0	0	0	0	1	0	0

0	miR-515-5p								
NM_016480	hsa-miR-519e-5p	0	1	0	0	0	0	0	0
NM_016480	hsa-miR-518c-5p	0	0	0	0	0	0	0	1
NM_016480	hsa-miR-516b-5p	0	0	0	1	0	0	0	0
NM_016480	hsa-miR-516a-5p	0	0	0	0	0	0	0	1
NM_016480	hsa-miR-5706	0	0	0	0	0	0	0	1
NM_032646	hsa-miR-33a-3p	0	0	0	0	0	0	0	1
NM_032646	hsa-miR-297	0	1	0	0	0	0	0	0
NM_003249	hsa-miR-15a-5p	0	0	0	0	0	0	0	1
NM_003249	hsa-miR-517-5p	0	0	0	1	0	0	0	0
NM_003249	hsa-miR-2277-3p	0	0	0	1	0	0	0	0
NM_001146337	hsa-miR-4668-3p	0	0	0	0	0	0	0	1
NM_001005731	hsa-miR-3911	0	1	0	0	0	0	0	0
NM_005778	hsa-miR-192-5p	0	0	0	0	0	0	0	1
NM_005778	hsa-miR-214-3p	0	0	0	0	0	0	0	1
NM_01498	hsa-	0	0	0	0	0	0	0	1

8	miR-150-5p								
NM_014988	hsa-miR-5581-3p	0	0	0	0	0	0	0	1
NM_001363	hsa-miR-623	0	0	0	0	0	0	0	1
NM_001363	hsa-miR-5003-3p	0	0	0	0	0	0	0	1
NM_020134	hsa-miR-3124-5p	0	0	0	0	0	0	0	1
NM_198594	hsa-miR-17-3p	0	1	0	0	0	0	0	0
NM_198594	hsa-miR-302d-5p	0	0	0	1	0	0	0	0
NM_198594	hsa-miR-5006-3p	0	0	0	1	0	0	0	0
NM_020747	hsa-miR-370	0	0	0	0	0	0	0	1
NM_003442	hsa-miR-4700-5p	0	0	0	0	0	1	0	0
NM_006207	hsa-miR-548g-5p	0	0	0	0	0	1	0	0
NM_006207	hsa-miR-548x-5p	0	0	0	0	0	1	0	0
NM_006207	hsa-miR-548aj-5p	0	0	0	0	0	1	0	0
NM_006699	hsa-miR-7-5p	0	0	0	0	0	0	0	1
NM_00375	hsa-	0	0	0	0	0	0	0	1

6	miR-1								
NM_003756	hsa-miR-1286	0	0	0	0	0	0	0	1
NM_001017969	hsa-miR-506-5p	0	0	0	1	0	0	0	0
NM_001039706	hsa-miR-4540	0	1	0	0	0	0	0	0
NM_001102406	hsa-miR-520a-5p	0	0	0	1	0	0	0	0
NM_003567	hsa-miR-499a-3p	0	0	0	1	0	0	0	0
NM_003567	hsa-miR-1238-5p	0	0	0	0	0	0	0	1
NM_001018116	hsa-miR-1181	0	0	0	1	0	0	0	0
NM_001018116	hsa-miR-5698	0	0	0	0	0	0	0	1
NM_020820	hsa-miR-624-5p	0	0	0	0	0	0	0	1
NM_020820	hsa-miR-3689a-5p	0	0	0	0	0	0	0	1
NM_020820	hsa-miR-3689b-5p	0	0	0	0	0	0	0	1
NM_020820	hsa-miR-3689e	0	0	0	0	0	0	0	1
NM_020820	hsa-miR-3689f	0	0	0	0	0	0	0	1
NM_004392	hsa-miR-141-5p	0	0	0	0	0	0	0	1
NM_004392	hsa-miR-3655	0	0	0	1	0	0	0	0

NM_014636	hsa-miR-4537	0	0	0	1	0	0	0	0
NM_014636	hsa-miR-4794	0	0	0	0	0	0	0	1
NM_153281	hsa-miR-143-3p	0	0	0	0	0	0	0	1
NM_006631	hsa-miR-143-3p	0	0	0	1	0	0	0	0
NM_006631	hsa-miR-4437	0	0	0	1	0	0	0	0
NM_014932	hsa-miR-3913-5p	0	0	0	1	0	0	0	0
NM_000433	hsa-miR-3115	0	0	0	0	0	0	0	1
NM_153609	hsa-miR-146b-5p	0	0	0	0	0	0	0	1
NM_153609	hsa-miR-1203	0	0	0	0	0	0	0	1
NM_014757	hsa-miR-608	0	0	0	0	0	0	0	1
NM_014757	hsa-miR-3680-3p	0	0	0	0	0	0	0	1
NM_014282	hsa-miR-5089-5p	0	0	0	1	0	0	0	0
NM_170692	hsa-miR-136-3p	0	0	0	1	0	0	0	0
NM_170692	hsa-miR-4731-5p	0	0	0	0	0	0	0	1
NM_006094	hsa-miR-3689a-3p	0	0	0	0	0	0	0	1

NM_206907	hsa-miR-580	0	0	0	1	0	0	0	0
NM_020405	hsa-miR-1225-5p	0	0	0	0	0	1	0	0
NM_005385	hsa-miR-505-5p	0	0	0	0	0	0	0	1
NM_001012279	hsa-miR-29a-5p	0	0	0	0	0	0	0	1
NM_001376	hsa-miR-1185-5p	0	0	0	0	0	0	0	1
NM_173490	hsa-miR-590-5p	0	0	0	0	0	0	0	1
NM_001202466	hsa-miR-3689b-3p	0	0	0	0	0	0	0	1
NM_001202466	hsa-miR-3689c	0	0	0	0	0	0	0	1
NM_003363	hsa-miR-216a-3p	0	0	0	0	0	0	0	1
NM_003363	hsa-miR-1247-3p	0	0	0	0	0	0	0	1
NM_003363	hsa-miR-3926	0	0	0	1	0	0	0	0
NM_153839	hsa-miR-662	0	1	0	0	0	0	0	0
NR_003587	hsa-miR-497-3p	0	0	0	0	0	0	0	1
NM_014272	hsa-miR-4637	0	0	0	0	0	0	0	1
NM_001135110	hsa-miR-205-3p	0	0	0	0	0	0	0	1
NM_01894	hsa-	0	0	0	1	0	0	0	0

1	miR-224-5p								
NM_139241	hsa-miR-3936	0	0	0	0	0	0	0	1
NM_054027	hsa-miR-526b-5p	0	0	0	0	0	0	0	1
NM_004959	hsa-miR-625-5p	0	0	0	1	0	0	0	0
NM_001098209	hsa-miR-4668-3p	0	0	0	0	0	1	0	0
NM_001098209	hsa-miR-4693-3p	0	0	0	0	0	0	0	1
NM_001098209	hsa-miR-4727-5p	0	0	0	0	0	0	0	1
NM_003622	hsa-miR-2053	0	0	0	0	0	1	0	0
NM_001962	hsa-miR-3162-5p	0	0	0	0	0	0	0	1
NM_031267	hsa-miR-3921	0	0	0	0	0	0	0	1
NM_020242	hsa-miR-4254	0	1	0	0	0	0	0	0
NM_148173	hsa-miR-617	0	0	0	0	0	0	0	1
NM_148173	hsa-miR-765	0	0	0	0	0	0	0	1
NM_001080517	hsa-miR-3680-3p	0	0	0	1	0	0	0	0
NM_015060	hsa-miR-3170	0	0	0	0	0	0	0	1
NM_00496	hsa-	0	0	0	0	0	0	0	1

3	miR-16-5p								
NM_004963	hsa-miR-195-5p	0	0	0	0	0	0	0	1
NM_004963	hsa-miR-4781-5p	0	1	0	0	0	0	0	0
NM_018712	hsa-miR-652-3p	0	0	0	0	0	0	0	1
NM_012112	hsa-let-7c	0	0	0	1	0	0	0	0
NM_012112	hsa-miR-4510	0	0	0	0	0	0	0	1
NM_003812	hsa-miR-3921	0	0	0	0	0	1	0	0
NM_003812	hsa-miR-4761-3p	0	0	0	1	0	0	0	0
NM_003603	hsa-miR-4787-5p	0	0	0	0	0	0	0	1
NM_016625	hsa-miR-4525	0	0	0	0	0	0	0	1
NM_001014797	hsa-miR-885-3p	0	0	0	0	0	0	0	1
NM_018711	hsa-miR-125b-2-3p	0	0	0	1	0	0	0	0
NM_018711	hsa-miR-3622a-5p	0	1	0	0	0	0	0	0
NM_018711	hsa-miR-4705	0	0	0	0	0	0	0	1
NM_024940	hsa-miR-3126-3p	0	0	0	1	0	0	0	0
NM_000621	hsa-miR-	0	0	0	0	0	0	0	1

	4760-5p								
NM_018287	hsa-miR-548n	0	0	0	0	0	0	0	1
NM_018287	hsa-miR-4803	0	0	0	1	0	0	0	0
NM_031478	hsa-miR-3138	0	0	0	0	0	1	0	0
NM_032270	hsa-miR-7-5p	0	0	0	0	0	0	0	1
NM_032270	hsa-miR-759	0	0	0	0	0	0	0	1
NM_001145443	hsa-miR-27a-3p	0	0	0	1	0	0	0	0
NM_001145443	hsa-miR-146b-3p	0	0	0	0	0	0	0	1
NM_001145443	hsa-miR-3135a	0	0	0	0	0	0	0	1
NM_001145443	hsa-miR-4423-5p	0	1	0	0	0	0	0	0
NM_022048	hsa-miR-548aq-5p	0	0	0	0	0	1	0	0
NM_006076	hsa-miR-578	0	0	0	0	0	0	0	1
NM_173508	hsa-miR-1293	0	0	0	0	0	0	0	1
NM_139281	hsa-miR-6506-3p	0	0	0	0	0	0	0	1
NM_152363	hsa-miR-614	0	1	0	0	0	0	0	0
NM_152363	hsa-miR-4703-	0	0	0	0	0	0	0	1

	3p								
NM_213604	hsa-miR-144-3p	0	1	0	0	0	0	0	0
NM_006993	hsa-miR-2116-5p	0	0	0	1	0	0	0	0
NM_006993	hsa-miR-4669	0	0	0	0	0	0	0	1
NM_018028	hsa-miR-542-3p	0	1	0	0	0	0	0	0
NM_032194	hsa-miR-31-3p	0	0	0	0	0	0	0	1
NM_003826	hsa-miR-135b-5p	0	0	0	0	0	0	0	1
NM_003826	hsa-miR-4633-5p	0	0	0	0	0	0	0	1
NM_006466	hsa-miR-1204	0	0	0	0	0	1	0	0
NM_001143850	hsa-miR-6069	0	0	0	1	0	0	0	0
NM_001244606	hsa-miR-5571-5p	0	1	0	0	0	0	0	0
NM_020894	hsa-miR-499a-5p	0	0	0	0	0	1	0	0
NM_022486	hsa-miR-4668-3p	0	0	0	1	0	0	0	0
NM_007170	hsa-miR-1292-5p	0	0	0	0	0	0	0	1
NM_007170	hsa-miR-3529-3p	0	0	0	0	0	1	0	0

NM_004849	hsa-miR-891b	0	0	0	1	0	0	0	0
NM_001037730	hsa-miR-122-5p	0	0	0	0	0	0	0	1
NM_006207	hsa-miR-597	0	0	0	0	0	0	0	1
NM_015084	hsa-miR-1471	0	0	0	0	0	0	0	1
NM_015084	hsa-miR-3177-5p	0	0	0	0	0	1	0	0
NM_015084	hsa-miR-4722-5p	0	0	0	0	0	0	0	1
NM_015052	hsa-miR-1273g-3p	0	1	0	0	0	0	0	0
NM_199076	hsa-miR-125b-2-3p	0	0	0	0	0	0	0	1
NM_199076	hsa-miR-488-3p	0	1	0	0	0	0	0	0
NM_005884	hsa-miR-614	0	0	0	0	0	1	0	0
NM_005884	hsa-miR-1471	0	0	0	0	0	1	0	0
NM_005884	hsa-miR-3177-5p	0	0	0	0	0	0	0	1
NM_017583	hsa-miR-198	0	0	0	0	0	1	0	0
NM_017583	hsa-miR-2681-3p	0	0	0	0	0	1	0	0
NM_032538	hsa-miR-4459	0	0	0	0	0	0	0	1

NM_032538	hsa-miR-5006-5p	0	0	0	1	0	0	0	0
NM_054012	hsa-miR-3144-5p	0	0	0	0	0	0	0	1
NM_016732	hsa-miR-500a-5p	0	0	0	0	0	0	0	1
NM_016732	hsa-miR-1295a	0	1	0	0	0	0	0	0
NM_016732	hsa-miR-1914-3p	0	0	0	0	0	0	0	1
NM_198353	hsa-miR-325	0	0	0	0	0	0	0	1
NM_001171990	hsa-miR-1278	0	0	0	1	0	0	0	0
NM_032172	hsa-miR-1323	0	0	0	1	0	0	0	0
NM_016252	hsa-miR-219-5p	0	0	0	1	0	0	0	0
NM_001127384	hsa-miR-325	0	1	0	0	0	0	0	0
NM_001127384	hsa-miR-2052	0	0	0	0	0	0	0	1
NM_001174066	hsa-miR-23b-5p	0	0	0	1	0	0	0	0
NM_001174066	hsa-miR-624-5p	0	0	0	1	0	0	0	0
NM_001174066	hsa-miR-4307	0	0	0	1	0	0	0	0
NM_019105	hsa-miR-488-3p	0	0	0	1	0	0	0	0
NM_181809	hsa-miR-	0	0	0	0	0	1	0	0

	122-5p								
NM_181809	hsa-miR-3614-3p	0	0	0	0	0	1	0	0
NM_181809	hsa-miR-4766-5p	0	0	0	1	0	0	0	0
NM_181809	hsa-miR-6075	0	0	0	0	0	0	0	1
NM_003887	hsa-miR-642b-5p	0	0	0	0	0	1	0	0
NM_003887	hsa-miR-4789-5p	0	0	0	0	0	0	0	1
NR_038429	hsa-miR-4321	0	0	0	1	0	0	0	0
NM_014604	hsa-miR-1293	0	0	0	0	0	0	0	1
NM_014604	hsa-miR-3675-5p	0	0	0	0	0	0	0	1
NM_014604	hsa-miR-6513-3p	0	0	0	1	0	0	0	0
NM_003159	hsa-miR-3663-5p	0	0	0	1	0	0	0	0
NM_017640	hsa-miR-100-3p	0	0	0	0	0	0	0	1
NR_003574	hsa-miR-4520a-3p	0	0	0	0	0	0	0	1
NM_004543	hsa-miR-615-5p	0	0	0	0	0	1	0	0
NM_016046	hsa-miR-5698	0	0	0	0	0	0	0	1

NM_006734	hsa-miR-4720-5p	0	0	0	1	0	0	0	0
NM_004840	hsa-miR-1207-5p	0	0	0	0	0	0	0	1
NM_004840	hsa-miR-2116-5p	0	1	0	0	0	0	0	0
NM_003750	hsa-miR-4720-5p	0	0	0	0	0	0	0	1
NM_005109	hsa-miR-4748	0	1	0	0	0	0	0	0
NM_012434	hsa-miR-518c-5p	0	0	0	1	0	0	0	0
NM_012434	hsa-miR-550b-3p	0	0	0	1	0	0	0	0
NM_012434	hsa-miR-5002-5p	0	0	0	0	0	0	0	1
NM_002451	hsa-miR-302a-3p	0	0	0	0	0	0	0	1
NM_002451	hsa-miR-424-5p	0	0	0	0	0	0	0	1
NM_005219	hsa-miR-4687-3p	0	0	0	1	0	0	0	0
NM_014878	hsa-let-7d-5p	0	0	0	0	0	0	0	1
NM_004641	hsa-miR-1298	0	0	0	0	0	1	0	0
NM_004641	hsa-miR-1294	0	0	0	0	0	0	0	1
NM_00464	hsa-	0	0	0	0	0	0	0	1

1	miR-4680-3p								
NM_020134	hsa-miR-610	0	0	0	0	0	0	0	1
NM_020134	hsa-miR-766-5p	0	0	0	0	0	1	0	0
NM_020134	hsa-miR-4692	0	0	0	1	0	0	0	0
NM_001114137	hsa-miR-1539	0	0	0	1	0	0	0	0
NM_153360	hsa-miR-3677-5p	0	0	0	0	0	0	0	1
NM_153360	hsa-miR-4746-5p	0	0	0	0	0	0	0	1
NM_194315	hsa-miR-33b-3p	0	0	0	1	0	0	0	0
NM_194315	hsa-miR-1912	0	0	0	0	0	1	0	0
NM_194315	hsa-miR-3157-5p	0	1	0	0	0	0	0	0
NM_194315	hsa-miR-3064-5p	0	0	0	1	0	0	0	0
NM_194315	hsa-miR-4776-5p	0	0	0	1	0	0	0	0
NM_194315	hsa-miR-6716-5p	0	0	0	0	0	1	0	0
NM_012309	hsa-miR-718	0	0	0	1	0	0	0	0
NM_001023570	hsa-miR-892c-	0	0	0	1	0	0	0	0

	3p								
NM_014829	hsa-miR-5589-5p	0	0	0	1	0	0	0	0
NM_004331	hsa-miR-369-3p	0	0	0	0	0	0	0	1
NM_004331	hsa-miR-6509-3p	0	0	0	0	0	0	0	1
NR_037661	hsa-miR-33b-3p	0	0	0	0	0	0	0	1
NR_037661	hsa-miR-1203	0	0	0	1	0	0	0	0
NR_037661	hsa-miR-3130-5p	0	0	0	0	0	0	0	1
NM_207408	hsa-miR-340-3p	0	0	0	0	0	0	0	1
NM_015978	hsa-miR-27a-3p	0	0	0	1	0	0	0	0
NR_027638	hsa-miR-571	0	0	0	1	0	0	0	0
NM_016231	hsa-miR-376a-3p	0	0	0	0	0	0	0	1
NR_027790	hsa-miR-4727-5p	0	0	0	1	0	0	0	0
NM_199451	hsa-miR-6072	0	1	0	0	0	0	0	0
NM_000646	hsa-miR-520h	0	0	0	0	0	0	0	1
NM_002645	hsa-miR-4752	0	0	0	1	0	0	0	0
NM_020117	hsa-miR-216a-	0	0	0	0	0	0	0	1

	5p								
NM_020117	hsa-miR-367-5p	0	0	0	0	0	0	0	1
NM_014797	hsa-miR-876-5p	0	0	0	0	0	0	0	1
NM_173508	hsa-miR-4703-3p	0	0	0	1	0	0	0	0
NM_016480	hsa-miR-22-5p	0	0	0	1	0	0	0	0
NM_016480	hsa-miR-576-5p	0	1	0	0	0	0	0	0
NM_016480	hsa-miR-3684	0	0	0	0	0	0	0	1
NM_181523	hsa-miR-4716-5p	0	0	0	0	0	0	0	1
NM_024670	hsa-miR-2277-3p	0	0	0	1	0	0	0	0
NM_024670	hsa-miR-3189-5p	0	0	0	0	0	0	0	1
NM_024670	hsa-miR-4685-3p	0	0	0	0	0	1	0	0
NM_024670	hsa-miR-4755-5p	0	0	0	0	0	1	0	0
NM_012421	hsa-miR-4725-5p	0	0	0	1	0	0	0	0
NM_012421	hsa-miR-4743-3p	0	0	0	1	0	0	0	0
NM_005433	hsa-miR-7-5p	0	0	0	0	0	1	0	0

NM_005433	hsa-miR-338-3p	0	0	0	0	0	0	0	1
NR_040109	hsa-miR-183-5p	0	0	0	0	0	0	0	1
NR_040109	hsa-miR-892c-5p	0	0	0	1	0	0	0	0
NM_017954	hsa-miR-222-5p	0	0	0	1	0	0	0	0
NM_014480	hsa-let-7i-5p	0	0	0	1	0	0	0	0
NM_024562	hsa-miR-1228-5p	0	0	0	1	0	0	0	0
NM_024562	hsa-miR-3909	0	0	0	0	0	0	0	1
NM_001815	hsa-miR-1299	0	0	0	1	0	0	0	0
NM_001815	hsa-miR-3074-5p	0	0	0	1	0	0	0	0
NM_001815	hsa-miR-676-3p	0	0	0	1	0	0	0	0
NM_001815	hsa-miR-4680-3p	0	1	0	0	0	0	0	0
NM_012112	hsa-miR-505-5p	0	0	0	0	0	0	0	1
NM_012112	hsa-miR-3129-5p	0	0	0	0	0	1	0	0
NM_012112	hsa-miR-4720-3p	0	0	0	0	0	0	0	1
NR_037185	hsa-miR-508-5p	0	0	0	0	0	0	0	1
NM_00104	hsa-	0	0	0	0	0	0	0	1

2475	miR-126-3p								
NR_024478	hsa-miR-5089-5p	0	0	0	1	0	0	0	0
NM_153265	hsa-miR-26b-5p	0	0	0	0	0	0	0	1
NM_001310	hsa-miR-367-5p	0	0	0	0	0	0	0	1
NM_017738	hsa-miR-98-5p	0	0	0	0	0	0	0	1
NM_015090	hsa-miR-6503-3p	0	0	0	0	0	0	0	1
NM_014636	hsa-miR-4787-5p	0	1	0	0	0	0	0	0
NM_001023570	hsa-miR-34a-5p	0	1	0	0	0	0	0	0
NM_017660	hsa-miR-1238-5p	0	0	0	0	0	0	0	1
NM_012281	hsa-miR-643	0	0	0	1	0	0	0	0
NM_012281	hsa-miR-1178-3p	0	0	0	0	0	1	0	0
NM_012281	hsa-miR-3200-3p	0	0	0	0	0	0	0	1
NM_012281	hsa-miR-4290	0	0	0	0	0	0	0	1
NM_030885	hsa-miR-27a-3p	0	0	0	1	0	0	0	0
NM_030885	hsa-miR-612	0	0	0	0	0	0	0	1
NM_03088	hsa-	0	0	0	0	0	0	0	1

5	miR-548m								
NM_030885	hsa-miR-548t-5p	0	0	0	0	0	0	0	1
NM_030885	hsa-miR-548az-5p	0	0	0	0	0	0	0	1
NR_027755	hsa-miR-29b-2-5p	0	1	0	0	0	0	0	0
NR_027755	hsa-miR-187-3p	0	1	0	0	0	0	0	0
NR_027755	hsa-miR-1203	0	0	0	0	0	0	0	1
NM_004798	hsa-miR-5683	0	0	0	0	0	1	0	0
NM_017553	hsa-miR-132-3p	0	0	0	0	0	1	0	0
NM_017553	hsa-miR-942	0	0	0	0	0	1	0	0
NM_017553	hsa-miR-548q	0	0	0	0	0	1	0	0
NM_001005731	hsa-miR-629-3p	0	0	0	0	0	1	0	0
NM_001005731	hsa-miR-1915-5p	0	0	0	1	0	0	0	0
NM_001083913	hsa-miR-3135b	0	0	0	1	0	0	0	0
NM_014722	hsa-miR-143-3p	0	0	0	1	0	0	0	0
NM_014722	hsa-miR-1323	0	0	0	0	0	1	0	0
NM_133444	hsa-miR-4666a-3p	0	0	0	0	0	0	0	1

NM_133444	hsa-miR-4799-3p	0	0	0	0	0	0	0	1
NM_002037	hsa-miR-610	0	0	0	1	0	0	0	0
NM_002037	hsa-miR-4525	0	0	0	1	0	0	0	0
NM_002037	hsa-miR-4705	0	0	0	0	0	0	0	1
NR_027473	hsa-miR-30d-3p	0	0	0	0	0	0	0	1
NR_027473	hsa-miR-3688-3p	0	0	0	0	0	0	0	1
NM_153223	hsa-miR-3613-5p	0	0	0	0	0	0	0	1
NM_144627	hsa-miR-2964a-5p	0	0	0	1	0	0	0	0
NM_144627	hsa-miR-4778-3p	0	0	0	0	0	0	0	1
NM_000646	hsa-miR-676-3p	0	0	0	1	0	0	0	0
NM_000646	hsa-miR-6718-5p	0	1	0	0	0	0	0	0
NM_015156	hsa-miR-1180	0	0	0	0	0	0	0	1
NM_001903	hsa-miR-154-5p	0	0	0	0	0	0	0	1
NM_001903	hsa-miR-4742-3p	0	0	0	0	0	1	0	0
NM_001903	hsa-miR-	0	0	0	0	0	0	0	1

	4760-5p								
NM_058004	hsa-let-7c	0	0	0	0	0	1	0	0
NM_058004	hsa-miR-122-5p	0	0	0	0	0	0	0	1
NM_015496	hsa-miR-3118	0	0	0	0	0	1	0	0
NM_014803	hsa-miR-4797-3p	0	0	0	1	0	0	0	0
NM_002709	hsa-miR-302a-5p	0	1	0	0	0	0	0	0
NM_002709	hsa-miR-4632-3p	0	0	0	0	0	1	0	0
NM_153228	hsa-miR-4797-3p	0	1	0	0	0	0	0	0
NM_153228	hsa-miR-5589-5p	0	0	0	0	0	0	0	1
NM_024407	hsa-miR-4477b	0	0	0	1	0	0	0	0
NR_024123	hsa-miR-2964a-5p	0	0	0	1	0	0	0	0
NM_181645	hsa-miR-3675-5p	0	0	0	0	0	0	0	1
NM_001698	hsa-miR-217	0	0	0	0	0	0	0	1
NM_001698	hsa-miR-5698	0	0	0	1	0	0	0	0
NM_014622	hsa-miR-4798-5p	0	0	0	0	0	0	0	1

NM_015084	hsa-miR-6716-3p	0	0	0	1	0	0	0	0
NM_001142650	hsa-miR-99a-5p	0	0	0	1	0	0	0	0
NM_005445	hsa-miR-23b-5p	0	1	0	0	0	0	0	0
NM_005445	hsa-miR-5587-3p	0	0	0	1	0	0	0	0
NM_016480	hsa-miR-3908	0	0	0	0	0	0	0	1
NM_016480	hsa-miR-3924	0	0	0	0	0	1	0	0
NM_016480	hsa-miR-3929	0	0	0	0	0	0	0	1
NM_016480	hsa-miR-4684-5p	0	0	0	1	0	0	0	0
NM_015315	hsa-miR-4671-3p	0	0	0	1	0	0	0	0
NR_024584	hsa-miR-4693-3p	0	0	0	0	0	0	0	1
NM_017754	hsa-miR-2116-5p	0	0	0	0	0	0	0	1
NM_014868	hsa-miR-516a-5p	0	0	0	0	0	0	0	1
NM_014868	hsa-miR-630	0	0	0	0	0	0	0	1
NM_178127	hsa-miR-520d-3p	0	0	0	0	0	0	0	1
NM_17812	hsa-	0	0	0	0	0	0	0	1

7	miR-4760-5p								
NM_178127	hsa-miR-5187-5p	0	0	0	1	0	0	0	0
NM_001130823	hsa-miR-3158-5p	0	0	0	1	0	0	0	0
NM_178314	hsa-miR-5579-3p	0	0	0	0	0	0	0	1
NM_001023570	hsa-miR-2053	0	0	0	0	0	0	0	1
NM_033389	hsa-miR-190b	0	0	0	0	0	0	0	1
NM_033389	hsa-miR-3913-5p	0	0	0	0	0	0	0	1
NM_001136225	hsa-miR-4662a-5p	0	1	0	0	0	0	0	0
NM_001142279	hsa-miR-5007-5p	0	0	0	0	0	0	0	1
NM_032043	hsa-miR-4680-3p	0	0	0	1	0	0	0	0
NM_016480	hsa-miR-6499-5p	0	1	0	0	0	0	0	0
NM_198837	hsa-miR-3939	0	0	0	0	0	0	0	1
NM_005316	hsa-miR-3939	0	0	0	1	0	0	0	0
NM_005316	hsa-miR-4752	0	0	0	1	0	0	0	0
NM_18164	hsa-	0	0	0	0	0	0	0	1

4	miR-519e-3p								
NM_181644	hsa-miR-4277	0	0	0	0	0	0	0	1
NM_022131	hsa-miR-5187-5p	0	0	0	0	0	0	0	1
NM_003887	hsa-miR-3622a-5p	0	0	0	0	0	1	0	0
NM_020760	hsa-miR-139-5p	0	1	0	0	0	0	0	0
NM_020760	hsa-miR-5694	0	0	0	1	0	0	0	0
NM_006706	hsa-miR-205-5p	0	0	0	0	0	0	0	1
NM_006706	hsa-miR-200b-5p	0	0	0	0	0	0	0	1
NM_006706	hsa-miR-660-5p	0	1	0	0	0	0	0	0
NM_001024855	hsa-miR-27a-5p	0	0	0	0	0	0	0	1
NM_001024855	hsa-miR-544a	0	0	0	0	0	0	0	1
NM_001024855	hsa-miR-4680-3p	0	1	0	0	0	0	0	0
NM_205836	hsa-miR-432-5p	0	0	0	0	0	1	0	0
NM_205836	hsa-miR-1305	0	0	0	1	0	0	0	0
NM_016282	hsa-miR-206	0	0	0	1	0	0	0	0
NM_016282	hsa-miR-	0	0	0	0	0	0	0	1

	3149								
NM_016282	hsa-miR-3160-5p	0	0	0	0	0	1	0	0
NM_178548	hsa-miR-15a-5p	0	0	0	1	0	0	0	0
NM_178548	hsa-miR-10a-5p	0	0	0	0	0	0	0	1
NM_178548	hsa-miR-15b-5p	0	0	0	1	0	0	0	0
NM_001160364	hsa-miR-941	0	0	0	0	0	0	0	1
NM_017969	hsa-miR-545-3p	0	0	0	0	0	1	0	0
NM_017969	hsa-miR-3689b-3p	0	0	0	0	0	0	0	1
NM_017969	hsa-miR-3689c	0	0	0	0	0	0	0	1
NM_198182	hsa-miR-3940-3p	0	0	0	0	0	0	0	1
NM_130843	hsa-miR-4277	0	0	0	1	0	0	0	0
NM_130843	hsa-miR-5581-3p	0	0	0	0	0	0	0	1
NM_007170	hsa-miR-5008-5p	0	0	0	1	0	0	0	0
NR_003574	hsa-miR-4436a	0	0	0	0	0	0	0	1
NM_014223	hsa-miR-676-5p	0	1	0	0	0	0	0	0
NM_003567	hsa-miR-4738-	0	0	0	0	0	0	0	1

	5p								
NM_018150	hsa-miR-1305	0	0	0	0	0	0	0	1
NM_018150	hsa-miR-3153	0	0	0	0	0	0	0	1
NR_026641	hsa-miR-1264	0	0	0	0	0	0	0	1
NM_001099281	hsa-miR-4743-3p	0	0	0	1	0	0	0	0
NM_018150	hsa-miR-126-5p	0	0	0	0	0	0	0	1
NM_002263	hsa-miR-5581-3p	0	0	0	0	0	0	0	1
NM_000747	hsa-miR-4720-3p	0	0	0	1	0	0	0	0
NM_177995	hsa-miR-3960	0	0	0	0	0	0	0	1
NM_153485	hsa-miR-2276	0	0	0	1	0	0	0	0
NM_001171832	hsa-miR-4640-5p	0	0	0	0	0	0	0	1
NM_001170765	hsa-miR-548m	0	0	0	0	0	0	0	1
NM_001130065	hsa-miR-135a-5p	0	0	0	1	0	0	0	0
NM_001130065	hsa-miR-135b-5p	0	0	0	1	0	0	0	0
NM_001130065	hsa-miR-1273e	0	0	0	1	0	0	0	0
NM_001018001	hsa-miR-	0	0	0	1	0	0	0	0

	511								
NM_001018001	hsa-miR-3683	0	0	0	0	0	0	0	1
NM_001018001	hsa-miR-5589-5p	0	0	0	1	0	0	0	0
NM_004690	hsa-miR-6718-5p	0	0	0	1	0	0	0	0
NM_016448	hsa-miR-6506-5p	0	0	0	0	0	0	0	1
NM_020405	hsa-miR-4672	0	0	0	1	0	0	0	0
NR_002451	hsa-miR-3925-3p	0	0	0	1	0	0	0	0
NM_001005731	hsa-miR-1207-5p	0	1	0	0	0	0	0	0
NM_020796	hsa-miR-1912	0	0	0	0	0	0	0	1
NM_020796	hsa-miR-5090	0	0	0	0	0	0	0	1
NM_021942	hsa-miR-125b-2-3p	0	0	0	0	0	0	0	1
NM_031217	hsa-miR-3660	0	0	0	0	0	0	0	1
NM_004516	hsa-miR-6080	0	1	0	0	0	0	0	0
NM_004796	hsa-miR-941	0	0	0	0	0	0	0	1
NM_013361	hsa-miR-21-5p	0	0	0	0	0	0	0	1
NM_018407	hsa-miR-	0	0	0	0	0	0	0	1

	892c-5p								
NM_001136017	hsa-miR-4733-3p	0	0	0	1	0	0	0	0
NM_152666	hsa-miR-1293	0	1	0	0	0	0	0	0
NM_139178	hsa-miR-660-5p	0	0	0	0	0	1	0	0
NM_182734	hsa-miR-4671-3p	0	0	0	1	0	0	0	0
NM_052905	hsa-miR-194-5p	0	0	0	1	0	0	0	0
NM_018711	hsa-miR-125b-5p	0	0	0	0	0	0	0	1
NM_018711	hsa-miR-506-5p	0	0	0	0	0	1	0	0
NM_015305	hsa-miR-3150b-3p	0	1	0	0	0	0	0	0
NM_006633	hsa-miR-187-3p	0	0	0	0	0	0	0	1
NM_004225	hsa-miR-302d-3p	0	0	0	0	0	0	0	1
NM_004225	hsa-miR-3147	0	0	0	1	0	0	0	0
NM_016224	hsa-miR-1908	0	0	0	1	0	0	0	0
NM_001242923	hsa-miR-3192	0	0	0	1	0	0	0	0
NM_000983	hsa-miR-3160-5p	0	0	0	0	0	1	0	0
NM_00098	hsa-	0	0	0	0	0	0	0	1

3	miR-4798-5p								
NM_001195017	hsa-miR-507	0	0	0	0	0	0	0	1
NM_021813	hsa-miR-625-3p	0	1	0	0	0	0	0	0
NM_018150	hsa-miR-508-3p	0	1	0	0	0	0	0	0
NM_001144952	hsa-miR-3194-3p	0	0	0	1	0	0	0	0
NM_018711	hsa-miR-125b-5p	0	0	0	0	0	0	0	1
NM_018711	hsa-miR-506-5p	0	0	0	0	0	1	0	0
NM_015305	hsa-miR-3150b-3p	0	1	0	0	0	0	0	0
NM_006633	hsa-miR-187-3p	0	0	0	0	0	0	0	1
NM_004225	hsa-miR-302d-3p	0	0	0	0	0	0	0	1
NM_004225	hsa-miR-3147	0	0	0	1	0	0	0	0
NM_016224	hsa-miR-1908	0	0	0	1	0	0	0	0
NM_001242923	hsa-miR-3192	0	0	0	1	0	0	0	0
NM_000983	hsa-miR-3160-5p	0	0	0	0	0	1	0	0
NM_000983	hsa-miR-4798-5p	0	0	0	0	0	0	0	1

NM_001195017	hsa-miR-507	0	0	0	0	0	0	0	1
NM_021813	hsa-miR-625-3p	0	1	0	0	0	0	0	0
NM_018150	hsa-miR-508-3p	0	1	0	0	0	0	0	0
NM_001144952	hsa-miR-3194-3p	0	0	0	1	0	0	0	0
NM_001038640	hsa-miR-4268	0	0	0	1	0	0	0	0
NM_001038640	hsa-miR-3909	0	0	0	1	0	0	0	0
NM_001038640	hsa-miR-499b-5p	0	0	0	1	0	0	0	0
NM_014797	hsa-miR-21-5p	0	0	0	0	0	0	0	1
NM_014797	hsa-miR-499a-5p	0	0	0	1	0	0	0	0
NM_006909	hsa-miR-2681-5p	0	0	0	1	0	0	0	0
NM_014089	hsa-miR-382-5p	0	0	0	0	0	0	0	1
NM_015906	hsa-miR-4764-5p	0	0	0	1	0	0	0	0
NM_006781	hsa-miR-4789-5p	0	0	0	0	0	0	0	1
NM_001170585	hsa-miR-4632-3p	0	0	0	0	0	1	0	0
NM_012197	hsa-miR-	0	0	0	0	0	0	0	1

	3124-5p								
NM_213649	hsa-miR-499b-5p	0	0	0	0	0	0	0	1
NM_001142807	hsa-miR-4450	0	0	0	0	0	0	0	1
NM_015215	hsa-miR-5002-5p	0	0	0	0	0	1	0	0
NM_001162529	hsa-miR-130a-5p	0	0	0	0	0	0	0	1
NM_017811	hsa-miR-548ar-3p	0	0	0	0	0	1	0	0
NM_004441	hsa-miR-4540	0	0	0	1	0	0	0	0
NM_001190918	hsa-miR-4766-5p	0	0	0	1	0	0	0	0
NM_004730	hsa-miR-1278	0	0	0	0	0	0	0	1
NM_170721	hsa-miR-218-1-3p	0	0	0	0	0	0	0	1
NM_178836	hsa-miR-27b-3p	0	0	0	1	0	0	0	0
NM_178836	hsa-miR-769-5p	0	0	0	0	0	1	0	0
NM_178836	hsa-miR-3193	0	0	0	0	0	0	0	1
NM_014992	hsa-miR-4662a-5p	0	0	0	1	0	0	0	0
NM_014728	hsa-miR-132-3p	0	0	0	0	0	0	0	1

NM_014728	hsa-miR-942	0	0	0	1	0	0	0	0
NM_014728	hsa-miR-676-3p	0	1	0	0	0	0	0	0
NM_014728	hsa-miR-4750-5p	0	0	0	0	0	0	0	1
NM_174886	hsa-miR-4783-5p	0	0	0	0	0	1	0	0
NM_015113	hsa-miR-19b-2-5p	0	0	0	0	0	0	0	1
NM_005797	hsa-miR-4680-3p	0	1	0	0	0	0	0	0
NM_014829	hsa-miR-96-5p	0	0	0	0	0	0	0	1
NM_153813	hsa-miR-1298	0	0	0	1	0	0	0	0
NM_004734	hsa-miR-1913	0	0	0	0	0	0	0	1
NM_005797	hsa-miR-3926	0	0	0	0	0	0	0	1
NM_005797	hsa-miR-4778-3p	0	0	0	0	0	1	0	0
NM_032154	hsa-miR-3150b-3p	0	0	0	1	0	0	0	0
NM_025150	hsa-miR-31-3p	0	0	0	1	0	0	0	0
NM_015062	hsa-miR-1181	0	0	0	1	0	0	0	0
NM_015194	hsa-miR-216a-	0	0	0	0	0	0	0	1

	3p								
NM_001109997	hsa-miR-4495	0	0	0	0	0	0	0	1
NR_038357	hsa-miR-587	0	0	0	0	0	0	0	1
NM_001017402	hsa-miR-889	0	0	0	1	0	0	0	0
NM_022468	hsa-miR-3131	0	0	0	0	0	0	0	1
NM_022468	hsa-miR-4788	0	0	0	1	0	0	0	0
NM_001039706	hsa-miR-95	0	0	0	0	0	0	0	1
NM_001023570	hsa-miR-572	0	1	0	0	0	0	0	0
NM_001023570	hsa-miR-668	0	1	0	0	0	0	0	0
NM_001023570	hsa-miR-3658	0	0	0	0	0	0	0	1
NM_001023570	hsa-miR-4428	0	0	0	0	0	0	0	1
NM_001146695	hsa-miR-3666	0	0	0	1	0	0	0	0
NM_001146695	hsa-miR-5582-3p	0	0	0	0	0	1	0	0
NM_002501	hsa-miR-5001-3p	0	0	0	0	0	1	0	0
NM_013302	hsa-miR-519e-3p	0	0	0	1	0	0	0	0
NM_013302	hsa-miR-33b-3p	0	0	0	0	0	1	0	0
NM_013302	hsa-miR-3157-	0	0	0	0	0	1	0	0

	5p								
NM_001099439	hsa-miR-3147	0	0	0	0	0	1	0	0
NR_039605	hsa-miR-548ar-3p	0	0	0	0	0	1	0	0
NR_039605	hsa-miR-5686	0	0	0	1	0	0	0	0
NM_006159	hsa-miR-145-3p	0	0	0	0	0	0	0	1
NM_006631	hsa-miR-1	0	0	0	0	0	0	0	1
NM_001202498	hsa-miR-125b-5p	0	0	0	0	0	0	0	1
NM_001202498	hsa-miR-610	0	1	0	0	0	0	0	0
NM_152628	hsa-miR-3936	0	0	0	0	0	0	0	1
NM_006207	hsa-miR-571	0	0	0	1	0	0	0	0
NM_001029869	hsa-miR-665	0	0	0	0	0	0	0	1
NM_006024	hsa-miR-3157-5p	0	0	0	0	0	0	0	1
NM_001043352	hsa-miR-5589-3p	0	0	0	0	0	0	0	1
NM_001183	hsa-miR-378h	0	0	0	1	0	0	0	0
NM_024792	hsa-miR-132-3p	0	0	0	0	0	0	0	1
NM_020405	hsa-miR-4498	0	0	0	0	0	0	0	1
NM_001135051	hsa-miR-	0	0	0	0	0	0	0	1

	592								
NM_181313	hsa-miR-1202	0	0	0	1	0	0	0	0
NM_181313	hsa-miR-1268b	0	1	0	0	0	0	0	0
NM_181313	hsa-miR-4689	0	1	0	0	0	0	0	0
NM_021813	hsa-miR-218-1-3p	0	0	0	1	0	0	0	0
NM_001170765	hsa-miR-100-5p	0	0	0	0	0	0	0	1
NM_006159	hsa-miR-4482-3p	0	0	0	0	0	0	0	1
NM_001199834	hsa-miR-200a-5p	0	0	0	1	0	0	0	0
NM_001199834	hsa-miR-1538	0	0	0	0	0	0	0	1
NM_013341	hsa-miR-23c	0	0	0	0	0	1	0	0
NM_001153	hsa-let-7c	0	0	0	0	0	1	0	0
NM_001153	hsa-miR-4510	0	0	0	0	0	1	0	0
NM_033165	hsa-miR-941	0	0	0	0	0	0	0	1
NM_033165	hsa-miR-6513-3p	0	0	0	0	0	1	0	0
NM_012102	hsa-miR-890	0	0	0	0	0	0	0	1
NR_028041	hsa-miR-889	0	0	0	1	0	0	0	0
NM_022373	hsa-miR-	0	1	0	0	0	0	0	0

	2392								
NM_022373	hsa-miR-6076	0	0	0	1	0	0	0	0
NM_005244	hsa-miR-432-5p	0	0	0	0	0	1	0	0
NM_005244	hsa-miR-190b	0	0	0	0	0	0	0	1
NM_005244	hsa-miR-1207-5p	0	0	0	1	0	0	0	0
NM_018711	hsa-miR-568	0	0	0	0	0	1	0	0
NM_006761	hsa-miR-6506-5p	0	0	0	0	0	1	0	0
NM_032016	hsa-miR-941	0	0	0	0	0	0	0	1
NM_005277	hsa-miR-3064-5p	0	0	0	1	0	0	0	0
NM_021116	hsa-miR-4749-5p	0	0	0	0	0	0	0	1
NM_173462	hsa-miR-4721	0	0	0	0	0	1	0	0
NM_001127715	hsa-miR-3130-5p	0	0	0	1	0	0	0	0
NM_019012	hsa-miR-924	0	0	0	1	0	0	0	0
NM_019012	hsa-miR-514b-3p	0	0	0	1	0	0	0	0
NM_002531	hsa-miR-5006-5p	0	0	0	1	0	0	0	0
NM_00125	hsa-	0	0	0	0	0	0	0	1

6630	miR-4778-3p								
NM_001173551	hsa-miR-373-5p	0	0	0	1	0	0	0	0
NM_001173551	hsa-miR-3143	0	0	0	0	0	0	0	1
NM_032043	hsa-miR-101-5p	0	0	0	0	0	0	0	1
NM_032043	hsa-miR-5698	0	0	0	1	0	0	0	0
NM_021150	hsa-miR-5193	0	0	0	0	0	0	0	1
NM_012330	hsa-miR-19b-2-5p	0	0	0	1	0	0	0	0
NM_012330	hsa-miR-92a-2-5p	0	0	0	0	0	1	0	0
NM_025160	hsa-miR-210	0	0	0	0	0	0	0	1
NM_025160	hsa-miR-3619-5p	0	0	0	1	0	0	0	0
NM_025160	hsa-miR-3622b-5p	0	0	0	1	0	0	0	0
NM_025160	hsa-miR-4766-5p	0	0	0	0	0	1	0	0
NR_026641	hsa-miR-4743-3p	0	0	0	1	0	0	0	0
NM_014777	hsa-miR-3921	0	0	0	0	0	0	0	1
NM_012465	hsa-miR-338-3p	0	0	0	0	0	0	0	1

NM_020740	hsa-miR-98-5p	0	0	0	1	0	0	0	0
NM_001184790	hsa-miR-15a-5p	0	0	0	0	0	1	0	0
NM_001184790	hsa-miR-4654	0	0	0	0	0	0	0	1
NM_000540	hsa-miR-33a-3p	0	0	0	1	0	0	0	0
NM_020760	hsa-miR-4540	0	0	0	1	0	0	0	0
NM_000937	hsa-miR-16-1-3p	0	1	0	0	0	0	0	0
NM_000937	hsa-miR-195-3p	0	0	0	1	0	0	0	0
NM_022844	hsa-miR-3681-5p	0	0	0	1	0	0	0	0
NM_022844	hsa-miR-6511a-3p	0	0	0	1	0	0	0	0
NM_003290	hsa-miR-3180-3p	0	0	0	0	0	0	0	1
NM_020129	hsa-miR-3664-3p	0	0	0	0	0	1	0	0
NM_020129	hsa-miR-4778-3p	0	0	0	0	0	1	0	0
NM_001098209	hsa-miR-138-2-3p	0	0	0	0	0	0	0	1
NM_001098209	hsa-miR-138-1-3p	0	0	0	0	0	0	0	1
NM_001098209	hsa-miR-	0	0	0	0	0	1	0	0

	638								
NM_001166448	hsa-miR-4760-5p	0	1	0	0	0	0	0	0
NR_033416	hsa-miR-374b-3p	0	0	0	0	0	1	0	0
NM_001144952	hsa-miR-1267	0	0	0	0	0	1	0	0
NM_001144952	hsa-miR-4524a-3p	0	0	0	1	0	0	0	0
NM_001145643	hsa-miR-369-5p	0	0	0	0	0	0	0	1
NM_001145354	hsa-miR-4671-3p	0	0	0	1	0	0	0	0
NM_001145354	hsa-miR-4693-3p	0	0	0	0	0	0	0	1
NM_014394	hsa-miR-1238-3p	0	0	0	1	0	0	0	0
NM_012121	hsa-miR-92a-1-5p	0	0	0	0	0	1	0	0
NM_012121	hsa-miR-6069	0	0	0	1	0	0	0	0
NM_018294	hsa-miR-943	0	0	0	0	0	0	0	1
NM_020760	hsa-miR-106b-5p	0	0	0	1	0	0	0	0
NR_024203	hsa-miR-5686	0	0	0	0	0	0	0	1
NM_004808	hsa-miR-2681-	0	0	0	0	0	0	0	1

	3p								
NM_020134	hsa-miR-4732-3p	0	0	0	1	0	0	0	0
NM_015291	hsa-miR-4725-5p	0	1	0	0	0	0	0	0
NM_001161562	hsa-miR-2117	0	0	0	1	0	0	0	0
NM_001171993	hsa-miR-520d-3p	0	0	0	0	0	0	0	1
NM_001166168	hsa-miR-6715b-5p	0	0	0	1	0	0	0	0
NM_201430	hsa-miR-3689a-3p	0	0	0	0	0	0	0	1
NM_006734	hsa-let-7b-5p	0	0	0	1	0	0	0	0
NM_006734	hsa-let-7c	0	0	0	1	0	0	0	0
NM_014844	hsa-miR-1299	0	0	0	1	0	0	0	0
NR_002451	hsa-miR-199a-3p	0	0	0	0	0	0	0	1
NR_002451	hsa-miR-199b-3p	0	0	0	0	0	0	0	1
NM_004690	hsa-miR-578	0	0	0	0	0	1	0	0
NM_003324	hsa-miR-3614-3p	0	0	0	0	0	0	0	1
NM_003324	hsa-miR-676-3p	0	0	0	1	0	0	0	0
NM_003324	hsa-miR-	0	0	0	0	0	0	0	1

	4766-5p								
NM_004440	hsa-miR-4999-5p	0	0	0	0	0	1	0	0
NM_006838	hsa-miR-448	0	0	0	0	0	0	0	1
NM_006838	hsa-miR-6513-3p	0	1	0	0	0	0	0	0
NM_004810	hsa-let-7a-3p	0	0	0	1	0	0	0	0
NM_004810	hsa-miR-924	0	0	0	0	0	0	0	1
NR_028041	hsa-miR-5580-5p	0	0	0	1	0	0	0	0
NM_002627	hsa-miR-23b-5p	0	0	0	0	0	0	0	1
NM_002627	hsa-miR-4452	0	1	0	0	0	0	0	0
NM_002154	hsa-miR-1972	0	0	0	0	0	0	0	1
NM_003622	hsa-miR-591	0	0	0	0	0	1	0	0
NM_000404	hsa-miR-3165	0	0	0	1	0	0	0	0
NM_024681	hsa-miR-1207-5p	0	0	0	1	0	0	0	0
NM_032538	hsa-miR-875-3p	0	0	0	0	0	1	0	0
NM_032538	hsa-miR-3682-3p	0	0	0	0	0	0	0	1
NM_032538	hsa-miR-3150b-	0	0	0	1	0	0	0	0

	3p								
NM_001171157	hsa-miR-377-5p	0	0	0	0	0	0	0	1
NM_001171157	hsa-miR-639	0	0	0	0	0	1	0	0
NM_001171157	hsa-miR-877-5p	0	0	0	1	0	0	0	0
NM_006207	hsa-miR-4650-5p	0	0	0	0	0	0	0	1
NM_004560	hsa-miR-6504-5p	0	0	0	0	0	0	0	1
NM_014980	hsa-miR-184	0	0	0	0	0	0	0	1
NM_014980	hsa-miR-584-3p	0	0	0	0	0	0	0	1
NM_018056	hsa-miR-4802-5p	0	0	0	0	0	0	0	1
NM_014048	hsa-miR-222-5p	0	0	0	1	0	0	0	0
NM_014048	hsa-miR-6072	0	0	0	1	0	0	0	0
NM_000937	hsa-miR-4656	0	0	0	0	0	0	0	1
NM_000937	hsa-miR-4778-3p	0	0	0	1	0	0	0	0
NM_012443	hsa-miR-376b-5p	0	0	0	1	0	0	0	0
NM_133433	hsa-miR-1180	0	0	0	0	0	0	0	1
NM_133433	hsa-miR-548aq-	0	0	0	0	0	0	0	1

	5p								
NM_001099407	hsa-miR-4798-5p	0	0	0	0	0	0	0	1
NM_014423	hsa-miR-659-3p	0	0	0	0	0	0	0	1
NM_012421	hsa-miR-3977	0	1	0	0	0	0	0	0
NM_003656	hsa-miR-3972	0	0	0	0	0	0	0	1
NM_006393	hsa-miR-19b-2-5p	0	0	0	0	0	0	0	1
NM_003887	hsa-miR-92a-2-5p	0	0	0	1	0	0	0	0
NM_003887	hsa-miR-5787	0	0	0	1	0	0	0	0
NM_001098209	hsa-miR-3162-3p	0	0	0	1	0	0	0	0
NM_001098209	hsa-miR-6514-3p	0	1	0	0	0	0	0	0
NM_014686	hsa-miR-4671-3p	0	0	0	1	0	0	0	0
NM_016529	hsa-miR-192-5p	0	0	0	0	0	0	0	1
NM_016529	hsa-miR-215	0	0	0	1	0	0	0	0
NR_015382	hsa-miR-3689a-5p	0	0	0	1	0	0	0	0
NR_015382	hsa-miR-3689b-5p	0	0	0	1	0	0	0	0

NR_015382	hsa-miR-3689e	0	0	0	1	0	0	0	0
NM_016937	hsa-miR-122-5p	0	0	0	1	0	0	0	0
NM_016937	hsa-miR-3614-3p	0	0	0	0	0	0	0	1
NM_015215	hsa-let-7i-5p	0	1	0	0	0	0	0	0
NM_002804	hsa-miR-196a-5p	0	0	0	0	0	0	0	1
NM_002804	hsa-miR-196b-5p	0	0	0	0	0	0	0	1
NM_002804	hsa-miR-1294	0	0	0	0	0	0	0	1
NM_002804	hsa-miR-5698	0	1	0	0	0	0	0	0
NM_022844	hsa-miR-92a-1-5p	0	0	0	0	0	0	0	1
NM_022844	hsa-miR-5698	0	0	0	0	0	0	0	1
NR_040073	hsa-miR-15a-3p	0	0	0	1	0	0	0	0
NM_005151	hsa-miR-184	0	0	0	1	0	0	0	0
NM_005903	hsa-miR-454-3p	0	0	0	0	0	0	0	1
NM_000747	hsa-miR-371a-5p	0	0	0	1	0	0	0	0
NM_012421	hsa-miR-6075	0	0	0	0	0	0	0	1
NM_001098209	hsa-miR-	0	0	0	1	0	0	0	0

	525-5p								
NM_007041	hsa-miR-4778-3p	0	1	0	0	0	0	0	0
NM_002248	hsa-miR-4764-5p	0	1	0	0	0	0	0	0
NM_130798	hsa-miR-508-3p	0	1	0	0	0	0	0	0
NM_022131	hsa-miR-4743-3p	0	0	0	1	0	0	0	0
NM_001164391	hsa-miR-106b-5p	0	0	0	0	0	0	0	1
NM_024622	hsa-let-7a-5p	0	0	0	0	0	1	0	0
NM_024622	hsa-let-7e-5p	0	0	0	0	0	1	0	0
NM_024622	hsa-let-7f-5p	0	0	0	0	0	1	0	0
NR_024134	hsa-miR-4268	0	0	0	0	0	0	0	1
NR_024134	hsa-miR-4722-5p	0	0	0	1	0	0	0	0
NM_005311	hsa-miR-616-5p	0	0	0	0	0	0	0	1
NM_005311	hsa-miR-651	0	0	0	0	0	0	0	1
NM_006159	hsa-miR-384	0	0	0	0	0	0	0	1
NM_003567	hsa-miR-302d-3p	0	0	0	0	0	0	0	1
NM_178584	hsa-miR-513b	0	0	0	0	0	0	0	1
NM_024670	hsa-miR-	0	0	0	0	0	1	0	0

	4693-3p								
NM_003156	hsa-miR-323b-5p	0	0	0	0	0	1	0	0
NM_012465	hsa-miR-4691-3p	0	0	0	0	0	0	0	1
NM_015196	hsa-miR-3171	0	0	0	0	0	1	0	0
NM_001378	hsa-miR-4762-5p	0	0	0	1	0	0	0	0
NR_023353	hsa-miR-16-1-3p	0	0	0	1	0	0	0	0
NR_023353	hsa-miR-16-2-3p	0	0	0	0	0	1	0	0
NR_023353	hsa-miR-324-3p	0	0	0	0	0	0	0	1
NR_023353	hsa-miR-3678-5p	0	1	0	0	0	0	0	0
NM_017772	hsa-miR-30b-5p	0	0	0	0	0	1	0	0
NM_018080	hsa-miR-4794	0	0	0	1	0	0	0	0
NR_003587	hsa-miR-1273e	0	0	0	1	0	0	0	0
NM_002616	hsa-miR-3911	0	0	0	0	0	0	0	1
NM_023034	hsa-miR-499a-3p	0	0	0	1	0	0	0	0
NM_000052	hsa-miR-127-3p	0	0	0	1	0	0	0	0
NM_139021	hsa-miR-	0	0	0	0	0	0	0	1

	4760-5p								
NM_001204299	hsa-miR-26a-5p	0	0	0	0	0	0	0	1
NM_001204299	hsa-miR-26b-5p	0	0	0	0	0	1	0	0
NM_152383	hsa-miR-149-3p	0	0	0	0	0	1	0	0
NM_152383	hsa-miR-6083	0	0	0	1	0	0	0	0
NR_027755	hsa-miR-507	0	0	0	1	0	0	0	0
NR_027755	hsa-miR-1301	0	0	0	1	0	0	0	0
NM_001098209	hsa-miR-513b	0	1	0	0	0	0	0	0
NM_001023570	hsa-miR-23b-5p	0	0	0	0	0	0	0	1
NR_023386	hsa-miR-519e-5p	0	0	0	0	0	0	0	1
NR_023386	hsa-miR-525-5p	0	0	0	0	0	0	0	1
NR_029448	hsa-miR-2052	0	0	0	0	0	0	0	1
NM_003622	hsa-miR-3944-5p	0	0	0	1	0	0	0	0
NR_015383	hsa-miR-16-5p	0	0	0	0	0	0	0	1
NR_015383	hsa-miR-195-5p	0	0	0	0	0	0	0	1
NM_002037	hsa-miR-340-3p	0	0	0	0	0	0	0	1
NM_015841	hsa-miR-	0	0	0	1	0	0	0	0

	519c-5p								
NM_015841	hsa-miR-519b-5p	0	0	0	1	0	0	0	0
NM_015841	hsa-miR-523-5p	0	0	0	1	0	0	0	0
NM_015841	hsa-miR-518e-5p	0	0	0	1	0	0	0	0
NM_015841	hsa-miR-522-5p	0	0	0	1	0	0	0	0
NM_015841	hsa-miR-519a-5p	0	0	0	1	0	0	0	0
NM_015841	hsa-miR-4761-3p	0	1	0	0	0	0	0	0
NM_015841	hsa-miR-4762-3p	0	0	0	0	0	0	0	1
NM_001031710	hsa-miR-520a-5p	0	0	0	0	0	0	0	1
NM_032043	hsa-miR-99b-5p	0	1	0	0	0	0	0	0
NM_032043	hsa-miR-3660	0	1	0	0	0	0	0	0
NM_014341	hsa-miR-4760-5p	0	0	0	0	0	0	0	1
NM_138612	hsa-miR-4781-5p	0	0	0	1	0	0	0	0
NM_021165	hsa-miR-2277-3p	0	0	0	1	0	0	0	0
NM_02116	hsa-	0	0	0	0	0	0	0	1

5	miR-3939								
NM_021165	hsa-miR-5008-5p	0	1	0	0	0	0	0	0
NM_033118	hsa-miR-450b-3p	0	1	0	0	0	0	0	0
NR_034113	hsa-miR-941	0	0	0	0	0	0	0	1
NR_038976	hsa-miR-101-3p	0	0	0	1	0	0	0	0
NR_038976	hsa-miR-382-5p	0	0	0	1	0	0	0	0
NM_001005731	hsa-miR-187-3p	0	0	0	0	0	1	0	0
NM_001005731	hsa-miR-1203	0	0	0	1	0	0	0	0
NM_001005731	hsa-miR-3130-5p	0	0	0	0	0	0	0	1
NM_001195684	hsa-miR-676-3p	0	0	0	1	0	0	0	0
NM_001195684	hsa-miR-4802-5p	0	0	0	0	0	1	0	0
NM_020820	hsa-miR-3671	0	1	0	0	0	0	0	0
NM_194286	hsa-miR-642a-5p	0	0	0	0	0	0	0	1
NM_194286	hsa-miR-662	0	0	0	1	0	0	0	0
NM_194286	hsa-miR-1285-5p	0	1	0	0	0	0	0	0
NM_00695	hsa-	0	0	0	0	0	0	0	1

8	miR-338-3p								
NM_006958	hsa-miR-578	0	0	0	1	0	0	0	0
NM_001536	hsa-miR-4760-5p	0	1	0	0	0	0	0	0
NM_020123	hsa-miR-5007-5p	0	0	0	0	0	1	0	0
NM_016653	hsa-miR-548aq-5p	0	0	0	1	0	0	0	0
NM_001185009	hsa-miR-505-3p	0	0	0	0	0	0	0	1
NM_003190	hsa-miR-1	0	0	0	1	0	0	0	0
NM_003190	hsa-miR-5047	0	0	0	0	0	0	0	1
NM_002789	hsa-miR-325	0	0	0	0	0	0	0	1
NM_003476	hsa-miR-1587	0	1	0	0	0	0	0	0
NM_003476	hsa-miR-5003-5p	0	0	0	0	0	1	0	0
NM_001142397	hsa-miR-1183	0	0	0	0	0	1	0	0
NM_001013622	hsa-miR-1471	0	0	0	1	0	0	0	0
NM_001013622	hsa-miR-4722-5p	0	0	0	1	0	0	0	0
NM_198581	hsa-miR-5591-5p	0	0	0	0	0	0	0	1
NM_005933	hsa-miR-	0	0	0	0	0	0	0	1

	642a-5p								
NM_005933	hsa-miR-3192	0	0	0	0	0	1	0	0
NM_058004	hsa-miR-3186-3p	0	1	0	0	0	0	0	0
NM_003945	hsa-miR-224-5p	0	1	0	0	0	0	0	0
NM_003945	hsa-miR-1284	0	0	0	0	0	1	0	0
NM_003945	hsa-miR-3186-3p	0	1	0	0	0	0	0	0
NM_003945	hsa-miR-4717-3p	0	0	0	1	0	0	0	0
NM_001135652	hsa-miR-1323	0	0	0	0	0	1	0	0
NM_001135652	hsa-miR-5009-5p	0	0	0	0	0	0	0	1
NM_006796	hsa-miR-1471	0	1	0	0	0	0	0	0
NM_004440	hsa-miR-2115-3p	0	1	0	0	0	0	0	0
NM_004440	hsa-miR-4656	0	0	0	0	0	0	0	1
NM_198141	hsa-miR-1298	0	0	0	0	0	0	0	1
NM_198141	hsa-miR-1539	0	0	0	1	0	0	0	0
NM_001197216	hsa-miR-3135a	0	0	0	1	0	0	0	0
NM_003887	hsa-miR-	0	0	0	0	0	1	0	0

	148a-3p								
NM_138813	hsa-miR-542-5p	0	0	0	1	0	0	0	0
NM_138813	hsa-miR-4757-5p	0	0	0	0	0	1	0	0
NM_138813	hsa-miR-5698	0	1	0	0	0	0	0	0
NM_003629	hsa-miR-4459	0	0	0	1	0	0	0	0
NM_032042	hsa-miR-1226-3p	0	0	0	0	0	1	0	0
NM_033101	hsa-miR-4482-3p	0	1	0	0	0	0	0	0
NM_001184970	hsa-miR-1178-3p	0	0	0	1	0	0	0	0
NM_001184970	hsa-miR-5581-3p	0	0	0	0	0	0	0	1
NM_022114	hsa-miR-3169	0	1	0	0	0	0	0	0
NM_015238	hsa-miR-410	0	0	0	0	0	0	0	1
NM_015238	hsa-miR-3127-3p	0	0	0	0	0	0	0	1
NM_014892	hsa-miR-21-5p	0	0	0	0	0	1	0	0
NM_001013622	hsa-miR-4743-3p	0	0	0	0	0	0	0	1
NM_001198812	hsa-miR-4742-	0	1	0	0	0	0	0	0

	3p								
NM_015318	hsa-miR-1229-5p	0	0	0	0	0	0	0	1
NM_015318	hsa-miR-5195-3p	0	1	0	0	0	0	0	0
NM_005441	hsa-miR-875-3p	0	0	0	1	0	0	0	0
NM_005441	hsa-miR-3150b-3p	0	0	0	1	0	0	0	0
NM_001145208	hsa-miR-24-1-5p	0	0	0	0	0	0	0	1
NM_052916	hsa-miR-571	0	1	0	0	0	0	0	0
NM_001698	hsa-miR-4700-5p	0	0	0	0	0	0	0	1
NR_038261	hsa-miR-1269a	0	1	0	0	0	0	0	0
NR_038261	hsa-miR-1269b	0	1	0	0	0	0	0	0
NM_014760	hsa-miR-1292-5p	0	0	0	0	0	0	0	1
NM_004840	hsa-miR-4653-5p	0	0	0	0	0	1	0	0
NM_001681	hsa-miR-1294	0	0	0	0	0	0	0	1
NM_001681	hsa-miR-3065-3p	0	1	0	0	0	0	0	0
NM_001681	hsa-miR-6502-5p	0	0	0	0	0	0	0	1

NM_004523	hsa-miR-4732-3p	0	1	0	0	0	0	0	0
NM_004523	hsa-miR-4760-5p	0	0	0	0	0	1	0	0
NM_002627	hsa-miR-3169	0	0	0	1	0	0	0	0
NM_001204517	hsa-miR-518c-3p	0	0	0	1	0	0	0	0
NM_001204517	hsa-miR-517a-3p	0	0	0	1	0	0	0	0
NM_001204517	hsa-miR-517b-3p	0	0	0	1	0	0	0	0
NM_001204517	hsa-miR-517c-3p	0	0	0	1	0	0	0	0
NM_001204517	hsa-miR-6502-3p	0	0	0	1	0	0	0	0
NM_133264	hsa-miR-342-5p	0	0	0	1	0	0	0	0
NM_133264	hsa-miR-5698	0	0	0	0	0	0	0	1
NM_014519	hsa-miR-548w	0	0	0	1	0	0	0	0
NM_001168344	hsa-miR-210	0	0	0	0	0	0	0	1
NM_001168344	hsa-miR-361-5p	0	0	0	0	0	0	0	1
NM_001142778	hsa-miR-764	0	1	0	0	0	0	0	0
NM_006877	hsa-miR-	0	0	0	0	0	0	0	1

	184								
NM_005797	hsa-miR-2054	0	0	0	0	0	1	0	0
NM_005797	hsa-miR-4540	0	0	0	1	0	0	0	0
NM_207408	hsa-miR-6721-5p	0	0	0	0	0	0	0	1
NM_012138	hsa-miR-1539	0	0	0	1	0	0	0	0
NM_004560	hsa-miR-4465	0	0	0	0	0	1	0	0
NM_004560	hsa-miR-4749-5p	0	0	0	1	0	0	0	0
NM_130811	hsa-miR-597	0	0	0	0	0	0	0	1
NM_130811	hsa-miR-891a	0	0	0	0	0	1	0	0
NM_0011099997	hsa-miR-5591-5p	0	0	0	1	0	0	0	0
NM_0011099997	hsa-miR-548az-5p	0	0	0	1	0	0	0	0
NM_000528	hsa-miR-544a	0	0	0	0	0	0	0	1
NM_0010200658	hsa-miR-548m	0	0	0	0	0	0	0	1
NM_0010200658	hsa-miR-548t-5p	0	0	0	0	0	1	0	0
NM_0011444060	hsa-miR-4712-5p	0	0	0	0	0	1	0	0
NM_0012044056	hsa-miR-548ar-	0	0	0	1	0	0	0	0

	3p								
NM_001098209	hsa-miR-4999-5p	0	1	0	0	0	0	0	0
NM_002510	hsa-miR-132-3p	0	0	0	1	0	0	0	0
NM_003274	hsa-miR-33b-3p	0	0	0	1	0	0	0	0
NM_003274	hsa-miR-4700-5p	0	0	0	0	0	0	0	1
NM_003274	hsa-miR-4737	0	0	0	0	0	0	0	1
NM_001199579	hsa-miR-188-5p	0	0	0	1	0	0	0	0
NM_001164436	hsa-miR-890	0	0	0	0	0	1	0	0
NM_002503	hsa-miR-302d-3p	0	0	0	0	0	0	0	1
NM_001104546	hsa-miR-3909	0	0	0	0	0	0	0	1
NM_138400	hsa-miR-30b-3p	0	0	0	0	0	1	0	0
NM_138400	hsa-miR-30c-1-3p	0	0	0	0	0	0	0	1
NM_033101	hsa-miR-4254	0	0	0	1	0	0	0	0
NM_033101	hsa-miR-4536-3p	0	0	0	1	0	0	0	0
NM_001815	hsa-miR-1299	0	1	0	0	0	0	0	0
NM_001098814	hsa-miR-4527	0	1	0	0	0	0	0	0

Supplementary table S2. miRNA target pairs (with measured protein fold change) with only miRNA 3' end interactions that were not detected by Targetscan context score model.

Dataset	Protein repression level (log 2 fold change)	Target mRNA	miRNA	Number of 3U3p 6-mer target sites	Number of 3U3p 7-merm8 target sites	Number of ORF3p 6-mer target sites
Baek et al (2008)	-0.254	NM_014018	miR-124	1	0	0
Baek et al (2008)	-0.264	NM_021218	miR-124	0	2	0
Baek et al (2008)	-0.465	NM_020385	miR-124	1	0	0
Baek et al (2008)	-0.03	NM_017516	miR-124	1	0	0
Baek et al (2008)	-0.105	NM_014503	miR-124	1	0	0
Baek et al (2008)	-0.193	NM_002283	miR-124	1	0	0
Baek et al (2008)	-0.163	NM_181558	miR-124	1	1	0
Baek et al (2008)	-0.102	NM_003589	miR-124	1	0	0
Baek et al (2008)	-0.074	NM_001376	miR-124	0	1	0
Baek et al (2008)	-0.06	NM_003403	miR-124	2	0	0
Baek et al (2008)	-0.348	NM_001008726	miR-124	0	1	0
Baek et al (2008)	-0.844	NM_005153	miR-124	0	2	0
Baek et al (2008)	-0.307	NM_005433	miR-124	0	2	1
Baek et al (2008)	-0.214	NM_003799	miR-124	1	0	1
Baek et al (2008)	-1.601	NM_024656	miR-124	0	1	0
Baek et al (2008)	-0.591	NM_014601	miR-124	0	1	0
Baek et al (2008)	-0.184	NM_012423	miR-124	1	0	0
Baek et al (2008)	-0.033	NM_005049	miR-124	0	1	0
Baek et al (2008)	-0.241	NM_002444	miR-124	0	1	0
Baek et al (2008)	-0.271	NM_012316	miR-181a	2	0	1
Baek et al (2008)	-0.008	NM_006135	miR-181a	1	0	1
Baek et al	-0.18	NM_012090	miR-181a	1	0	1

(2008)						
Baek et al (2008)	-0.054	NM_032864	miR-181a	1	0	0
Baek et al (2008)	-0.055	NM_032236	miR-181a	5	2	0
Baek et al (2008)	-0.464	NM_004552	miR-181a	1	0	1
Baek et al (2008)	-0.121	NM_006802	miR-181a	0	1	0
Baek et al (2008)	-0.48	NM_018188	miR-181a	3	0	0
Baek et al (2008)	-1.244	NM_017734	miR-181a	1	0	0
Baek et al (2008)	-0.098	NM_018841	miR-181a	2	0	0
Baek et al (2008)	-0.402	NM_198544	miR-181a	1	0	0
Baek et al (2008)	-0.678	NM_005717	miR-181a	1	0	0
Baek et al (2008)	-0.443	NM_005721	miR-181a	1	0	1
Baek et al (2008)	-0.253	NM_004846	miR-181a	1	0	0
Baek et al (2008)	-0.157	NM_152945	miR-181a	1	0	0
Baek et al (2008)	-0.023	NM_006190	miR-181a	2	0	0
Baek et al (2008)	-0.187	NM_000182	miR-181a	0	1	0
Baek et al (2008)	-0.083	NM_015904	miR-181a	1	1	0
Baek et al (2008)	-0.157	NM_138394	miR-181a	2	0	0
Baek et al (2008)	-0.664	NM_145261	miR-181a	1	0	0
Baek et al (2008)	-0.019	NM_005445	miR-181a	2	1	0
Baek et al (2008)	-0.296	NM_033500	miR-181a	1	0	0
Baek et al (2008)	-0.037	NM_001001392	miR-181a	3	0	0
Baek et al (2008)	-0.558	NM_006288	miR-181a	4	0	0
Baek et al (2008)	-0.406	NM_004551	miR-181a	1	0	0
Baek et al (2008)	-0.141	NM_012250	miR-181a	1	2	0
Baek et al (2008)	-0.13	NM_007178	miR-181a	4	0	0
Baek et al	-0.346	NM_001039938	miR-181a	2	0	0

(2008)						
Baek et al (2008)	-0.599	NM_001079521	miR-181a	1	1	0
Baek et al (2008)	-0.012	NM_001008726	miR-181a	1	0	1
Baek et al (2008)	-0.013	NM_003090	miR-181a	2	0	0
Baek et al (2008)	-0.178	NM_015432	miR-181a	0	2	0
Baek et al (2008)	-0.092	NM_000977	miR-181a	2	0	2
Baek et al (2008)	-0.099	NM_013975	miR-181a	1	0	0
Baek et al (2008)	-0.372	NM_078471	miR-181a	1	0	3
Baek et al (2008)	-0.005	NM_005177	miR-181a	2	0	0
Baek et al (2008)	-0.03	NM_001004333	miR-181a	1	0	0
Baek et al (2008)	-0.265	NM_032351	miR-181a	1	0	1
Baek et al (2008)	-0.395	NM_003574	miR-181a	4	0	0
Baek et al (2008)	-0.107	NM_182978	miR-181a	1	1	0
Baek et al (2008)	-0.447	NM_002911	miR-181a	1	0	1
Baek et al (2008)	-0.675	NM_015318	miR-181a	2	0	1
Baek et al (2008)	-0.206	NM_014601	miR-181a	1	0	3
Baek et al (2008)	-0.233	NM_032737	miR-181a	2	3	2
Baek et al (2008)	-0.182	NM_080552	miR-181a	3	0	0
Baek et al (2008)	-0.502	NM_030773	miR-181a	1	1	0
Baek et al (2008)	-0.028	NM_015511	miR-181a	0	1	0
Baek et al (2008)	-0.032	NM_007098	miR-181a	1	0	0
Baek et al (2008)	-0.011	NM_002872	miR-181a	0	1	0
Baek et al (2008)	-0.244	NM_030763	miR-181a	1	0	0
Selbach et al (2008)	-1	METTL7A	miR-155	3	0	0
Selbach et al (2008)	-0.97	SLC25A40	miR-155	4	0	0
Selbach et	-0.83	PPL	miR-155	1	0	0

al (2008)						
Selbach et al (2008)	-0.69	SNAP29	miR-155	4	0	1
Selbach et al (2008)	-0.69	TACSTD2	miR-155	4	0	0
Dataset	Protein repression level (log 2 fold change)	Target mRNA	miRNA	Number of 3U3p 6-mer target sites	Number of 3U3p 7-merm8 target sites	Number of ORF3p 6-mer target sites
Baek et al (2008)	-0.254	NM_014018	miR-124	1	0	0
Baek et al (2008)	-0.264	NM_021218	miR-124	0	2	0
Baek et al (2008)	-0.465	NM_020385	miR-124	1	0	0
Baek et al (2008)	-0.03	NM_017516	miR-124	1	0	0
Baek et al (2008)	-0.105	NM_014503	miR-124	1	0	0
Baek et al (2008)	-0.193	NM_002283	miR-124	1	0	0
Baek et al (2008)	-0.163	NM_181558	miR-124	1	1	0
Baek et al (2008)	-0.102	NM_003589	miR-124	1	0	0

Supplementary table S3a. Feature set (9 types of miRNA binding sites) for 1501 miRNA-target pairs used for training ANN

Pfold	site1	site2	site3	site4	site5	site6	site7	site8	site9
-0.46	0	0	0.125	0	0	0	0	0.049	0
-0.379	0	0.093	0.125	0.14	0.09	0	0.192	0.049	0
-0.061	0	0	0	0	0	0	0	0	0
-0.155	0	0	0	0	0	0	0	0	0
-0.245	0	0	0	0	0	0	0	0	0
-0.585	0	0.093	0.125	0	0.27	0	0	0	0
-0.19	0	0	0.125	0	0.09	0	0	0	0
-0.239	0	0	0	0.14	0.18	0	0	0	0
-1.058	0	0	0	0	0	0	0.096	0.049	0
-0.202	0	0	0	0	0	0	0	0	0
-0.232	0	0.093	0	0	0	0	0	0	0
-0.287	0	0	0	0	0	0	0	0	0
-0.004	0	0	0	0	0	0	0	0	0
-0.282	0	0.093	0.125	0.14	0.09	0	0	0	0
-0.16	0	0	0	0	0	0	0	0	0
-0.252	0.04	0.372	1.75	0.7	0.09	0	0.096	0	0
-0.467	0.04	0	0.25	0	0	0	0	0	0
-0.135	0	0	0	0.14	0.27	0	0.096	0.049	0

-0.501	0	0	0.125	0	0	0	0	0	0
-0.145	0	0	0	0	0	0	0	0	0
-0.175	0	0	0	0	0.09	0	0	0	0
-0.064	0	0.186	0.25	0.42	0.45	0.136	0	0	0
-0.755	0	0.279	0	0.28	0.36	0	0.096	0	0
-0.135	0	0	0	0	0.18	0.068	0	0	0
-0.069	0	0	0	0	0	0	0	0.196	0
-0.238	0	0	0	0	0	0	0	0	0
-0.181	0	0	0	0	0	0	0	0	0
-0.168	0	0.093	0.125	0.14	0	0	0	0	0
-0.464	0	0	0	0.14	0.18	0	0	0.049	0
-0.074	0	0.186	0	0	0	0	0	0	0
-0.431	0	0	0	0	0	0	0	0	0
-0.008	0	0.093	0	0	0	0	0	0.098	0
-0.212	0	0	0	0	0	0	0	0	0
-0.123	0	0	0	0	0	0	0	0	0
-0.043	0	0	0.25	0.56	0.18	0	0.192	0	0
-0.218	0	0	0	0	0	0	0	0	0
-0.115	0	0	0	0	0	0	0.096	0	0
-0.171	0	0	0	0	0	0	0	0	0.091
-0.046	0	0.093	0.125	0.14	0	0	0.096	0	0
-0.603	0	0	0.125	0	0	0	0	0	0
-1.283	0	0	0	0	0	0	0	0	0
-0.108	0	0	0	0	0	0	0	0	0
-0.326	0	0.186	0.125	0	0	0	0	0	0
-0.114	0	0	0	0	0	0	0	0	0
-0.082	0	0	0.25	0	0	0	0	0	0
-0.14	0	0	0.375	0	0	0	0	0	0
-0.146	0	0	0	0	0	0	0	0	0
-0.285	0	0	0.25	0	0	0	0	0	0.091
-0.007	0	0	0	0	0	0	0	0	0
-0.076	0	0	0	0	0	0	0	0	0
-0.061	0	0.093	0.25	0	0	0	0	0	0
-0.009	0	0	0	0	0	0	0	0	0
-0.69	0	0.279	0.125	0.14	0	0	0	0	0
-0.456	0	0	0.125	0	0	0	0	0	0
-0.235	0	0	0	0	0	0	0	0	0
-0.095	0	0	0	0.14	0	0	0.096	0	0
-0.374	0	0.186	0	0.14	0	0	0	0	0
-0.514	0	0	0.25	0	0.09	0	0	0	0
-0.464	0	0	0	0	0.09	0	0	0.049	0
-0.123	0	0	0	0	0	0	0	0	0
-0.464	0	0	0.125	0	0	0	0	0	0
-0.043	0	0	0.125	0	0.09	0	0	0	0
-0.099	0	0	0	0	0.09	0	0	0	0
-0.392	0	0.093	0.25	0	0	0	0	0	0
-0.254	0	0	0	0	0	0	0	0	0
-0.212	0	0	0	0	0.09	0	0	0.049	0
-0.745	0	0	0.125	0	0	0	0	0	0
-0.22	0	0	0	0	0	0	0	0	0

-0.026	0	0	0	0	0	0	0	0	0
-0.313	0	0	0	0	0	0	0	0	0
-0.248	0	0	0	0	0	0	0	0	0
-0.046	0	0	0.5	0	0	0.068	0	0	0
-0.43	0	0	0	0	0	0	0	0	0
-0.528	0	0	0.125	0	0	0	0	0	0
-0.123	0.04	0.093	0.25	0.42	0.72	0	0.096	0	0
-0.409	0	0.465	0.625	0.14	0.09	0	0.096	0	0
-0.193	0	0	0	0	0.09	0	0.096	0	0
-0.289	0	0	0	0.14	0	0	0	0	0.091
-0.002	0	0	0	0	0	0	0	0	0
-0.291	0	0	0	0	0	0	0	0	0
-0.044	0	0	0	0	0.36	0	0	0	0
-0.031	0	0	0	0	0	0	0	0.049	0
-0.173	0	0	0	0	0	0	0	0	0
-0.219	0	0	0	0	0	0	0	0	0
-0.18	0	0	0.125	0	0.36	0	0	0	0
-0.158	0	0	0	0	0	0	0	0	0
-0.092	0	0	0	0	0	0	0	0	0
-0.93	0	0	0	0	0	0	0	0	0
-0.101	0	0.093	0.125	0	0	0	0	0	0
-0.699	0	0	0.125	0	0	0	0	0	0
-0.022	0	0	0.125	0	0.18	0	0	0	0
-0.038	0	0.093	0	0.14	0.09	0.068	0	0	0
-0.053	0	0	0.125	0	0	0	0	0	0
-0.862	0.08	0.093	0.125	0.14	0.18	0	0.096	0	0
-0.172	0	0	0	0	0	0	0	0	0
-0.278	0	0	0	0	0	0	0	0.049	0
-0.254	0	0	0	0	0.09	0	0	0	0
-0.044	0	0	0.125	0.14	0	0	0	0	0
-0.165	0	0	0.125	0	0.09	0	0	0	0
-0.348	0	0	0	0	0	0.068	0	0	0
-0.192	0	0.186	0.75	0.28	0	0	0.288	0	0.091
-0.245	0	0	0	0.14	0	0	0	0	0
-0.143	0	0	0.125	0	0	0	0	0	0
-0.015	0	0	0.5	0.28	0.27	0	0.096	0	0
-0.197	0	0	0.25	0.14	0	0	0	0	0
-0.32	0.04	0	0.625	0.28	0	0	0	0	0
-0.038	0	0	0.125	0	0.18	0	0	0	0
-0.473	0	0	0	0	0	0	0	0	0
-0.278	0	0	0	0	0	0	0	0	0
-0.203	0	0.093	0	0	0	0	0	0	0
-0.098	0	0	0	0	0	0	0	0	0
-0.299	0	0.093	0	0	0	0	0	0	0
-0.663	0	0.093	0	0	0.72	0.068	0	0	0
-0.334	0	0.093	0	0	0.45	0.204	0	0	0
-0.015	0	0	0	0	0	0	0.096	0.049	0
-0.634	0	0	0	0	0.09	0	0	0	0
-0.176	0	0	0	0	0	0	0.096	0	0
-0.2	0	0.279	0.125	0	0.18	0	0	0.049	0

-0.287	0	0	0	0.14	0	0	0	0.049	0
-0.293	0	0.093	0.125	0	0.18	0	0	0	0
-0.135	0	0	0	0	0	0	0	0	0
-0.105	0	0	0	0.14	0	0	0	0	0
-0.23	0	0	0	0	0	0	0	0	0
-0.057	0	0	0	0	0	0	0	0	0.091
-0.209	0	0.279	0.375	0.98	0.27	0	0.288	0	0
-0.037	0	0	0	0	0	0	0	0	0
-0.003	0	0.093	0	0.14	0.18	0	0	0	0
-0.016	0	0	0.25	0	0.18	0	0	0	0
-0.239	0	0	0	0	0.09	0	0	0	0
-0.601	0.04	0	0.125	0.42	0	0	0	0	0
-0.244	0	0	0.125	0	0.27	0.068	0.096	0	0
-0.193	0	0	0	0	0	0	0	0	0
-0.798	0	0	0	0	0	0	0	0	0
-0.215	0	0.093	0.25	0	0.81	0.068	0	0	0
-0.027	0	0.093	0	0	0	0	0	0	0
-0.158	0	0	0	0	0	0	0	0	0
-0.18	0	0	0	0	0	0	0	0	0
-0.002	0	0	0.125	0	0	0	0	0	0
-0.286	0	0	0	0	0	0	0	0	0
-0.615	0	0	0	0	0	0	0	0	0
-0.46	0	0	0	0	0	0	0	0	0
-0.091	0	0	0	0	0	0	0	0	0
-0.032	0	0	0.25	0.14	0	0	0	0	0
-0.188	0	0	0	0	0	0	0	0	0
-0.02	0	0	0	0	0	0	0	0	0
-0.253	0	0	0	0	0	0	0	0	0
-0.134	0	0	0	0	0	0	0	0	0
-0.242	0	0	0	0	0	0	0	0	0
-0.249	0	0	0	0	0	0	0	0	0
-0.156	0	0	0	0	0	0	0	0	0
-0.059	0	0	0	0	0	0	0	0	0
-0.218	0	0	0	0	0	0	0	0.049	0
-0.08	0	0	0	0	0	0	0	0	0
-0.195	0	0	0	0	0	0	0	0	0
-0.131	0	0	0	0	0	0	0	0	0
-0.135	0	0.093	0.25	0	0	0	0	0	0
-0.09	0	0	0	0	0.09	0	0	0	0
-0.006	0	0	0	0	0.27	0	0	0.049	0
-0.299	0	0	0	0	0	0	0	0	0
-0.389	0	0	0	0	0	0	0	0	0
-0.302	0	0	0	0	0	0	0	0	0
-0.093	0	0	0	0	0	0	0	0	0
-0.039	0	0	0	0	0	0	0	0.049	0
-0.207	0	0.093	0	0.14	0	0	0.096	0	0
-0.086	0	0	0	0	0	0	0	0.049	0
-0.314	0	0.093	0.125	0	0.81	0.068	0.096	0.049	0
-0.017	0	0.093	0.25	0	0.18	0	0	0	0
-0.128	0	0	0.375	0	0	0	0	0	0

-0.025	0	0	0	0	0	0	0	0	0
-1.794	0	0	0	0	0	0	0	0	0
-0.115	0	0	0	0	0	0	0	0	0
-0.019	0	0	0	0	0	0	0	0	0
-0.486	0	0.093	0	0	0	0	0	0	0
-0.815	0	0.558	1.125	0.42	0.27	0.34	0.384	0.049	0
-0.286	0	0	0	0.14	0	0	0	0	0
-0.312	0	0	0	0	0	0	0	0	0
-0.578	0	0	0	0	0	0	0	0	0
-0.337	0	0	0	0	0	0	0	0	0
-0.837	0	0	0.125	0.14	0.09	0	0	0	0
-0.079	0	0	0	0	0	0	0	0	0
-0.113	0	0.279	1.5	0.14	0.09	0	0.096	0.049	0
-0.063	0	0	0.125	0	0	0	0	0	0
-0.014	0	0	0	0	0	0	0	0	0
-0.407	0	0	0	0	0	0	0	0	0
-0.195	0	0	0	0	0	0	0	0	0
-0.038	0	0	0	0	0.09	0	0.096	0	0
-0.264	0	0	0.125	0	0	0	0	0	0
-0.177	0	0	0.125	0.14	0.54	0	0	0	0
-0.284	0	0	0	0	0	0	0	0	0
-0.127	0	0	0	0	0	0	0	0	0
-0.104	0	0	0	0	0	0	0	0	0
-0.06	0	0	0	0	0	0	0	0.049	0
-0.047	0	0	0	0	0	0	0	0	0
-0.351	0	0.093	0.25	0	0	0.136	0	0	0
-0.017	0	0	0	0	0	0	0	0	0
-0.599	0	0	0	0	0	0	0.096	0	0
-0.186	0	0	0	0	0	0	0	0	0
-0.714	0	0	0.125	0.14	0	0	0	0	0
-0.068	0	0	0	0	0	0	0	0	0
-0.413	0	0	0	0	0	0	0	0	0
-0.253	0	0.186	0.25	0	0.27	0.068	0.192	0.049	0
-0.184	0	0	0	0	0	0	0.096	0	0
-0.083	0	0	0	0	0.18	0	0	0	0
-0.356	0	0	0	0	0	0	0	0	0
-0.072	0	0	0	0	0	0	0	0	0
-0.107	0	0	0	0	0	0	0	0	0
-0.248	0	0	0	0	0	0	0	0	0
-0.49	0	0	0	0	0.09	0	0	0	0
-0.047	0	0	0.25	0	0.09	0.136	0	0	0
-0.211	0	0	0	0	0.09	0	0	0	0
-0.381	0	0	0	0	0	0.068	0	0	0
-0.145	0	0	0	0	0	0	0	0	0
-0.213	0	0.093	0	0	0	0	0	0	0
-0.073	0	0	0	0	0	0	0	0	0
-0.422	0	0.093	0	0.14	0.09	0	0	0.147	0
-0.23	0	0.186	0.125	0	0	0	0	0	0
-0.227	0	0	0.125	0.14	0	0	0	0	0
-0.528	0	0	0.125	0	0	0	0	0	0

-0.226	0.04	0	0	0.28	0	0	0	0	0
-0.178	0	0	0	0	0	0	0	0	0
-0.168	0	0	0	0	0	0	0	0	0
-0.033	0	0.093	0	0.14	0.27	0	0	0	0
-0.083	0	0.093	0	0.28	0.09	0	0	0.098	0
-0.897	0	0	0.125	0	0	0	0	0	0
-0.165	0	0	0	0	0	0	0	0	0
-0.094	0	0	0	0	0	0	0	0	0
-0.905	0	0.093	0.125	0.28	0.54	0	0	0	0
-0.035	0	0.093	0.25	0	0.09	0.068	0	0.049	0
-0.806	0	0	0.125	0.14	0.18	0.068	0	0	0
-0.271	0	0	0.25	0.14	0	0	0	0	0
-0.096	0	0	0.125	0	0.18	0	0	0	0
-0.632	0	0	0	0	0.27	0	0	0	0
-0.471	0	0	0	0	0.09	0	0	0	0
-0.207	0	0.093	0	0.14	0	0	0	0	0
-0.07	0	0	0.25	0.14	0.18	0	0	0	0
-0.153	0	0	0	0	0	0	0	0	0
-0.636	0	0	0	0	0	0	0	0.098	0
-0.103	0	0	0	0	0	0	0	0	0
-0.082	0	0	0	0.14	0.09	0	0	0	0
-0.219	0	0	0.25	0.28	0.18	0.136	0.096	0	0
-0.17	0	0	0	0	0	0	0.096	0	0
-0.615	0	0	0	0	0	0	0	0	0
-0.026	0	0	0	0	0	0	0	0	0
-0.193	0	0	0.25	0	0.09	0	0	0	0.091
-0.487	0	0.093	0	0	0	0	0.096	0	0
-0.002	0	0	0	0	0.18	0	0	0.049	0
-0.182	0	0	0	0	0	0	0	0	0
-0.678	0	0	0	0	0.09	0	0	0	0
-0.052	0	0	0.125	0	0	0	0	0	0
-0.194	0	0	0.125	0	0.63	0	0	0	0
-0.606	0	0	0	0	0	0	0	0	0
-0.058	0	0	0	0	0	0	0	0	0
-0.28	0	0	0.125	0	0	0	0	0	0
-0.601	0	0	0	0.28	0.09	0	0	0	0
-0.113	0	0.372	0.625	0	0.36	0.068	0.192	0.049	0
-0.023	0	0	0	0	0.09	0	0.096	0.049	0
-0.898	0	0	0	0.14	0	0	0	0	0
-0.388	0	0	0	0	0	0	0	0	0
-0.109	0	0.279	0.25	0.84	0.54	0	0.096	0.049	0
-0.127	0	0	0	0	0	0	0	0	0
-0.058	0	0	0	0	0	0	0	0	0
-2.065	0	0	0	0	0	0	0	0	0
-0.189	0	0	0	0	0	0	0	0	0
-0.032	0	0	0	0	0.09	0	0	0	0
-0.349	0	0	0	0	0	0	0	0	0
-0.488	0	0	0	0	0	0	0	0	0
-0.648	0	0	0	0	0	0	0	0	0
-0.12	0	0	0	0	0	0	0	0	0

-0.3	0	0	0	0	0	0	0	0	0
-0.777	0	0	0.125	0	0	0	0	0	0
-0.467	0	0	0.625	0	0.36	0.068	0	0	0
-0.216	0	0	0.25	0	0	0.068	0	0	0
-0.064	0	0	0.125	0	0	0.068	0	0	0
-0.278	0	0	0	0	0	0	0	0	0
-0.198	0	0	0	0	0	0	0	0	0
-0.558	0	0	0	0	0.45	0	0	0	0
-0.401	0	0.093	0	0	0.18	0	0	0	0
-0.241	0	0	0	0	0	0	0	0	0
-0.114	0	0	0	0	0.09	0	0	0.049	0
-0.566	0	0	0	0	0.09	0	0.096	0	0
-0.136	0	0.372	0.75	0.42	0.09	0.136	0.096	0	0
-0.133	0	0.093	0.375	0	0	0	0	0	0
-0.017	0	0	0.25	0.14	0.27	0	0	0	0
-0.059	0	0	0.25	0	0.09	0	0	0	0
-0.092	0	0	0	0	0	0	0	0.049	0
-0.136	0	0	0	0	0	0	0	0	0
-0.17	0	0.093	0	0	0	0	0	0	0.091
-0.312	0	0.093	0	0	0	0	0	0.049	0
-0.416	0	0	0	0	0.18	0	0	0.098	0
-0.069	0	0	0	0	0	0	0	0	0
-0.195	0	0	0	0	0	0	0	0	0
-0.158	0	0	0	0	0	0	0	0	0
-0.018	0	0	0	0	0	0	0	0	0
-0.199	0	0.093	0.25	0.14	0	0	0	0	0
-0.332	0	0	0	0	0	0	0	0	0
-0.232	0	0.093	0.125	0	0.09	0	0	0	0
-0.226	0	0	0	0	0	0	0	0	0
-0.054	0	0	0	0	0.09	0	0	0	0
-0.1	0	0	0	0	0	0	0	0	0.091
-0.247	0	0.279	0	0.14	0.27	0	0	0	0
-0.055	0	0	0.25	0.14	0.27	0.068	0	0	0
-0.013	0	0	0.125	0	0.18	0.068	0	0.147	0
-0.07	0	0	0	0	0	0	0	0	0
-0.295	0	0	0	0	0	0	0	0	0
-0.118	0	0	0	0	0	0	0	0	0
-0.09	0	0	0.125	0	0	0.068	0	0.049	0
-0.105	0	0.093	0	0	0	0	0	0	0
-0.108	0	0	0	0	0	0	0	0	0
-0.066	0	0	0	0	0	0	0	0	0
-0.014	0	0	0	0	0	0	0	0	0.091
-0.027	0	0.093	0.125	0.14	0	0	0	0	0
-0.257	0	0	0	0	0	0	0	0	0
-0.482	0	0	0	0	0	0.068	0	0.049	0
-0.872	0	0.093	0	0	0.09	0	0	0.049	0
-0.133	0	0	0	0	0	0	0	0	0
-0.271	0	0	0	0	0.09	0.068	0	0.147	0
-0.053	0	0.093	0.125	0.14	0	0	0	0	0
-0.498	0	0.093	0	0.14	0.18	0.136	0.096	0	0

-0.167	0	0.093	0.125	0	0	0	0	0	0
-0.084	0	0	0	0	0.09	0	0	0	0
-0.721	0	0	0	0	0	0.068	0	0	0
-0.553	0	0	0.125	0	0.09	0	0	0	0
-0.211	0	0	0	0	0	0	0	0	0
-0.168	0	0	0	0	0	0	0	0	0
-0.201	0	0	0.125	0	0	0	0	0	0
-0.04	0	0	0	0.14	0	0	0	0	0
-0.11	0	0	0	0	0	0	0	0	0
-3.48	0	0	0	0.14	0	0	0	0	0
-0.281	0	0	0.125	0.14	0	0	0	0	0
-0.09	0	0	0	0	0	0	0	0	0
-0.237	0	0	0	0	0	0	0	0	0
-0.927	0	0	0.125	0.14	0	0	0	0	0
-0.216	0	0	0.125	0	0.09	0	0	0	0
-0.088	0	0	0.125	0.28	0	0	0	0	0
-0.215	0	0.186	0.25	0.28	0.09	0.136	0.192	0	0
-0.492	0	0	0.125	0	0	0	0	0	0
-0.041	0	0	0	0	0	0	0	0	0
-0.325	0	0	0	0	0.09	0	0	0.049	0
-0.291	0	0	0	0	0	0	0	0	0
-0.421	0	0	0.25	0.28	0	0	0.096	0	0
-0.133	0	0.093	0	0	0	0	0	0	0
-0.968	0	0.093	0.25	0.14	0	0	0	0	0
-0.117	0	0	0	0	0	0	0	0	0
-0.048	0.08	0.465	1	0.7	0.54	0.136	0	0	0
-0.293	0	0	0.125	0	0	0	0	0	0
-0.355	0	0	0	0	0	0	0	0	0
-0.003	0	0.093	0.375	0.84	0.18	0	0	0	0
-0.104	0.04	0.279	0.25	0.14	0.18	0	0	0	0
-0.106	0	0	0	0	0.09	0	0.096	0	0
-0.226	0	0.186	1.125	0.28	0.27	0	0	0	0
-0.803	0	0	0	0	0	0	0	0	0
-0.027	0	0	0	0	0	0	0.096	0	0
-0.101	0	0	0	0.14	0	0	0	0	0
-0.134	0	0.093	0.125	0	0	0	0	0	0
-0.071	0	0	0.125	0	0	0	0	0	0
-0.374	0	0	0	0	0	0	0	0	0
-0.03	0	0	0.125	0	0	0	0	0	0
-0.074	0	0	0	0	0	0	0	0	0
-0.006	0	0	0.125	0	0.09	0	0	0	0
-0.01	0	0	0.125	0	0	0	0	0	0
-0.101	0	0	0	0	0.09	0	0	0	0
-0.261	0	0	0	0	0	0	0	0	0
-0.245	0	0.186	0.25	0.14	0	0	0	0	0
-0.091	0	0	0	0	0	0	0	0	0
-0.242	0	0	0	0	0	0	0	0	0
-0.041	0	0.093	0	0.14	0	0	0	0	0
-0.203	0	0	0	0	0	0	0	0	0
-3.05	0	0	0	0	0	0	0	0	0

-0.173	0	0	0	0	0	0	0	0	0
-0.134	0	0	0	0	0	0	0	0	0
-0.426	0	0	0	0	0	0	0	0	0
-0.002	0	0.093	0.125	0.28	0.36	0.136	0.096	0.049	0
-1.035	0	0	0	0.14	0	0	0	0	0
-0.042	0	0	0	0	0.27	0	0	0	0
-0.317	0	0.093	0	0	0.09	0	0.096	0	0
-0.032	0	0	0	0	0	0	0	0	0
-0.251	0	0	0	0	0	0	0	0	0
-0.053	0	0.093	0	0	0	0	0	0	0
-0.048	0	0	0	0	0	0	0	0	0
-0.258	0	0	0.125	0.14	0	0	0	0	0
-0.031	0	0	0.125	0.14	0	0	0	0	0
-0.427	0	0.186	0.125	0.14	0	0	0	0	0
-0.196	0	0	0	0.14	0	0	0	0	0
-0.039	0	0.465	0.625	0.14	0.72	0	0.096	0	0
-0.415	0	0.093	0	0	0	0	0.192	0	0
-0.026	0	0	0.125	0	0	0	0	0	0
-0.894	0	0	0.125	0	0.09	0	0	0	0
-0.034	0	0	0	0	0	0	0	0	0
-0.09	0	0	0	0	0.09	0	0	0	0
-0.209	0	0.093	0	0	0	0	0	0	0
-0.183	0	0	0	0.28	0	0	0.096	0.147	0
-0.897	0	0	0	0.14	0	0	0	0.098	0
-0.498	0	0	0	0	0	0	0	0	0
-0.157	0	0.279	1.375	0.28	0.45	0	0	0.147	0
-0.036	0	0	0	0	0.09	0.068	0	0	0
-0.48	0	0	0	0	0	0	0	0	0
-0.02	0	0	0.125	0.14	0.09	0	0	0	0
-0.071	0	0	0	0	0	0	0	0	0
-0.315	0	0.093	0	0.42	0.18	0	0	0	0
-0.326	0	0	0	0	0	0	0	0	0
-0.202	0	0	0	0	0	0	0	0	0
-0.827	0	0	0	0	0	0	0	0	0
-0.285	0	0	0	0	0	0	0	0	0
-0.461	0	0.093	0	0.14	0	0	0.096	0.098	0
-0.06	0	0	0	0	0	0	0	0	0
-0.594	0	0	0.125	0.14	0	0	0	0	0
-0.228	0	0	0	0	0	0	0	0	0
-0.684	0	0	0	0	0.09	0	0	0	0
-0.573	0	0.093	0	0	0	0	0	0	0
-0.048	0	0.186	0.5	0.28	0	0	0	0	0
-0.417	0	0	0	0	0	0.068	0	0	0
-0.212	0	0	0	0	0.09	0	0	0.049	0
-0.168	0	0	0.125	0.14	0	0	0	0	0
-0.024	0	0	0	0	0	0	0	0.049	0
-0.034	0.04	0.093	0.25	0.14	0	0.068	0.096	0	0
-0.653	0	0	0.125	0	0	0	0	0	0
-0.082	0	0	0.125	0	0.18	0.068	0	0	0
-0.038	0	0	0	0	0.09	0	0	0.049	0

-0.472	0	0	0	0.14	0.18	0	0	0.049	0.091
-0.037	0	0.279	0.375	0.42	0.36	0	0	0	0
-0.844	0	0	0	0	0	0.136	0	0	0
-0.285	0	0	0.25	0.28	0	0	0.192	0	0
-0.035	0	0	0	0.14	0.09	0	0	0	0
-0.243	0	0	0	0	0	0	0	0	0
-0.038	0	0	0	0	0	0	0	0	0
-0.104	0	0.186	0.125	0.42	0.09	0	0.096	0	0
-0.751	0	0.093	0.125	0.14	0.09	0	0	0	0
-0.037	0	0	0	0	0	0	0	0	0
-0.249	0	0	0	0	0	0	0	0	0
-0.149	0	0.372	0.25	0.14	0.63	0.068	0	0	0
-0.166	0	0.093	0.125	0	0.18	0.068	0	0	0
-0.089	0	0.093	0.125	0.14	0.09	0.068	0	0	0.091
-0.022	0	0.186	0.625	0.28	0	0	0	0	0
-0.16	0	0	0	0	0	0	0	0	0
-0.241	0	0	0	0	0	0	0	0	0
-0.038	0	0	0.125	0	0	0	0	0	0
-0.071	0	0	0	0.14	0	0	0	0.098	0
-0.189	0	0	0	0	0.18	0	0	0	0
-0.035	0	0	0.25	0	0	0	0	0	0
-0.074	0	0	0	0	0.18	0	0	0	0
-0.227	0	0	0	0.14	0	0	0	0	0
-0.018	0	0	0	0	0	0	0	0	0
-0.23	0	0	0.125	0	0	0	0	0	0
-0.17	0	0	0	0	0	0	0.096	0.049	0
-0.71	0	0	0	0	0	0	0.096	0	0
-0.488	0	0	0	0	0	0	0	0	0
-0.414	0	0	0	0	0	0	0	0	0
-0.6	0	0	0	0.14	0	0	0	0	0
-0.35	0	0.093	0.125	0	0	0	0	0	0
-0.459	0	0	0	0	0	0	0	0	0
-0.432	0	0	0.125	0	0	0	0	0	0
-0.562	0	0	0	0	0	0	0	0	0
-0.149	0	0	0	0	0	0	0	0	0
-0.026	0	0	0	0	0	0	0	0.049	0
-0.18	0	0.093	0.5	0	0	0	0	0.049	0
-0.334	0	0	0	0	0.09	0	0	0	0
-0.062	0	0	0	0	0	0	0	0	0
-0.484	0	0	0	0	0	0	0	0	0
-0.531	0	0	0.375	0.28	0.18	0	0	0	0
-0.021	0	0	0.125	0.28	0	0.136	0.192	0	0
-0.695	0	0	0	0	0.18	0	0	0	0
-0.028	0	0	0	0	0	0	0	0	0
-0.507	0	0	0	0.14	0	0	0	0.049	0
-0.099	0	0.093	0.625	0	0	0	0	0	0
-0.192	0	0	0.125	0	0	0	0	0	0
-0.109	0	0	0.125	0	0.18	0	0	0	0
-0.256	0	0	0	0	0	0	0	0	0
-0.157	0	0	0.25	0	0.09	0	0	0.049	0

-0.413	0	0.093	0	0.14	0.09	0	0.096	0	0
-0.133	0	0	0.125	0	0.18	0	0	0.098	0
-0.188	0	0	0	0	0	0	0	0	0
-0.24	0	0	0	0	0	0	0	0	0
-0.301	0.04	0.093	0.125	0	0.18	0	0	0	0
-0.198	0	0	0	0	0.09	0	0	0	0
-0.133	0	0	0	0	0	0	0	0	0
-0.169	0	0	0	0	0	0	0	0	0
-0.026	0	0	0	0.14	0.36	0	0.096	0.049	0
-0.095	0	0	0	0	0	0	0.096	0	0.091
-0.026	0	0	0	0	0	0	0	0	0
-0.179	0	0	0	0	0	0	0	0	0
-0.372	0	0	0	0	0.09	0	0	0.147	0
-0.476	0	0.093	0.625	0	0.09	0	0	0	0
-0.087	0	0.093	0.5	0	0	0	0.096	0	0
-0.35	0	0	0	0	0	0	0	0	0
-0.457	0	0	0.25	0.28	0	0	0	0.049	0
-0.713	0	0	0.375	0	0	0	0.096	0	0
-0.2	0	0.093	0	0	0	0	0	0	0
-0.078	0	0.186	0.125	0	0.27	0	0	0	0
-0.319	0	0	0.125	0	0	0	0	0	0
-0.305	0	0	0	0	0	0	0	0	0
-0.727	0	0	0	0	0	0	0	0.049	0
-0.109	0	0	0.125	0	0	0	0	0	0
-0.111	0	0	0	0	0	0	0	0.098	0
-0.032	0	0	0	0	0	0	0	0	0
-0.13	0	0	0	0	0	0.068	0	0	0
-0.934	0	0	0	0	0.09	0	0	0	0
-0.387	0	0	0	0	0	0	0.096	0	0
-0.052	0	0	0	0	0	0.136	0	0.098	0
-0.787	0	0	0	0	0	0	0	0	0
-0.283	0	0.279	0.375	0.14	0.18	0	0	0.098	0
-0.327	0	0	0	0	0	0	0	0	0
-0.057	0	0	0	0	0.18	0	0	0	0
-0.21	0	0	0	0	0.27	0	0	0	0
-0.136	0	0	0	0	0	0	0	0	0
-0.041	0	0	0	0	0.09	0	0	0.049	0
-0.405	0	0	0.25	0	0	0	0.096	0.049	0
-0.411	0	0	0	0	0.36	0	0	0	0
-0.076	0	0	0.25	0	0.27	0	0	0	0
-0.043	0	0	0.375	0.56	0.72	0	0	0	0
-0.062	0	0	0	0	0	0	0	0	0
-0.035	0	0	0.5	0.28	0	0.068	0.096	0	0
-0.15	0	0.093	0.125	0.28	0.18	0	0	0	0
-0.487	0	0.372	1.625	1.12	0.18	0.068	0.192	0	0
-1.903	0	0	0	0	0	0	0	0	0
-0.122	0	0	0.125	0	0.18	0	0	0.049	0
-0.042	0	0.093	0	0.42	0.18	0	0	0.049	0
-0.465	0	0	0	0	0	0	0	0.049	0
-0.248	0	0	0	0.14	0.09	0	0	0	0

-0.132	0	0	0	0	0.18	0	0	0.049	0
-0.197	0	0.186	0.375	0.42	0.09	0	0.096	0	0
-0.443	0	0	0	0	0.09	0	0	0.049	0
-0.622	0	0	0	0	0	0	0.096	0	0
-0.272	0	0	0	0	0	0	0	0	0
-0.115	0	0.186	0.125	0	0.63	0	0	0	0
-0.049	0	0	0	0	0	0	0	0	0
-0.476	0	0	0	0	0	0	0	0	0
-0.074	0	0	0	0	0	0	0	0	0
-0.228	0	0	0	0	0	0	0	0	0
-0.039	0	0	0	0	0	0	0	0	0
-0.393	0	0	0.125	0.42	0.27	0	0	0	0
-0.2	0	0	0.5	0.28	0.54	0.068	0.096	0	0
-0.13	0	0.093	0	0	0.09	0	0	0	0
-0.029	0	0	0	0	0	0	0	0	0
-0.007	0	0	0	0.14	0.09	0	0	0	0
-0.376	0	0	0.125	0	0	0	0.288	0	0
-0.021	0	0	0.25	0.14	0.09	0.136	0	0	0
-0.066	0	0	0.25	0	0.09	0.068	0	0	0
-0.156	0	0	0	0	0	0	0	0	0
-0.07	0	0	0	0	0	0	0	0	0
-0.193	0	0	0.125	0	0	0.204	0.096	0	0
-0.156	0	0	0	0	0	0	0	0	0
-0.155	0	0	0	0	0.09	0.068	0	0	0
-0.189	0	0	0	0	0.09	0	0	0.098	0
-1.131	0	0.093	0.75	0	0.09	0	0	0	0
-0.283	0	0	0	0	0	0	0	0	0
-0.548	0	0	0	0	0.18	0.068	0	0	0
-1.461	0	0	0.125	0.14	0	0	0	0.147	0
-0.017	0	0.093	0.375	0	0	0	0	0	0
-0.264	0	0	0	0	0	0	0	0	0
-0.274	0	0.186	0.25	0.14	0	0.068	0	0	0
-0.198	0	0	0	0	0	0	0	0	0
-0.33	0	0	0	0.14	0	0	0	0	0
-0.279	0	0	0	0	0	0	0	0	0
-0.068	0	0	0	0	0	0	0	0	0
-0.081	0	0	0	0	0	0	0	0	0
-0.069	0	0.093	0.25	0	0	0.068	0	0.147	0
-0.116	0	0.093	0	0	0.09	0	0	0	0
-0.163	0	0	0	0	0	0	0	0	0
-0.003	0	0	0	0	0.09	0	0	0.098	0
-0.082	0	0	0.125	0	0	0	0	0	0
-0.195	0	0	0	0	0	0	0	0	0
-0.305	0	0	0	0	0	0	0	0	0
-0.173	0	0	0	0	0	0	0	0	0
-0.061	0	0	0.25	0	0	0	0	0	0
-0.51	0	0	0	0	0	0	0.096	0	0.091
-0.319	0	0.279	0.25	0.14	0.09	0	0.096	0	0
-0.41	0	0	0	0	0	0	0	0	0
-0.262	0	0.372	0.75	0.98	0.54	0	0.48	0	0

-0.036	0	0	0.125	0	0.18	0.068	0	0	0
-0.206	0	0	0	0	0.09	0	0	0.147	0
-0.149	0	0	0	0	0	0	0	0	0
-0.061	0	0	0	0.14	0.18	0	0	0	0
-0.702	0	0	0	0	0	0	0	0.049	0
-0.029	0.04	0	0	0	0.18	0	0	0	0
-0.023	0	0.186	0.5	0	0.18	0	0	0	0
-0.062	0	0	0.25	0	0	0	0.384	0	0
-0.295	0	0	0	0	0.09	0	0	0	0
-0.351	0	0.372	0.375	0.14	0.09	0.068	0	0.049	0
-0.308	0	0	0.125	0	0	0	0	0	0
-0.014	0	0	0	0	0	0	0	0	0
-0.111	0	0	0.125	0	0.09	0.068	0	0	0
-0.264	0	0	0	0	0	0	0.096	0	0
-0.014	0	0	0	0	0	0	0	0	0
-0.074	0	0.093	0.25	0.14	0.09	0	0	0	0
-1.045	0	0	0	0	0	0	0	0.196	0
-0.072	0	0	0	0	0.09	0	0.096	0	0
-0.437	0.04	0.093	0.25	0.56	0.36	0	0.288	0	0
-0.304	0	0.093	0.25	0.14	0.09	0	0	0	0
-0.071	0	0.093	0.25	0	0.09	0	0	0	0
-1.537	0	0.093	0.125	0.14	0.36	0	0	0.098	0
-0.125	0	0	0	0	0	0	0	0.049	0
-0.319	0	0	0.25	0	0	0.068	0	0	0
-0.215	0	0	0	0	0	0	0	0	0
-0.176	0	0.279	0	0.14	0.45	0.068	0	0	0
-0.071	0.04	0.372	0.25	0.14	0.09	0.204	0.096	0	0
-0.266	0	0	0	0	0	0	0	0	0
-0.181	0	0	0	0	0	0	0	0	0
-0.391	0	0	0	0	0	0	0	0	0
-0.174	0	0	0	0	0	0	0	0	0
-0.119	0	0.093	0.125	0.14	0.45	0.204	0	0.049	0
-0.177	0.04	0	0.125	0.14	0	0	0	0	0
-0.135	0	0	0	0.28	0.18	0.068	0.096	0	0
-0.316	0	0	0.125	0	0	0	0	0	0
-0.018	0	0	0	0	0	0	0	0	0
-0.182	0	0	0	0	0.27	0	0	0	0
-0.214	0	0.093	0	0	0	0	0	0	0
-0.033	0	0	0	0	0	0.068	0	0	0
-0.033	0	0	0	0	0	0	0	0	0
-0.145	0	0	0	0	0	0	0	0	0
-0.056	0	0	0	0	0.09	0	0	0	0
-0.412	0	0	0	0	0.18	0	0	0	0
-0.546	0	0	0	0	0	0	0	0	0
-0.216	0	0.093	0.125	0.14	0.09	0	0	0	0
-0.257	0	0	0	0.14	0	0	0	0	0
-0.25	0	0.093	0	0.14	0.27	0	0.192	0.098	0
-0.14	0	0.093	0.375	0.28	0.18	0.068	0.096	0	0
-0.169	0	0	0	0	0	0	0	0	0
-0.046	0	0	0.125	0.14	0.09	0.068	0	0	0

-0.05	0	0	0	0	0	0	0	0	0
-0.354	0	0.465	1.25	0.84	0.45	0	0	0	0
-0.129	0	0	0	0	0	0	0	0	0
-0.523	0	0	0.125	0	0	0	0	0.147	0
-0.359	0	0	0	0	0	0	0	0	0
-0.811	0	0	0	0	0	0	0	0.049	0
-0.002	0	0	0	0	0	0	0.096	0	0
-0.795	0	0	0	0	0	0	0	0	0
-0.303	0	0	0	0	0	0	0	0	0
-0.145	0	0	0	0	0	0	0.096	0	0
-0.383	0	0	0	0	0	0	0	0	0
-0.249	0	0.093	0.25	0.14	0	0	0	0	0
-0.207	0	0	0	0	0.18	0	0	0	0
-0.184	0	0	0	0.28	0.27	0.068	0.096	0.098	0
-0.014	0	0	0	0	0	0	0	0	0
-0.264	0	0	0	0.14	0	0	0	0	0
-0.014	0	0	0	0.14	0	0	0	0	0
-0.032	0	0	0	0	0	0	0	0	0
-0.329	0	0	0.375	0	0	0	0	0	0
-0.145	0	0	0	0	0	0	0	0	0
-0.208	0	0	0.125	0.14	0.36	0	0	0	0
-0.643	0	0	0.125	0.14	0.18	0	0	0	0
-0.044	0	0	0	0	0	0	0	0	0
-0.158	0	0	0	0	0.18	0	0	0	0
-0.019	0	0	0	0	0.18	0	0	0	0
-0.21	0	0	0	0	0.18	0	0	0	0
-0.258	0	0	0	0	0	0	0	0	0
-0.1	0	0	0	0	0	0	0	0	0
-0.121	0	0	0	0	0	0	0.096	0	0
-0.178	0	0	0.375	0.14	0.09	0.068	0	0	0
-0.033	0	0	0.125	0	0.18	0	0	0.098	0
-0.234	0	0	0	0	0	0	0	0.049	0
-0.161	0	0	0	0	0.27	0	0	0	0
-0.43	0	0.186	0.125	0	0.09	0	0.096	0.049	0
-0.31	0	0	0	0	0	0.068	0.096	0	0
-0.073	0	0	0	0	0	0	0	0	0
-0.53	0	0	0	0	0	0	0	0	0
-0.491	0	0	0	0	0	0	0	0	0
-0.614	0	0	0.375	0.14	0.27	0.136	0	0	0
-0.015	0	0	0	0	0	0	0	0	0
-0.268	0	0.372	0.5	0.84	0.63	0	0	0	0
-0.15	0	0	0	0	0	0	0	0	0
-0.061	0	0	0.125	0	0	0	0	0	0
-0.272	0	0	0	0.14	0	0	0	0.098	0
-0.361	0	0	0	0.14	0	0	0	0	0
-0.009	0	0.093	0	0.14	0.18	0	0	0	0
-0.128	0	0	0	0	0.36	0	0	0	0
-0.015	0	0	0	0.14	0	0	0	0	0
-0.061	0	0.093	0	0	0.27	0	0	0	0
-0.222	0	0	0	0	0.09	0	0	0	0

-0.1	0	0	0	0	0	0	0	0	0
-0.271	0	0	0.125	0.14	0.09	0	0.096	0	0
-0.183	0	0	0.125	0.14	0.09	0	0	0	0
-0.116	0	0.093	0.125	0.14	0.27	0	0.096	0	0
-0.416	0	0	0	0	0	0	0	0	0
-0.058	0	0	0	0	0	0	0	0	0
-0.048	0	0	0	0	0	0	0	0	0
-0.931	0	0	0	0	0	0	0	0	0
-0.021	0	0.093	0	0	0	0.136	0	0	0
-0.697	0	0	0.375	0	0	0	0	0	0
-0.088	0	0	0	0.14	0.09	0	0	0	0
-0.16	0	0	0	0	0	0	0	0	0
-0.155	0	0	0.125	0.14	0	0	0	0	0
-0.294	0	0.093	0	0	0	0	0	0	0
-0.274	0	0.093	0.25	0.14	0.27	0	0.096	0	0
-0.412	0	0	0.125	0	0.09	0.136	0	0	0
-0.018	0	0	0	0	0	0	0	0	0
-0.017	0	0	0	0	0	0	0	0	0
-0.151	0	0	0	0	0	0	0	0.049	0
-0.088	0	0	0	0	0	0	0	0	0
-0.016	0	0	0	0	0	0	0	0	0
-0.228	0	0	0	0	0	0	0	0	0
-0.068	0	0	0.25	0	0.09	0	0	0	0
-0.603	0	0	0	0	0	0	0	0	0
-0.126	0	0	0.25	0.56	0.27	0	0.288	0	0
-0.241	0	0	0.25	0.28	0.18	0	0.096	0	0
-0.443	0	0.093	0.125	0.28	0.09	0	0	0.147	0
-0.521	0	0.093	0.375	0.28	0.18	0	0	0	0
-0.05	0	0	0	0	0	0	0	0	0
-0.598	0	0.093	0	0.14	0	0	0	0	0
-0.098	0	0.093	0.25	0.56	0.18	0	0	0	0
-0.313	0	0	0	0	0	0	0.096	0	0
-0.438	0	0.093	0	0.28	0	0	0	0	0
-0.639	0	0	0	0	0.36	0	0.096	0	0
-2.33	0	0	0.25	0.14	0	0	0	0	0
-0.188	0.04	0	0.125	0.14	0	0	0	0	0
-0.603	0	0	0	0	0.09	0	0	0	0
-0.097	0	0	0	0	0	0	0	0	0
-0.145	0	0.093	0.375	0.28	0	0	0	0	0
-0.005	0.04	0.093	0.125	0	0	0	0	0	0
-0.256	0	0	0.125	0	0	0	0	0	0
-0.002	0	0	0.5	0.14	0.09	0	0.096	0	0
-0.127	0	0	0	0	0	0	0	0.098	0
-0.072	0	0	0	0	0.09	0	0	0	0
-0.314	0	0	0	0	0	0	0	0	0
-0.057	0	0	0.125	0	0	0	0.096	0	0
-0.502	0	0	0	0	0.45	0.068	0	0	0
-0.257	0	0	0.5	0.42	0.18	0	0	0	0
-0.099	0	0.186	0.25	0	0.18	0	0	0.049	0.091
-0.048	0.08	0	0	0	0	0	0	0	0

-0.094	0	0.465	0.125	0	0.54	0	0.096	0	0
-0.083	0	0	0	0	0.09	0.068	0	0	0
-0.082	0	0	0	0	0	0	0	0	0
-0.155	0	0	0	0	0.09	0	0.096	0	0
-0.274	0	0	0	0	0	0	0	0	0
-0.149	0	0.093	0	0	0.09	0	0	0.049	0
-0.101	0	0	0	0	0	0	0	0	0
-0.037	0	0	0	0	0	0	0	0	0
-0.098	0	0	0	0	0.18	0	0	0	0
-0.035	0	0	0.125	0	0	0	0	0	0
-0.232	0	0	0.125	0	0	0	0	0	0
-0.037	0	0.093	0.125	0	0	0	0	0	0
-0.065	0	0	0	0.14	0.27	0.068	0	0	0
-0.112	0	0	0	0.14	0.36	0	0.096	0.147	0
-0.31	0	0.465	0.375	0.14	0.27	0.068	0.096	0	0.091
-0.06	0	0	0	0	0.18	0	0	0	0
-0.057	0	0	0	0	0	0	0	0	0
-0.099	0	0	0.125	0.28	0	0	0	0	0
-0.239	0	0	0.125	0.14	0	0.068	0	0.049	0.091
-0.002	0	0	0	0	0	0	0	0	0
-0.139	0	0	0.125	0	0	0	0	0	0
-0.151	0	0	0	0	0.27	0	0	0	0
-0.021	0	0	0	0	0	0	0	0	0
-0.233	0	0	0	0	0	0	0	0	0
-0.055	0	0	0	0	0.45	0.136	0	0	0
-0.028	0	0	0	0	0.09	0.068	0	0	0
-0.057	0	0	0	0	0	0	0	0	0
-0.215	0	0	0	0	0	0	0	0	0
-0.039	0	0	0	0	0	0	0.096	0	0
-0.164	0	0	0.125	0	0	0	0	0	0
-0.316	0	0	0.125	0	0	0	0	0	0
-0.122	0	0	0.625	0.56	0.27	0.136	0.096	0.049	0
-0.102	0	0.093	0	0	0	0	0	0	0
-0.005	0	0	0	0	0	0	0	0	0
-0.499	0	0	0	0	0	0	0	0.098	0
-0.607	0	0	0.125	0	0	0	0	0	0
-0.137	0	0	0.125	0	0	0	0	0.147	0
-0.397	0	0	0	0	0	0	0	0	0
-0.235	0	0	0	0	0	0	0	0	0
-0.14	0	0	0.375	0	0	0	0	0	0
-0.098	0	0	0.125	0	0	0	0.096	0	0
-0.102	0	0	0.125	0	0.09	0	0	0	0
-0.231	0	0	0.125	0.14	0	0	0	0	0
-0.857	0	0	0	0	0	0	0	0	0
-0.268	0	0	0	0	0	0	0	0	0
-0.492	0	0	0	0	0	0	0	0	0
-0.077	0	0	0	0	0.09	0	0	0	0
-0.012	0	0	0	0	0	0	0	0	0
-0.377	0	0	0	0	0	0	0	0	0
-0.396	0	0.093	0	0	0	0	0	0	0

-0.048	0	0.093	0.125	0.28	0.27	0	0.096	0	0
-0.488	0	0.093	0	0	0	0	0	0	0.091
-0.536	0	0	0.25	0	0	0	0	0	0
-0.818	0	0	0	0.42	0	0	0	0	0
-0.333	0	0	0.125	0.14	0.09	0	0	0.049	0
-0.166	0	0	0	0	0	0	0	0	0
-0.154	0	0	0	0	0	0	0	0	0
-0.611	0	0	0	0	0	0	0	0	0
-0.1	0	0.093	0.75	0.42	0	0.068	0	0.049	0
-0.174	0	0.093	0.5	0	0.27	0	0	0.049	0
-0.93	0	0	0	0	0	0	0	0	0
-0.066	0	0	0.125	0	0	0	0	0	0
-0.09	0	0	0.25	0	0	0	0	0.049	0
-0.004	0	0.093	0	0	0.27	0	0.096	0	0
-0.64	0	0.093	0	0	0.36	0	0.096	0	0
-0.125	0	0	0.125	0	0	0	0	0	0
-0.57	0	0	0.125	0	0	0	0	0	0
-0.095	0	0.093	0	0.14	0.45	0	0	0	0
-0.036	0	0	0	0	0.09	0	0	0	0
-0.102	0	0	0	0	0	0	0	0	0
-0.042	0	0.093	0	0	0	0	0	0	0
-0.069	0	0	0	0	0	0	0	0	0
-0.102	0	0	0.125	0	0	0	0	0	0
-0.201	0	0	0	0	0	0.136	0	0	0
-0.03	0	0	0	0	0.09	0.068	0	0	0
-0.232	0	0	0	0	0	0	0	0	0
-0.252	0	0	0	0	0	0	0	0	0
-0.055	0	0	0.125	0	0	0	0	0	0
-0.139	0	0	0	0	0	0	0	0	0
-0.252	0	0.093	0.125	0	0	0	0	0	0
-0.022	0	0	0	0	0.09	0.068	0	0.049	0
-0.564	0	0	0	0	0	0	0	0	0
-0.006	0	0.093	0	0	0.09	0	0	0.098	0
-0.788	0	0.093	0	0	0	0	0	0.049	0
-0.471	0	0	0	0	0	0	0	0	0
-0.278	0	0.093	0.125	0	0.09	0.068	0	0	0
-0.484	0	0	0.125	0	0	0	0	0	0
-0.011	0	0	0	0	0	0	0	0	0
-0.227	0	0.093	0.125	0	0.18	0	0	0	0
-0.09	0	0	0	0	0	0	0	0	0
-0.66	0	0	0	0	0	0	0	0	0
-0.218	0	0	0	0.14	0	0	0	0	0
-0.138	0	0	0.125	0	0	0	0	0	0
-0.182	0	0	0.25	0	0	0	0	0	0
-0.487	0	0	0	0	0	0	0	0	0
-0.033	0	0	0.125	0	0	0	0	0	0
-0.599	0	0	0	0.14	0.09	0	0	0.049	0
-0.102	0	0.093	0.125	0	0.45	0.068	0	0.049	0.091
-0.303	0	0	0.25	0	0	0	0.288	0	0
-0.217	0	0	0	0	0	0	0.096	0	0

-0.245	0	0	0	0	0	0	0	0	0
-0.002	0	0	0	0	0	0	0	0	0
-0.085	0	0	0	0	0	0	0	0	0
-0.026	0	0	0	0	0	0	0	0	0
-0.414	0	0	0	0	0	0	0	0	0
-0.166	0	0	0	0.14	0.09	0	0.096	0	0
-0.041	0	0	0	0	0.09	0	0	0	0
-0.165	0	0	0	0	0	0	0.096	0	0
-0.423	0	0	0	0	0	0	0	0	0
-0.103	0	0	0	0	0.09	0	0	0.098	0
-0.17	0	0	0	0	0	0	0	0	0
-0.713	0	0	0.25	0.14	0.27	0	0.192	0.294	0
-0.288	0	0.093	0.125	0	0	0	0	0	0
-0.177	0	0	0	0	0.09	0	0.288	0	0
-0.038	0	0	0	0.14	0.09	0	0	0	0
-0.431	0	0	0.625	0.28	0.36	0	0.096	0.098	0
-0.173	0	0.186	0.25	0.14	0.54	0	0	0	0
-0.128	0	0	0	0	0.09	0	0	0.049	0
-0.214	0	0	0	0	0.09	0	0	0.049	0
-0.087	0	0	0.25	0	0.09	0	0	0	0
-0.074	0	0	0	0.42	0	0	0	0	0
-0.121	0	0.093	0.125	0.14	0.09	0	0	0	0
-0.794	0	0	0	0	0	0	0	0	0
-0.333	0	0	0	0.14	0	0	0	0	0.091
-0.163	0	0	0	0	0.18	0.068	0	0	0
-0.053	0	0	0	0	0	0	0	0	0
-0.456	0	0	0	0	0	0	0	0	0
-0.12	0	0	0.25	0	0	0	0	0	0
-0.184	0	0	0	0	0	0	0	0	0
-0.107	0	0	0	0	0	0	0	0	0
-0.469	0	0	0.125	0	0	0	0	0	0
-0.223	0	0	0	0	0.27	0	0.096	0.049	0
-0.187	0	0	0	0	0.09	0.068	0	0	0
-0.198	0	0.093	0.375	0.14	0	0	0.096	0	0
-0.008	0	0	0	0	0	0	0	0	0
-0.039	0	0	0	0	0	0	0.096	0	0
-0.664	0	0	0	0	0.18	0	0	0	0
-0.017	0	0.093	0	0	0	0	0	0	0
-0.057	0	0	0	0	0	0	0	0	0
-0.346	0	0	0	0	0.18	0	0	0	0
-0.375	0	0.093	0	0	0	0	0	0	0
-0.389	0	0	0	0	0	0	0	0	0
-0.075	0	0	0	0	0	0	0	0	0
-0.347	0	0	0	0	0	0	0	0	0
-0.071	0	0	0	0	0	0	0	0	0
-0.021	0	0	0	0	0	0	0.096	0	0
-0.467	0	0	0.125	0	0	0	0	0	0
-0.369	0	0	0	0	0	0	0	0	0
-0.058	0	0	0	0	0	0	0	0	0
-0.071	0	0	0	0	0	0.136	0	0	0

-0.097	0	0	0	0	0	0	0.192	0	0
-0.092	0	0	0	0	0	0	0	0	0
-0.019	0	0	0.125	0	0	0	0.096	0	0
-0.262	0	0	0	0	0	0	0	0	0
-0.001	0	0	0	0	0.09	0	0	0	0
-0.18	0	0	0	0	0	0	0	0	0
-0.154	0	0	0	0	0.27	0	0	0.098	0
-0.268	0	0	0	0	0	0	0.096	0	0
-0.019	0	0	0.5	0.14	0.27	0.068	0.288	0	0
-0.689	0	0	0	0	0	0	0	0	0
-0.106	0	0.093	0.375	0.28	0.36	0.068	0	0	0
-0.589	0	0	0.125	0	0	0	0	0	0
-0.244	0	0.186	0.125	0	0	0	0	0.049	0.091
-0.203	0	0	0	0	0	0	0	0	0
-0.011	0	0.093	0.125	0.28	0.09	0	0	0	0
-0.143	0	0	0	0	0	0	0	0	0
-0.45	0	0	0	0	0	0	0	0	0
-0.261	0	0	0.125	0	0	0	0.096	0	0
-0.031	0	0	0	0	0	0	0	0	0
-0.052	0	0	0.25	0	0	0	0	0.049	0
-0.181	0	0.093	0.125	0.14	0.18	0.204	0	0	0
-0.207	0	0	0	0	0	0	0	0	0
-0.223	0	0	0.25	0.14	0	0	0	0.049	0
-0.129	0	0	0	0	0	0	0	0	0
-0.319	0	0	0	0	0	0	0	0	0
-0.267	0	0	0	0	0	0	0	0	0
-0.084	0	0	0	0.14	0	0	0	0	0
-0.003	0	0.093	0.5	0.56	0.27	0	0.096	0.098	0
-0.648	0	0	0	0	0	0	0	0	0
-0.12	0	0	0	0	0.36	0	0.096	0	0
-0.003	0.04	0	0.25	0.14	0	0	0	0	0
-0.878	0	0	0	0	0	0	0	0	0
-1.346	0	0	0.375	0	0.09	0	0	0	0
-0.514	0	0	0	0	0	0	0	0	0
-0.03	0	0	0	0	0	0	0	0	0
-0.465	0	0	0	0	0.09	0	0	0	0
-0.241	0	0	0	0	0	0.068	0	0	0
-0.283	0	0	0	0	0	0	0	0	0
-0.1	0	0	0	0	0	0	0	0	0
-0.153	0	0	0	0	0	0	0	0	0
-0.157	0	0	0	0	0.09	0	0	0	0
-0.321	0	0.093	0.125	0	0.27	0	0	0	0
-0.447	0	0	0	0	0.09	0	0	0.049	0
-0.269	0	0	0	0	0	0	0	0	0
-0.424	0	0	0	0	0	0	0	0	0
-0.018	0	0	0	0	0	0	0	0	0
-0.442	0	0.186	0	0.14	0	0.068	0	0	0
-0.069	0	0	0.25	0	0	0	0	0	0
-0.318	0	0.093	0	0.28	0.18	0	0	0	0
-0.025	0	0.093	0.75	0.14	0.72	0	0	0	0

-0.02	0	0	0.25	0	0.09	0	0.096	0	0
-0.13	0	0	0	0	0	0	0	0	0
-0.006	0	0	0.125	0	0.09	0	0	0	0
-0.123	0	0	0.25	0.28	0.09	0.204	0.192	0	0
-0.173	0	0	0.125	0	0	0	0.096	0	0
-0.032	0	0	0	0	0	0	0	0	0
-0.76	0	0	0	0	0	0	0	0	0
-0.278	0	0	0	0	0	0	0	0	0
-0.038	0	0	0.125	0	0	0	0	0	0
-0.55	0	0	0	0	0	0	0	0	0
-0.133	0	0	0.125	0	0.09	0	0	0	0
-0.215	0	0	0	0	0	0	0	0	0
-0.121	0	0	0	0	0	0	0	0	0
-0.148	0	0	0.125	0	0.36	0	0	0	0
-0.202	0	0	0	0	0	0	0.096	0	0
-0.449	0	0	0	0	0	0	0	0	0
-0.002	0	0.093	0	0	0	0.068	0	0	0
-0.115	0	0	0	0	0	0	0	0	0
-0.153	0	0	0	0	0	0	0	0	0
-0.251	0	0.093	0	0	0	0	0	0	0
-0.029	0	0	0	0	0	0	0	0	0
-0.226	0	0	0	0	0	0	0	0	0
-0.26	0	0.093	0.125	0.14	0	0	0	0	0
-0.13	0	0	0	0	0	0	0	0	0
-0.193	0	0	0	0	0	0	0	0	0
-0.146	0	0	0.125	0	0	0	0.192	0	0
-0.25	0	0	0	0	0	0	0	0	0
-0.083	0	0.093	0.375	0.56	0.18	0.068	0	0	0.091
-0.165	0	0.093	0	0	0	0	0	0	0.091
-0.348	0	0	0	0	0	0	0	0	0
-0.331	0	0	0	0.28	0.09	0	0	0	0
-0.377	0	0	0	0	0	0	0	0	0
-0.195	0	0	0	0	0	0	0	0	0
-0.027	0	0	0	0	0	0	0	0.098	0
-2.121	0	0	0	0	0	0	0.096	0	0
-0.196	0	0.186	0	0.14	0.09	0	0	0	0
-0.137	0	0	0	0	0	0	0	0	0
-0.344	0.04	0.186	0	0.28	0.09	0	0.192	0	0
-0.011	0	0	0	0	0	0	0	0	0
-0.364	0	0	0	0	0	0	0	0	0
-0.138	0	0	0.125	0	0	0	0	0	0
-0.228	0	0	0.125	0	0.09	0	0.096	0	0
-0.069	0	0	0.25	0	0.09	0	0.096	0	0
-0.022	0	0	0	0	0	0	0	0	0
-0.082	0	0.093	0	0	0	0.068	0	0	0
-0.174	0	0	0.125	0	0	0	0	0	0
-0.055	0	0.093	0	0	0	0.204	0.192	0	0
-0.524	0	0	0	0.14	0.18	0	0	0.098	0
-0.104	0	0.186	0.875	0.14	0.81	0	0.192	0	0
-0.015	0	0	0	0	0	0	0	0	0

-0.214	0	0	0	0	0	0	0	0	0
-0.199	0	0.186	0.125	0.14	0	0	0.096	0.196	0
-0.058	0	0	0	0	0	0	0	0	0
-0.351	0	0.093	0.125	0	0.81	0.136	0	0	0
-0.041	0	0	0	0	0	0	0	0	0
-0.164	0	0	0	0	0	0	0	0	0
-0.388	0	0	0.125	0	0	0	0	0	0
-0.073	0	0	0.125	0.14	0.18	0	0	0	0
-1.071	0	0	0	0	0	0	0	0	0
-0.092	0	0	0	0	0	0	0	0	0
-0.181	0	0.186	0.625	0.84	0.09	0	0.096	0	0
-0.019	0	0.093	0.125	0.42	0.09	0.136	0	0	0
-0.191	0	0	0.125	0	0	0	0	0	0
-0.002	0	0.093	0	0	0.36	0	0	0	0
-0.066	0	0	0	0	0.09	0	0	0	0
-1.044	0	0.093	0.125	0	0	0	0	0	0
-0.276	0.04	0	0.125	0.14	0.09	0	0	0	0
-0.037	0	0	0.25	0.14	0.36	0	0.096	0	0.091
-0.078	0	0	0	0	0	0	0	0	0
-0.122	0	0	0	0	0	0	0	0	0
-0.134	0	0	0.125	0	0	0	0	0	0
-0.032	0	0	0	0	0	0	0	0	0
-0.086	0	0	0	0	0	0	0	0	0
-0.553	0	0	0	0	0.09	0	0	0	0
-0.276	0	0.093	0	0	0.09	0	0.096	0	0
-0.086	0	0	0	0.14	0.18	0	0	0	0
-0.102	0	0	0	0	0.09	0	0	0	0
-0.016	0	0	0	0.14	0.18	0	0	0.098	0
-0.578	0	0.093	0.125	0	0	0	0	0	0
-0.255	0	0.093	0	0.14	0	0	0	0	0
-0.78	0	0	0	0	0	0	0	0	0
-0.869	0	0.093	0.5	0	0.45	0	0	0	0
-0.432	0	0	0.125	0	0	0	0	0.049	0
-0.529	0	0	0	0	0	0	0	0.049	0
-0.012	0.04	0.558	0.75	0.42	0.36	0.272	0	0	0
-0.32	0	0.093	0	0	0.09	0	0	0	0
-0.495	0	0	0	0	0	0	0	0	0
-0.504	0	0	0.125	0	0.36	0	0	0	0
-0.068	0	0	0.125	0.28	0.09	0.136	0.096	0	0
-0.286	0	0	0	0	0	0	0	0	0
-0.487	0	0	0.125	0	0.09	0	0	0.049	0
-0.032	0	0.093	0.75	0.28	0.18	0	0	0.049	0
-0.747	0	0	0	0	0.09	0	0.288	0	0
-0.045	0	0	0.125	0	0.09	0	0	0	0
-0.176	0	0	0.375	0.14	0.18	0	0	0	0
-0.354	0	0.186	0.25	0.42	0.09	0	0	0	0
-0.163	0	0	0	0	0	0	0	0	0
-0.266	0	0.093	0.125	0.14	0.18	0	0	0.049	0
-0.396	0	0	0	0	0	0	0	0.147	0
-0.352	0	0	0.125	0	0	0	0	0	0

-0.039	0	0	0	0	0	0	0	0	0
-0.221	0	0	0	0	0	0	0	0	0
-0.347	0	0	0	0	0	0	0	0	0
-0.245	0.04	0	0	0	0.09	0.136	0	0	0
-0.499	0	0	0	0	0	0	0	0.049	0
-0.233	0	0	0	0	0	0	0	0	0
-0.034	0	0	0.125	0.14	0.09	0	0	0	0
-0.027	0	0	0	0	0	0	0	0	0
-0.03	0	0	0	0	0	0	0	0	0
-0.719	0	0	0	0.14	0	0	0	0	0
-0.594	0	0.093	0	0	0	0	0	0	0
-0.664	0	0	0	0	0	0	0	0	0
-0.457	0	0.093	0.125	0	0	0	0	0	0
-0.991	0	0.279	0.25	0.28	0.18	0	0	0	0
-0.087	0	0	0	0	0.18	0	0	0.098	0
-0.752	0	0	0	0	0	0	0	0	0
-0.214	0	0	0	0	0	0	0	0	0
-0.204	0	0.186	0	0	0	0.204	0	0	0
-0.347	0	0	0	0	0.45	0.136	0.288	0.343	0
-0.019	0	0	0	0	0	0	0.096	0	0
-0.847	0	0	0.125	0	0	0	0.096	0	0
-0.233	0	0	0.25	0	0	0	0	0.098	0
-0.038	0	0	1.125	0.28	0.18	0	0	0	0
-0.008	0	0	0	0	0	0	0	0	0
-0.078	0	0	0	0	0	0	0	0	0
-0.013	0	0	0	0	0	0	0	0	0
-0.107	0	0	0	0.14	0	0	0	0	0
-1.949	0	0	0	0	0	0	0	0	0
-0.125	0	0	0	0.14	0.09	0	0.096	0	0
-0.308	0	0.093	0.375	0	0.09	0.068	0.096	0.098	0
-0.255	0	0	0	0.14	0	0	0	0	0
-0.003	0	0	0	0	0	0	0	0	0.091
-0.043	0	0	0.125	0	0.27	0	0	0	0
-0.115	0	0.186	0.875	0.42	0.09	0	0.096	0.098	0
-0.272	0	0	0	0.14	0	0	0	0	0
-0.1	0	0	0	0	0	0	0	0	0
-0.395	0	0	0.125	0.14	0	0	0	0	0
-0.273	0	0	0	0	0	0	0	0	0
-0.145	0	0	0	0	0	0	0	0	0
-0.579	0	0.093	0	0	0	0	0	0	0
-0.258	0	0	0.25	0	0	0	0	0	0
-0.426	0	0	0.125	0	0	0	0	0	0
-0.046	0	0	0	0.14	0	0	0	0.196	0
-0.003	0	0.186	0.25	0.14	0	0.272	0	0	0
-0.345	0	0	0.125	0	0	0	0	0	0
-0.498	0	0	0	0.14	0	0	0	0	0
-0.768	0	0	0	0.14	0	0	0	0	0
-0.544	0	0	0	0	0	0	0	0.049	0
-0.384	0	0	0	0	0	0	0	0	0
-0.174	0	0	0	0	0	0	0	0	0

-0.004	0	0.093	0.5	0	0.09	0	0	0	0
-0.382	0	0	0	0	0	0	0	0	0
-0.18	0	0	0	0	0	0	0	0	0
-0.025	0	0	0.5	0	0	0	0	0	0
-0.022	0	0	0	0	0.18	0.068	0.096	0	0
-0.161	0	0	0.125	0	0	0	0	0	0
-0.029	0	0	0	0	0	0	0	0	0
-0.395	0	0	0	0.14	0.09	0	0	0.098	0
-0.01	0	0	0	0	0	0	0	0	0
-0.076	0	0	0.125	0	0	0	0	0	0
-0.539	0	0.093	0.125	0.28	0.09	0	0	0	0
-0.027	0	0	0	0	0	0	0	0	0
-0.386	0	0	0	0	0.09	0	0	0	0
-0.175	0	0.186	0.375	0.42	0	0	0.096	0	0
-0.023	0	0.186	2.25	0.7	0.45	0.068	0.096	0	0
-0.968	0	0	0.125	0	0	0	0	0	0
-0.398	0	0	0	0	0	0	0	0	0
-0.08	0	0	0.125	0	0	0	0	0	0
-0.038	0	0	0	0	0	0	0	0	0
-0.35	0	0	0	0	0	0	0	0	0
-0.396	0	0.093	0	0	0	0	0	0	0
-0.063	0	0	0.125	0	0.54	0.136	0	0	0
-0.194	0	0.186	0	0.28	0	0	0.096	0.098	0
-0.473	0	0	0.25	0	0	0	0	0	0
-0.157	0	0	0	0	0.18	0	0	0	0
-0.088	0	0.093	0.125	0	0	0	0	0	0
-0.404	0	0	0	0	0	0	0	0.049	0
-0.182	0	0	0	0.14	0.09	0.068	0	0	0
-0.052	0	0.093	0.5	0	0	0	0	0	0
-0.342	0	0	0.25	0.14	0.09	0	0.096	0	0
-0.309	0	0.093	0.5	0.28	0	0	0	0	0
-0.501	0	0	0	0	0	0	0	0	0
-0.113	0	0.093	0.125	0	0	0	0	0	0
-0.052	0	0	0	0	0	0	0	0	0
-0.162	0	0	0.375	0	0.45	0	0	0	0
-0.05	0	0	0	0	0	0	0	0	0
-0.308	0	0.093	0	0.14	0	0	0	0	0
-0.03	0	0	0	0	0	0	0	0	0
-0.827	0	0	0	0	0	0	0	0	0
-0.043	0	0	0	0	0	0	0	0.098	0
-0.274	0	0	0	0	0	0	0	0	0
-0.224	0	0	0	0.14	0	0	0	0.049	0
-0.724	0	0.093	0.125	0.14	0	0	0	0	0
-0.042	0	0	0	0	0	0	0.096	0	0
-0.138	0.08	0.186	1	0.7	0.54	0.068	0	0	0
-0.017	0	0	0	0	0	0	0	0.049	0
-0.045	0	0	0.125	0	0	0.136	0	0	0
-0.13	0	0	0	0	0	0	0	0	0
-0.563	0	0	0.125	0.14	0	0.136	0	0	0
-0.501	0	0.093	0	0.14	0.18	0	0	0.049	0

-0.195	0	0	0.75	0	0.09	0	0	0	0
-0.149	0	0	0	0	0	0	0.096	0	0
-0.068	0	0.093	0.375	0	0.18	0.068	0	0.049	0
-0.221	0	0	0.25	0	0	0	0	0	0
-0.115	0	0	0	0	0.27	0.068	0	0.049	0
-0.193	0	0	0	0	0	0	0	0	0
-0.024	0	0	0	0	0	0	0	0.049	0
-0.075	0	0	0	0	0.18	0	0.288	0	0
-0.319	0	0.186	0	0	0	0.204	0	0	0
-0.386	0	0	0	0	0	0	0	0.098	0
-0.576	0	0	0.375	0.14	0.09	0	0	0	0
-0.544	0	0.186	0.375	0.14	0.36	0.136	0	0	0
-0.071	0	0.093	0	0.14	0.09	0	0.096	0.049	0
-0.058	0	0	0.25	0	0.27	0	0	0	0
-0.265	0	0	0	0	0	0	0	0	0
-0.152	0	0	0	0	0	0	0	0	0
-0.515	0	0	0	0	0.09	0.068	0	0	0
-0.19	0	0	0	0.14	0.18	0	0	0	0
-2.571	0	0	0	0	0	0	0	0	0
-0.441	0	0	0	0	0	0	0	0	0
-0.5	0	0	0	0	0	0	0	0	0
-0.025	0	0	0	0	0	0	0	0	0
-0.159	0	0	0	0	0	0	0	0	0
-0.275	0	0.093	0.125	0	0.27	0	0	0	0
-0.019	0	0	0	0.14	0	0	0	0	0
-0.135	0	0	0.125	0	0	0	0	0	0
-0.001	0	0	0	0	0	0	0	0	0
-0.104	0	0	0	0	0	0	0	0	0
-0.182	0	0	0	0	0	0	0	0	0
-0.083	0	0.093	0	0.28	0.09	0	0	0	0
-0.21	0	0	0.125	0	0	0	0	0	0
-0.239	0	0	0	0	0	0	0.096	0	0
-0.061	0	0	0	0	0	0	0	0	0
-0.118	0	0	0	0	0	0	0	0	0
-0.154	0	0.186	0	0	0.09	0	0.096	0	0
-0.103	0	0	0	0	0	0	0	0	0
-0.116	0	0	0	0	0	0	0	0.049	0
-0.097	0	0	0	0	0	0	0	0	0
-0.024	0	0	0	0.14	0.18	0	0	0	0
-0.147	0	0.186	0.375	0.14	0	0	0	0	0
-0.192	0	0	0	0	0	0	0	0	0
-0.017	0	0	0	0	0	0	0	0	0
-0.178	0	0.093	0	0	0	0	0.096	0	0
-0.172	0	0	0	0	0	0	0	0	0
-0.362	0	0	0	0	0	0	0	0	0
-0.085	0	0	0.125	0	0	0	0	0	0
-0.05	0	0	0	0	0	0	0	0	0
-0.222	0	0	0	0	0	0	0	0	0
-0.576	0	0	0	0	0	0	0	0	0
-0.175	0	0	0	0	0.09	0.272	0	0	0

-0.08	0	0	0	0	0	0	0	0	0
-0.025	0	0	0.25	0	0.27	0	0.192	0	0
-0.17	0	0	0	0	0	0	0	0	0
-0.136	0	0	0	0	0	0	0	0.049	0
-0.441	0	0	0.125	0	0	0	0	0	0
-0.173	0	0	0	0	0	0	0	0	0
-0.106	0	0	0	0	0	0	0	0	0
-0.496	0.04	0	0	0	0	0	0	0	0
-0.118	0	0	0	0	0	0	0.192	0	0
-0.043	0	0	0	0	0	0	0	0	0
-0.166	0	0.186	0.125	0	0	0	0.096	0	0
-0.142	0	0	0	0	0	0	0	0	0
-0.227	0	0	0	0	0	0	0	0	0
-0.175	0	0	0	0	0.09	0	0	0	0
-0.075	0	0	0	0	0	0	0	0	0
-0.027	0	0	0.125	0	0.18	0	0	0.049	0
-0.081	0	0	0	0	0	0	0	0	0
-0.3	0	0	0	0	0	0	0	0	0
-0.009	0	0.465	0.25	0.42	0.45	0.068	0	0	0.091
-0.125	0	0.093	0.25	0.28	0	0	0.192	0	0
-0.037	0	0.465	0.5	0.42	0.81	0	0.096	0	0
-0.484	0	0	0	0	0	0	0	0.049	0
-0.188	0	0.093	0	0	0	0	0	0	0
-0.133	0	0	0.125	0.14	0	0	0	0	0
-0.005	0	0	0	0	0	0	0	0	0
-0.06	0	0	0	0	0	0	0	0	0
-0.471	0	0	0.125	0	0.18	0.136	0.096	0	0.091
-0.174	0	0	0	0	0.09	0	0	0	0
-0.097	0	0	0	0	0	0	0	0	0
-0.105	0	0	0	0	0.09	0	0	0	0
-0.281	0	0	0	0	0	0	0	0.049	0
-0.415	0	0	0	0	0	0	0	0	0
-0.055	0	0	0	0	0	0	0	0	0
-0.09	0	0	0	0	0	0	0	0	0
-0.209	0	0	0	0	0	0	0	0	0
-0.023	0	0	0	0	0	0	0	0	0
-0.315	0	0	0.125	0	0.09	0	0	0.098	0
-0.182	0	0.093	0.375	0.28	0.45	0	0	0.049	0
-0.414	0	0	0	0	0.09	0	0.096	0	0
-0.038	0	0	0	0	0	0	0	0.049	0
-0.041	0.04	0	0	0	0	0	0	0.049	0
-0.439	0	0.093	0.25	0.28	0.09	0	0.096	0	0
-0.053	0	0	0	0	0	0	0	0	0
-0.298	0	0	0	0	0	0	0	0	0
-0.036	0	0.093	0	0	0	0	0	0	0
-0.335	0	0.093	0	0.28	0.27	0	0	0	0
-0.28	0	0	0	0	0	0	0	0	0
-0.429	0	0	0	0.14	0.27	0.068	0	0.147	0
-0.242	0	0	0	0	0	0	0	0	0
-0.019	0	0	0	0	0	0	0.096	0	0

-0.502	0	0	0	0	0	0	0	0	0
-0.382	0	0	0	0	0	0	0.096	0	0
-0.243	0	0	0	0	0	0	0	0	0
-0.351	0	0	0.125	0	0.09	0	0	0	0
-0.223	0	0	0.25	0	0.09	0	0	0	0
-0.499	0	0.372	0.625	0.28	0.18	0	0	0	0
-0.199	0	0.651	1.75	1.26	0.45	0.068	0	0	0
-0.096	0	0	0.25	0.42	0	0	0	0	0
-0.004	0	0.186	0.5	0	0	0	0	0	0
-0.21	0	0	0	0	0	0	0	0	0
-0.452	0	0.093	0.25	0	0	0.068	0	0	0
-0.064	0	0.093	0	0	0	0	0	0	0
-0.262	0	0	0	0	0	0	0	0	0
-0.324	0	0.093	0.25	0	0	0	0	0	0
-0.285	0	0	0.25	0	0	0	0.288	0	0
-0.099	0	0	0	0	0	0	0	0	0
-0.161	0	0	0	0	0	0	0	0	0
-0.12	0	0	0	0	0	0	0	0	0
-0.075	0	0	0.125	0	0	0	0	0	0
-0.394	0	0.093	0.375	0.14	0.09	0	0	0	0
-0.002	0	0	0	0	0	0	0	0	0
-0.485	0	0	0	0	0	0	0	0	0
-0.249	0	0	0.125	0	0.18	0	0.096	0	0
-0.54	0	0	0	0	0	0	0	0	0
-0.254	0	0	0.125	0	0	0	0	0	0
-0.143	0	0	0	0	0.09	0.068	0	0	0
-0.349	0	0.093	0.125	0.14	0.09	0.204	0	0	0
-0.185	0	0	0.25	0.14	0	0	0	0	0
-0.266	0	0	0	0	0	0	0	0	0
-0.059	0	0.093	0	0.14	0	0	0	0	0
-0.166	0	0	0.125	0.14	0	0	0	0	0
-0.189	0	0.093	0.25	0.14	0	0	0	0.049	0
-0.187	0	0	0	0	0	0	0	0	0
-0.276	0	0	0	0	0	0	0	0	0
-0.475	0	0	0	0	0	0	0	0	0
-0.836	0	0.186	0.625	0.28	0.81	0	0	0	0
-0.209	0	0	0.125	0	0	0	0	0	0
-0.146	0	0	0	0	0.09	0	0	0	0
-0.206	0	0.093	0.125	0	0	0	0	0	0.182
-0.23	0	0	0	0	0	0	0.096	0.098	0
-0.234	0	0	0	0	0	0	0	0.049	0
-0.018	0	0	0	0	0	0.136	0	0	0
-0.153	0	0.093	0.125	0	0.09	0	0	0	0
-0.251	0	0	0	0	0	0	0	0	0
-0.919	0	0	0.125	0	0.09	0	0	0	0
-0.068	0	0	0	0	0	0	0.096	0	0
-0.108	0	0.093	0.375	0	0.36	0	0	0	0
-0.073	0	0.093	0.25	0.14	0	0	0.192	0.049	0
-0.017	0	0	0	0	0.09	0	0	0	0
-0.009	0	0.093	0	0	0	0	0	0	0

-0.269	0	0	0	0	0	0	0	0	0
-0.005	0	0	0.125	0	0	0.068	0	0	0
-0.726	0	0.186	0.25	0	0.27	0	0.096	0	0
-0.08	0	0	0.25	0	0	0	0	0	0
-0.116	0	0	0	0	0.45	0	0	0	0
-0.866	0	0	0	0	0	0	0.096	0	0
-0.214	0	0	0	0	0	0	0	0	0
-0.298	0	0	0.25	0.14	0.09	0	0	0	0
-0.28	0	0	0	0	0.54	0	0	0	0
-0.009	0	0	0	0	0	0	0	0	0
-0.312	0	0	0	0	0	0	0.096	0	0
-0.001	0	0	0.125	0	0.18	0	0	0	0
-0.059	0	0.093	0	0	0	0	0	0	0
-0.357	0	0	0	0	0	0	0	0	0
-0.008	0.04	0	0	0	0.27	0.136	0	0	0
-0.065	0	0	0.125	0.14	0	0	0	0	0
-0.212	0	0	0	0	0	0	0	0	0
-0.289	0.08	0.093	0.375	0	0	0	0.096	0	0
-0.221	0	0	0.125	0	0.09	0	0.288	0	0
-0.157	0	0	0	0	0.45	0	0	0	0
-0.38	0	0	0	0	0	0	0.096	0	0
-0.365	0	0	0	0	0	0	0	0	0
-0.268	0	0	0	0	0	0	0	0	0
-0.003	0	0	0	0	0	0	0	0	0
-0.129	0	0	0	0	0.09	0	0	0	0
-0.821	0	0.093	0.25	0.28	0	0.068	0.096	0	0
-0.135	0	0	0.125	0	0	0	0	0	0
-0.22	0	0	0	0.14	0	0	0	0	0
-0.102	0	0	0	0	0	0	0	0	0
-0.146	0	0	0.125	0.14	0.09	0	0	0	0
-0.241	0	0	0	0	0	0	0.096	0	0
-0.13	0	0	0	0	0	0	0	0	0
-0.167	0	0	0	0	0	0	0	0	0
-0.423	0	0	0	0	0	0	0	0	0
-0.059	0	0	0	0.14	0.09	0	0	0	0
-0.176	0	0	0	0.14	0.36	0.136	0	0	0
-0.175	0	0	0	0	0	0.068	0.096	0.098	0
-0.403	0	0	0	0	0	0	0	0	0
-0.284	0	0	0	0	0	0	0	0	0
-0.268	0	0.186	0	0	0.54	0.204	0	0.049	0
-0.328	0	0	0	0	0	0	0	0	0
-0.413	0	0	0	0	0	0	0	0	0
-0.108	0	0	0	0	0	0	0	0	0
-0.358	0	0	0	0	0	0	0	0	0
-0.147	0	0.093	0	0	0.72	0	0.096	0.049	0
-0.169	0	0	0	0	0	0	0	0	0
-0.332	0	0	0	0	0	0	0.096	0.049	0
-0.019	0	0	0.125	0	0.09	0	0	0	0
-0.222	0	0	0	0	0	0	0	0	0
-0.033	0	0	0.125	0	0	0	0	0	0

-0.771	0	0	0.125	0.14	0	0	0.096	0.098	0
-0.007	0	0.093	0.125	0.14	0	0	0.192	0.196	0
-0.065	0	0	0.125	0	0.36	0	0	0	0
-0.373	0	0.093	0.25	0	0	0.068	0	0	0
-0.104	0	0	0	0	0.09	0	0	0	0
-0.166	0	0.093	0.125	0.14	0.27	0	0	0	0
-0.024	0	0	0.375	0	0	0	0	0	0
-0.102	0	0	0	0	0	0	0	0	0
-0.082	0	0	0.5	0.14	0	0.068	0	0	0
-0.066	0	0	0	0	0	0	0	0	0
-0.515	0	0	0	0	0	0	0	0	0
-0.061	0	0	0	0	0	0	0	0	0
-0.191	0	0	0	0	0	0	0	0	0
-0.06	0	0	0	0	0.27	0	0	0	0
-0.255	0	0	0.125	0.56	0.09	0	0.096	0.049	0
-0.094	0.04	0.186	0.5	0.14	0.18	0.136	0	0	0
-0.082	0	0	0	0	0	0	0	0	0
-1.214	0	0	0.125	0.14	0	0	0	0	0
-0.084	0	0.093	0	0.14	0.09	0	0	0	0
-0.145	0	0.186	0.875	0	0	0	0	0	0
-0.035	0	0	0.125	0	0.18	0	0	0	0
-0.751	0	0	0.25	0	0.36	0	0	0	0
-0.099	0	0.093	0	0.28	0	0	0	0	0
-0.676	0	0.093	0	0.14	0	0	0	0.049	0
-0.016	0	0	0	0	0	0	0	0	0
-0.32	0	0	0	0	0	0	0	0.196	0
-0.096	0	0.093	0	0	0.18	0	0.096	0	0
-0.196	0	0	0	0	0	0	0	0	0
-0.155	0	0	0	0	0.09	0	0.096	0	0
-0.161	0	0	0.125	0.28	0	0	0.192	0	0
-0.086	0	0	0	0	0	0	0	0.049	0
-0.073	0	0	0	0	0	0	0	0	0
-0.191	0	0	0	0	0	0	0	0	0
-0.277	0	0.186	0.375	0.7	0.09	0	0	0	0
-0.604	0	0	0	0	0	0	0	0	0
-0.482	0	0	0	0	0	0	0	0	0
-0.037	0	0	0	0	0	0	0	0	0
-0.172	0	0	0	0	0	0	0	0	0
-0.454	0	0	0.125	0	0.27	0	0	0	0
-0.137	0	0	0	0	0	0	0	0	0
-1.235	0	0.093	0.125	0.28	0	0	0	0.049	0
-0.302	0	0	0	0	0	0	0	0	0
-0.233	0	0	0.125	0.28	0	0	0	0	0
-0.148	0	0	0	0	0	0	0	0	0
-0.258	0	0	0	0	0	0	0	0	0
-0.31	0	0	0	0	0.36	0	0	0	0
-0.28	0	0.093	0.125	0	0	0	0	0	0
-0.081	0	0	0.125	0	0	0	0	0	0
-0.12	0	0	0	0	0	0	0	0	0
-0.152	0	0	0	0	0	0	0	0	0

-0.309	0	0	0	0	0	0	0	0	0
-0.102	0	0	0	0	0.09	0	0	0	0
-0.127	0	0	0.125	0	0	0	0.096	0	0
-0.359	0	0.372	0.375	0	0	0	0.096	0	0
-0.233	0	0	0	0.14	0	0	0.192	0	0
-1.822	0	0	0	0	0	0	0	0	0
-0.153	0	0	0	0	0	0	0	0	0
-0.841	0	0	0	0	0	0	0	0	0
-0.179	0	0.093	0	0.14	0	0	0.096	0	0
-0.016	0	0	0	0	0	0.136	0	0.098	0
-1.152	0	0	0	0.14	0.27	0	0.096	0	0
-0.594	0	0	0	0	0	0	0	0.049	0
-0.38	0	0	0	0	0	0	0	0	0
-0.012	0	0	0	0	0	0	0	0	0
-0.135	0	0.093	0.125	0	0.18	0	0	0	0
-0.288	0	0	0	0	0	0	0	0.049	0
-0.807	0	0	0	0	0	0	0	0	0
-0.107	0	0.186	0.375	0	0	0	0	0	0
-0.119	0	0.093	0.125	0.14	0.27	0	0	0	0
-0.106	0	0	0	0	0	0	0	0	0
-0.596	0	0	0	0	0.09	0	0	0	0
-0.254	0	0	0	0	0	0	0	0	0
-0.095	0	0	0	0	0	0	0	0	0
-0.141	0	0	0	0.14	0	0	0	0	0
-0.487	0	0	0	0	0	0	0	0	0
-0.178	0	0	0	0	0	0	0	0	0
-0.02	0	0	0	0	0.09	0	0	0	0
-0.609	0	0	0.125	0	0.09	0	0	0	0
-0.982	0	0	0.125	0.14	0.09	0	0.096	0	0
-0.467	0	0	0.25	0.14	0.09	0	0	0	0
-0.259	0	0	0	0	0	0	0	0	0
-0.121	0	0	0.25	0	0	0	0	0	0
-0.075	0	0.093	0	0	0	0	0	0	0
-0.056	0	0	0	0	0	0	0	0	0
-0.239	0	0	0	0	0	0	0	0	0
-0.91	0	0	0	0	0	0	0	0	0
-0.291	0	0	0.125	0	0.18	0	0	0.049	0
-0.155	0	0	0	0	0	0	0	0	0
-0.101	0	0.093	0	0	0	0	0	0	0
-0.028	0	0	0	0	0	0	0	0	0
-0.415	0	0	0.125	0	0	0	0	0	0
-0.183	0	0	0	0	0	0	0	0	0
-0.119	0	0	0.125	0	0.09	0	0.096	0	0
-0.011	0	0	0	0	0	0.068	0	0	0
-0.063	0	0	0	0	0	0	0	0	0
-0.466	0	0	0	0	0	0	0	0	0
-0.008	0	0.093	0	0	0.36	0.068	0	0	0
-0.086	0	0.093	0	0	0	0	0	0	0
-0.119	0	0	0	0	0	0	0.096	0	0
-0.143	0	0	0	0	0	0	0.096	0	0

-0.371	0	0	0	0	0	0	0	0.196	0
-0.013	0	0	0	0	0	0	0.096	0	0
-0.005	0	0.093	0	0.28	0	0	0.096	0.049	0
-0.32	0	0	0	0	0	0	0	0	0
-0.102	0	0	0	0	0	0	0	0	0
-1.116	0	0	0.125	0.14	0	0	0.096	0	0.091
-0.48	0	0	0	0	0.27	0	0	0	0
-0.02	0	0	0	0	0	0	0	0	0
-0.105	0	0	0.125	0.14	0.36	0.136	0	0.049	0
-0.215	0	0.093	0.375	0	0.18	0	0	0	0
-0.226	0	0	0	0	0	0	0	0	0
-0.08	0	0.093	0.25	0	0	0	0	0	0
-0.071	0	0.465	0.5	0.7	0.36	0	0.096	0	0
-0.212	0	0	0	0	0	0	0	0.098	0
-0.718	0	0.279	0.75	0.14	0	0	0	0	0
-0.505	0	0.186	0.25	0	0.09	0.068	0.096	0	0
-0.134	0	0	0.125	0	0	0	0	0	0
-0.116	0	0	0.125	0	0	0.068	0	0	0
-0.146	0	0	0	0	0	0	0	0	0
-0.056	0	0	0	0	0	0	0	0	0
-0.534	0	0	0	0	0.18	0	0	0	0
-0.521	0	0	0.375	0.14	0.18	0	0.096	0	0
-0.293	0	0	0.25	0	0	0	0	0	0
-0.173	0	0	0.125	0	0	0	0	0	0
-0.289	0	0	0	0	0.09	0	0	0	0
-0.623	0	0	0	0	0	0	0.096	0	0
-0.15	0	0	0	0	0	0	0	0	0
-0.082	0	0	0.375	0.14	0	0	0	0	0
-0.012	0	0	0	0	0	0	0	0	0
-0.17	0	0	0	0	0	0	0	0	0
-0.014	0	0.093	0.25	0	0.54	0	0.192	0.147	0
-0.319	0.04	0	0	0	0	0	0	0	0
-1.242	0	0.186	0.25	0.14	0	0	0	0	0
-0.145	0	0	0	0	0	0	0	0	0
-0.192	0	0	0	0	0	0	0	0	0
-0.428	0	0.279	0.5	0.28	0.18	0.068	0	0	0
-0.003	0	0	0	0	0	0	0	0.049	0
-0.356	0	0	0.125	0	0	0	0.096	0	0
-0.012	0	0	0	0	0.09	0	0	0.049	0
-0.481	0	0	0.125	0	0.09	0	0	0	0
-0.381	0	0	0	0	0	0	0	0	0
-0.255	0	0.279	0.125	0	0	0	0	0	0
-0.069	0	0	0	0	0	0	0	0	0
-0.563	0	0	0	0.14	0	0	0	0	0
-0.097	0	0	0	0	0	0	0	0	0
-0.046	0	0	0	0	0	0	0	0.098	0
-0.307	0	0.093	0.25	0.7	0.09	0	0.096	0	0
-0.384	0	0	0.125	0	0.09	0	0	0.049	0
-0.04	0	0.093	0	0	0.27	0	0	0	0
-0.269	0	0	0	0	0	0	0	0	0

-0.146	0	0.093	0.375	0	0	0	0	0	0
-0.255	0	0	0.125	0.28	0	0	0	0	0
-0.109	0	0	0	0	0	0	0	0	0.091
-0.613	0	0	0	0	0	0	0	0	0
-0.175	0	0	0.125	0	0	0	0	0	0
-0.47	0	0	0	0	0	0	0	0	0
-0.083	0	0.093	0	0	0.09	0.068	0	0	0
-0.171	0	0.279	1	0.14	0.36	0	0.192	0	0.091
-0.019	0	0	0	0	0.18	0.068	0	0	0
-0.011	0	0	0	0	0	0	0	0	0.091
-0.052	0	0	0	0	0	0	0	0	0
-2.392	0	0	0.25	0	0.18	0	0	0	0
-0.038	0	0.093	0.625	0.56	0.63	0	0	0	0
-0.123	0	0	0	0	0	0	0	0	0
-0.083	0	0	0	0	0	0	0	0	0
-0.16	0	0	0	0	0.09	0	0.096	0	0
-0.121	0	0	0	0	0	0	0	0	0.091
-0.246	0	0	0.125	0	0.09	0	0	0.049	0
-0.344	0	0	0	0	0	0	0	0	0
-0.487	0	0.093	0.125	0	0.36	0	0	0	0
-0.074	0	0	0	0	0	0	0	0	0
-0.257	0	0.093	0.375	0.28	0.09	0	0	0	0
-0.336	0	0	0	0	0	0	0	0	0
-0.087	0	0	0	0	0	0	0	0	0
-0.02	0	0.093	0.375	0	0.09	0.204	0	0.049	0
-0.004	0	0	0	0	0	0	0	0	0.091
-0.01	0	0	0	0	0	0	0	0	0
-0.017	0	0.093	0	0	0	0	0.096	0	0
-0.128	0	0	0.125	0	0	0	0	0	0
-0.25	0	0.372	0.25	0.7	0	0	0	0	0
-0.25	0	0.093	0.5	0	0	0.068	0	0	0
-0.433	0	0	0	0	0	0	0	0	0
-0.18	0	0	0	0	0.09	0	0	0.049	0

Supplementary table S3b. Performance comparison of the ANN regression model with SMO regression and MLPR regression after 10-fold cross-validation

**Regression model**	**Cross-validation**	**Root mean squared error (RMSE)**
ANN regression	10-fold	0.29
SMO regression	10-fold	0.3
MLPR regression	10-fold	0.294

Supplementary table S3c. Feature set (9 types of miRNA binding sites) for 1376 miRNA-target pairs from Baek dataset used for testing miRepress model

Pfold	site1	site2	site3	site4	site5	site6	site7	site8	site9
-0.798	0	0	0.125	0	0	0	0	0	0

-0.48	0	0	0	0.14	0	0	0	0	0
-0.018	0	0	0	0.14	0	0	0.096	0	0
-0.365	0	0	0	0	0	0	0	0	0
-0.071	0	0.093	0	0	0	0	0	0	0
-0.164	0	0	0	0	0	0	0	0	0
-0.311	0	0	0	0	0	0	0	0	0
-0.183	0	0.186	0.125	0.14	0.09	0.136	0	0.049	0
-0.533	0	0	0	0	0	0	0.096	0	0
-0.195	0	0.093	0.125	0.28	0.27	0	0	0	0
-0.28	0	0	0.125	0.28	0.18	0	0	0	0
-0.154	0	0	0	0	0	0	0.096	0	0
-0.246	0	0	0	0	0	0	0	0	0
-0.005	0	0	0.125	0	0	0	0	0	0
-1.08	0	0	0.375	0.28	0.09	0	0.192	0	0
-0.394	0	0.093	0.125	0	0	0	0	0	0
-0.052	0	0	0	0	0	0	0.192	0	0
-0.221	0	0.186	0.375	0.14	0.45	0.068	0.192	0	0
-0.059	0	0.093	0.125	0.14	0	0	0	0	0
-0.131	0	0	0	0	0	0	0	0	0
-0.43	0	0.093	0.375	0.14	0.18	0	0	0	0
-0.044	0	0.186	0.125	0.42	0	0	0.096	0	0
-0.011	0	0.093	0.125	0	0	0	0	0.049	0
-0.614	0	0	0.125	0	0.54	0	0	0	0
-0.617	0	0	0	0	0.18	0	0	0	0
-0.186	0	0	0	0	0	0	0	0	0
-0.296	0	0.093	0	0	0	0	0	0.049	0
-0.328	0	0.093	0.25	0.14	0	0	0	0	0
-0.113	0	0	0	0	0	0	0	0	0.091
-0.034	0	0	0	0	0	0	0	0	0
-0.004	0	0	0	0	0	0	0	0	0
-0.137	0	0.093	0.25	0	0.27	0	0.192	0	0
-0.135	0	0.093	0.125	0.14	0	0	0.096	0	0
-0.444	0	0	0	0.28	0.18	0	0.192	0	0
-0.202	0	0.186	0.125	0.56	0.36	0.136	0	0	0
-0.299	0	0.372	0.375	0.14	0.27	0.068	0	0.049	0
-0.113	0	0.093	0.125	0.28	0	0	0	0	0
-0.199	0	0	0	0	0	0	0	0	0
-0.171	0	0	0	0	0	0	0	0	0
-0.48	0	0	0	0	0	0	0.096	0	0
-0.152	0	0.093	0.375	0.28	0	0	0	0	0
-0.029	0	0.093	0.125	0	0	0	0	0	0.091
-0.103	0	0.186	0.25	0.14	0	0	0	0	0.182
-0.529	0.04	0.093	0.625	0.56	0.18	0	0	0	0
-0.074	0	0	0	0	0	0	0	0	0
-0.008	0.12	0.186	0	0	0	0	0	0.049	0
-0.005	0	0.558	1.125	0.42	0.18	0.136	0.096	0.098	0
-0.109	0	0	0.125	0	0.27	0	0	0	0
-0.033	0	0.186	0.25	0	0.18	0	0	0	0
-0.088	0	0	0.25	0.28	0.09	0.068	0	0	0
-0.025	0	0	0	0.14	0	0	0	0	0

-0.105	0	0	0.25	0	0	0	0	0	0
-0.21	0	0	0	0	0	0	0	0	0
-0.126	0	0.093	0.25	0.56	0.18	0.068	0.096	0	0
-0.001	0	0	0	0	0	0	0	0	0
-0.63	0	0.093	0.25	0	0	0	0.096	0.098	0
-0.431	0	0	0.25	0	0	0.204	0	0	0
-0.923	0	0.186	0.5	0.42	0	0	0	0.049	0
-0.013	0.04	0.186	0.125	0.84	0.45	0	0	0	0
-0.204	0	0	0	0.14	0	0	0	0	0
-0.199	0	0	0	0	0	0	0	0	0
-0.011	0	0.186	0.5	0.14	0.18	0	0.576	0.147	0
-0.355	0	0.093	0.125	0	0	0	0.096	0	0
-0.055	0	0.093	0.625	0.14	0.09	0	0	0.049	0
-0.208	0	0	0.125	0.28	0.18	0	0.096	0	0
-0.219	0	0.093	0.125	0.28	0.09	0	0	0	0
-0.173	0	0	0	0	0	0	0	0	0
-0.008	0	0	0	0	0	0	0	0	0
-0.263	0	0	0	0	0.09	0	0	0	0
-0.244	0	0	0.125	0	0	0	0	0	0
-0.116	0	0.093	0	0	0.18	0	0	0	0
-0.025	0	0.093	0	0	0	0	0	0	0
-0.192	0	0	0.125	0	0	0	0	0	0
-0.121	0	0	0	0	0	0	0	0	0
-0.223	0	0.186	0.375	0.7	0	0	0.096	0	0.091
-0.275	0	0.093	0.375	0.14	0	0	0	0	0
-0.424	0	0	0	0	0.09	0	0	0	0
-0.746	0	0.093	0.125	0	0.18	0.068	0	0	0
-0.316	0	0.372	0.625	0.42	0.09	0	0	0	0
-0.569	0	0	0	0	0	0	0	0	0
-0.141	0	0.093	0	0	0	0	0	0	0
-0.598	0	0	0	0.14	0	0	0.096	0	0
-0.04	0	0	0	0	0	0	0	0	0
-0.535	0	0.093	0	0.42	0	0	0.096	0	0
-0.014	0	0.186	0.125	0.14	0.63	0	0	0	0
-0.24	0	0.651	0.75	0.28	0.54	0.068	0	0.049	0
-0.179	0	0	0.125	0.28	0	0.068	0	0	0
-0.176	0	0.372	0.125	0	0	0	0	0	0
-0.088	0	0	0.125	0	0	0	0	0	0
-0.331	0	0	0.25	0.14	0	0	0.096	0	0
-0.136	0	0.186	0.625	0.14	0.18	0	0	0	0
-0.014	0	0	0	0	0	0	0	0	0
-0.182	0.04	0	0	0.28	0.36	0	0.192	0	0.091
-0.074	0	0.186	0.125	0	0	0	0.096	0	0
-0.124	0	0	0.125	0.7	0.09	0	0	0	0
-0.166	0	0.093	0	0	0	0	0.096	0	0
-0.262	0	0.186	1	0.14	0.09	0	0	0	0
-0.247	0	0	0	0	0	0	0.096	0	0
-0.373	0	0.093	0.25	0	0	0	0	0.245	0
-0.076	0	0.186	0	0.14	0	0	0	0	0
-0.17	0	0	0.125	0.28	0.09	0	0	0	0

-0.068	0	0.093	0	0	0	0	0.096	0	0
-0.219	0	0	0	0	0	0	0	0.049	0.091
-0.082	0	0.093	0.125	0.14	0	0	0	0	0
-0.211	0	0	0.125	0	0	0	0	0	0
-0.006	0	0	0.75	0.14	0.09	0.136	0.096	0	0
-0.125	0	0.186	0.75	0.42	0	0	0	0	0
-0.175	0.04	0.186	0.375	0.14	0.27	0.204	0	0	0
-0.073	0	0	0	0	0	0	0	0	0
-0.046	0	0.093	0	0	0	0	0	0	0
-0.04	0	0	0.125	0.14	0	0	0.096	0	0
-0.019	0	0	0.125	0	0	0.136	0	0	0
-0.048	0.04	0.093	0.125	0.28	0.36	0	0	0.098	0
-0.114	0	0	0	0	0	0	0	0	0
-0.104	0	0.186	0.375	0.14	1.08	0	0	0	0
-0.128	0	0	0.125	0.28	0	0.068	0	0	0
-0.053	0	0	0	0.14	0	0	0	0	0
-0.66	0	0	0	0.14	0	0	0	0	0
-0.568	0	0.093	0.25	0.28	0	0	0.096	0	0
-0.112	0	0	0.125	0	0.36	0	0	0.098	0
-0.16	0	0	0.125	0.42	0	0	0.096	0.049	0
-1.479	0	0	0	0	0	0	0	0	0
-0.015	0	0	0	0	0.27	0	0.096	0	0
-0.041	0	0.279	0.75	0.42	0.09	0.068	0	0.049	0
-0.079	0	0	0	0.14	0	0	0	0	0
-0.088	0	0	0.375	0.14	0	0	0.096	0	0
-0.187	0	0.372	1.375	0.56	0.45	0.068	0	0	0
-0.149	0	0	0	0	0	0	0	0	0
-0.094	0	0	0	0	0	0	0	0	0
-0.132	0	0	0	0	0	0	0	0	0
-0.265	0	0	0	0.14	0.09	0	0	0	0
-0.115	0	0.093	0.375	0.28	0	0	0	0	0
-0.89	0.04	0.465	0.375	0.84	0.09	0.136	0	0	0
-0.041	0.04	0.279	0	0.28	0.09	0	0	0	0
-0.186	0	0	0.25	0	0	0	0	0	0.091
-0.043	0	0.186	0.375	0.14	0	0	0	0	0
-0.235	0	0	0	0.14	0.09	0	0.192	0	0
-0.369	0	0	0	0	0	0	0	0	0
-0.053	0	0.93	1.875	1.82	0.81	0.068	0	0	0
-0.084	0	0	0	0	0	0	0	0	0
-0.367	0	0	0	0	0	0	0	0	0
-0.069	0	0	0.125	0	0	0	0.096	0.049	0
-0.083	0	0	0	0	0.09	0	0.096	0.049	0
-0.062	0	0	0.125	0	0	0	0	0	0
-0.023	0	0	0	0.14	0	0	0	0	0
-0.105	0	0	0.125	0	0.09	0	0.096	0	0
-0.217	0	0.372	0.625	0.56	0.36	0.136	0.096	0	0
-0.196	0.04	0.186	0.25	0.42	0.18	0	0.096	0	0
-0.056	0	0	0	0	0	0	0	0	0
-0.271	0	0	0.125	0	0.18	0	0	0	0
-0.036	0	0.093	0	0	0.09	0	0.192	0	0

-0.495	0	0	1	0.14	0.36	0	0	0.049	0
-0.207	0	0.186	0.5	0.84	0.36	0	0.384	0.049	0
-0.34	0	0	0	0	0	0	0	0	0
-0.015	0	0.186	0.25	0.42	0	0.068	0.096	0.049	0
-0.183	0	0	0	0	0	0	0	0	0
-0.002	0	0	0	0	0	0	0	0	0
-0.146	0	0.093	0.125	0	0	0	0	0.098	0
-0.454	0.04	0	0.125	0	0	0	0	0	0
-0.006	0	0	0	0.14	0	0	0	0	0
-0.007	0	0.093	0	0	0	0	0	0	0
-0.436	0	0	0	0	0	0	0	0	0
-0.087	0.04	0.093	0.25	0	0.09	0	0	0	0
-0.298	0	0	0.125	0	0	0	0	0	0
-0.067	0.04	0.279	0.875	0.56	0.09	0	0	0.049	0
-0.089	0	0	0	0.14	0.18	0	0	0	0
-0.266	0	0	0	0	0	0	0	0	0
-0.141	0	0	0.125	0	0	0	0	0	0
-0.411	0	0	0	0	0	0	0	0	0
-0.113	0	0.093	0.875	0.28	0	0	0	0	0
-0.074	0	0.093	0.25	0	0	0	0.096	0	0
-0.259	0	0.372	0.25	0.42	0.27	0.204	0	0	0
-0.166	0	0	0.125	0	0.09	0	0	0	0
-0.059	0	0	0	0	0.36	0	0	0	0
-0.333	0	0.279	0.25	0.28	0.09	0	0	0	0
-0.323	0.12	0.186	0.625	0	0	0	0	0.147	0
-0.061	0	0	0	0.14	0	0	0	0	0
-0.21	0	0	0.125	0	0	0	0	0	0
-0.273	0	0	0.125	0	0	0	0	0	0
-0.073	0	0	0	0	0	0	0	0	0
-0.004	0	0	0	0	0.09	0	0	0	0
-0.025	0	0.093	0	0.28	0.18	0	0	0.049	0
-0.098	0	0	0	0	0	0	0	0	0
-0.009	0	0	0	0	0	0	0	0	0
-0.634	0	0.093	0.125	0	0.45	0	0	0	0
-0.73	0	0	0	0	0	0	0.096	0	0
-0.441	0	0	0	0	0	0	0	0	0
-0.011	0.04	0.186	0.375	0	0.18	0.068	0	0	0
-0.122	0	0.186	0.25	0	0	0	0	0	0
-0.219	0	0.093	0	0.14	0	0	0	0	0
-0.046	0	0	0	0	0	0	0	0	0
-0.088	0	0.186	0.375	0	0.27	0	0	0	0
-0.958	0	0.093	0.625	0.28	0	0.068	0.192	0	0
-0.016	0	0	0.375	0.56	0.09	0	0	0	0
-0.202	0	0	0.125	0.28	0.09	0	0.192	0	0
-0.084	0	0	0	0	0	0	0	0	0
-0.03	0	0.093	0.25	0	0	0	0.096	0	0
-0.108	0	0	0	0	0	0	0	0	0
-0.612	0	0.093	0.125	0	0.09	0	0	0	0
-0.05	0.04	0.186	0.875	0.42	0.18	0	0.384	0.049	0
-0.338	0	0	0	0	0	0	0	0	0

-0.057	0	0	0	0	0	0	0	0	0
-0.205	0.04	0	0	0.14	0	0	0	0	0
-0.878	0	0	0.25	0	0	0	0	0	0
-0.336	0	0	0	0.42	0.45	0.068	0.096	0.049	0
-0.319	0	0	0.125	0	0	0	0	0	0
-0.189	0	0	0	0	0	0	0	0	0
-0.257	0	0	0	0	0	0	0	0	0
-0.233	0	0	0	0	0	0	0	0	0
-0.039	0	0.093	0	0	0	0	0	0	0
-0.1	0	0	0	0.14	0	0	0	0.196	0.091
-0.318	0	0	0	0	0	0	0	0	0
-0.014	0	0	0	0	0	0	0	0	0
-0.129	0	0	0.125	0	0	0	0	0	0
-0.073	0	0	0.125	0.14	0.09	0	0	0	0
-0.189	0	0.186	0.5	0.42	0.45	0	0.096	0	0
-0.339	0	0	0	0.14	0	0	0.096	0	0
-0.643	0	0	0.125	0	0	0.068	0.192	0	0
-0.147	0	0	0	0	0	0	0	0	0
-0.071	0	0	0.125	0	0.09	0	0	0	0
-0.044	0	0.093	0	0.14	0.18	0.068	0	0.049	0
-0.442	0	0.093	1.125	0.28	0.27	0	0	0	0
-0.062	0	0	0	0	0	0	0.096	0	0
-0.129	0	0	0	0	0	0.068	0	0	0
-0.203	0	0	0.125	0	0.09	0	0	0	0
-0.038	0	0	0.125	0	0	0	0	0	0
-0.595	0.04	0.186	0.375	0	0.09	0	0.096	0	0
-0.674	0	0	0	0	0	0	0	0	0
-0.153	0	0	0.5	0.14	0.09	0	0	0	0
-0.05	0	0	0	0	0	0	0	0	0
-0.274	0	0	0.125	0	0	0	0	0	0
-0.064	0	0	0.125	0.14	0	0	0	0	0
-0.051	0	0	0.125	0.28	0.54	0	0	0	0
-0.177	0	0	0.125	0	0	0	0	0	0
-0.044	0	0.279	0.625	0	0	0	0.096	0.098	0.091
-0.068	0	0.279	0.5	0.7	0.18	0.068	0.096	0	0
-0.038	0	0.093	0.5	0	0	0	0.096	0	0
-0.143	0	0.093	0	0.14	0	0	0	0.098	0.091
-0.242	0.04	0.093	0.375	0.56	0.36	0	0.096	0.049	0
-0.08	0	0	0	0.14	0	0	0	0	0
-0.148	0	0.186	0.25	0	0	0	0	0	0
-0.512	0	0	0.125	0	0	0	0.288	0	0
-0.136	0	0	0	0	0.09	0	0	0	0
-0.193	0	0	0	0	0	0	0	0	0
-0.037	0	0	0	0	0.18	0	0	0	0
-0.446	0	0.186	0.625	0.28	0.36	0.136	0.096	0	0
-0.044	0	0	0	0	0	0	0	0	0
-0.141	0	0	0	0.14	0	0	0	0	0
-0.076	0	0.093	0	0	0.18	0	0	0	0
-0.249	0	0	0.125	0	0.18	0.136	0	0	0
-0.526	0	0.186	0.125	0.14	0.09	0	0	0	0

-0.025	0	0.093	0.125	0.14	0.36	0	0	0	0
-0.185	0	0.093	0.5	0	0.18	0	0	0	0
-0.363	0	0.093	0.125	0	0.09	0.068	0	0	0
-0.059	0	0	0	0	0	0	0	0	0.091
-0.254	0	0	0	0	0.18	0	0	0	0
-0.256	0	0	0	0	0	0	0.096	0	0
-0.035	0	0.186	0.375	0.14	0.09	0	0.096	0	0
-0.009	0	0	0	0	0	0	0	0	0
-0.175	0	0	0	0.14	0	0.068	0.096	0	0
-0.368	0	0	0	0	0	0	0.096	0	0
-0.254	0	0	0.25	0	0	0	0	0	0
-0.033	0	0	0.125	0	0	0	0	0	0
-0.101	0	0	0	0	0	0	0	0	0
-0.204	0	0	0	0	0	0	0	0	0
-0.015	0	0.093	0.375	0	0.09	0	0	0	0
-0.265	0	0	0.125	0	0	0	0	0	0
-0.104	0	0	0	0.14	0	0	0	0	0
-0.307	0	0	0.25	0.14	0.27	0	0.096	0	0
-0.384	0.04	0.279	0.875	0.98	0.09	0	0	0.049	0
-0.053	0	0	0	0	0	0	0	0	0
-0.049	0	0	0	0	0	0	0	0	0
-0.365	0	0.279	0.125	0.14	0	0	0	0	0
-0.428	0	0	0	0	0	0	0	0	0
-0.04	0	0	0	0	0	0	0	0	0
-0.054	0	0	0	0	0	0	0	0	0
-0.332	0	0	0	0.28	0	0	0	0	0
-0.504	0	0	0	0	0	0	0	0.049	0
-0.13	0	0	0.125	0.14	0	0	0	0	0
-0.009	0	0.186	0.5	0	0	0	0	0.049	0
-0.1	0	0	0	0	0	0	0	0	0
-0.447	0.04	0.186	0.125	0	0	0	0	0	0
-0.115	0	0	0	0.14	0.45	0.068	0.096	0	0
-0.16	0	0	0	0	0	0	0	0	0
-0.59	0	0	0	0	0	0	0	0	0
-0.497	0	0	0	0	0	0	0	0	0
-0.345	0	0	0.125	0	0	0	0	0	0
-0.152	0	0.093	0	0	0	0	0	0	0
-0.19	0	0	0	0	0	0	0	0	0
-0.049	0.04	0	0.25	0.42	0.18	0	0	0	0.091
-0.001	0	0	0.5	0	0.09	0	0	0	0
-0.324	0	0	0	0	0	0	0	0	0
-0.028	0	0	0	0	0	0	0	0	0
-0.073	0	0.186	0	0	0	0	0.096	0	0
-0.024	0	0.093	0	0	0	0	0	0	0
-0.149	0	0	0	0	0	0	0	0	0
-0.073	0	0.093	0.125	0.42	0	0	0.096	0.098	0
-0.119	0	0	0	0	0	0	0	0	0
-0.302	0	0.186	0.75	0.42	0.18	0.068	0	0	0
-0.028	0	0	0.125	0	0	0	0	0	0
-0.026	0	0	0	0	0	0	0	0	0

-0.301	0	0.186	0.375	0.42	0	0	0.096	0	0
-0.071	0	0	0.25	0.14	0.27	0	0	0	0
-0.191	0	0	0	0	0	0	0	0.049	0
-0.169	0	0	0.375	0	0.09	0	0	0	0
-0.004	0	0	0	0	0	0	0	0	0
-0.273	0	0	0.25	0.28	0.18	0	0	0	0
-0.242	0	0	0	0.14	0	0	0	0	0
-0.268	0	0	0.25	0	0	0	0	0	0
-0.106	0	0	0.125	0	0	0	0	0	0
-0.278	0	0.093	0.25	0	0	0	0	0	0
-0.482	0	0	0	0	0	0	0	0	0
-0.083	0	0	0	0	0	0	0	0	0
-0.194	0	0	0	0	0	0	0	0	0
-1.6	0	0	0	0	0	0	0	0	0
-0.161	0	0	0	0	0	0	0	0	0
-0.232	0	0	0.125	0	0	0	0.096	0	0
-0.712	0	0	0	0	0	0	0.096	0	0
-0.244	0	0	0	0	0	0	0	0	0
-0.697	0	0.093	0.25	0	0.09	0	0	0	0.182
-0.114	0	0	0.125	0	0	0	0	0	0
-1.509	0	0	0.25	0.14	0	0	0	0	0
-0.018	0.04	0.093	0	0	0	0	0	0	0
-0.449	0	0.186	0.125	0	0	0.068	0	0.098	0
-0.053	0	0	0.125	0	0	0	0	0	0
-0.04	0	0	0	0	0.09	0	0.096	0	0
-0.718	0	0.093	0.125	0	0.18	0	0.192	0	0
-0.927	0	0	0	0	0	0	0	0	0
-0.171	0	0	0	0	0	0	0	0	0
-0.438	0	0	0	0	0	0	0	0	0
-1.329	0	0	0	0.14	0	0	0.096	0	0
-0.16	0	0.093	0.125	0	0.18	0	0	0.049	0
-0.019	0	0	0.125	0	0.09	0.068	0	0	0
-0.128	0	0	0	0.14	0.18	0	0	0	0
-0.048	0	0	0	0	0	0	0	0	0
-0.748	0	0	0	0	0	0	0	0	0
-0.085	0	0	0	0	0	0	0	0	0
-0.12	0	0	0	0	0	0	0	0	0
-0.122	0	0	0	0	0	0	0	0	0
-0.066	0	0	0	0	0	0	0	0	0
-0.476	0	0	0	0	0	0	0	0	0
-0.056	0	0	0.375	0	0.09	0.068	0	0	0
-0.569	0	0	0	0	0	0	0	0	0
-0.134	0	0	0	0	0	0	0	0	0
-0.322	0	0	0.125	0	0.27	0	0	0	0
-0.782	0	0	0.25	0.14	0.18	0.068	0	0	0.182
-0.403	0	0	0	0	0	0	0	0.049	0
-0.145	0	0	0	0	0	0	0	0	0
-0.236	0	0	0	0	0	0	0	0	0
-0.438	0	0	0	0	0	0	0	0	0
-0.2	0	0	0.125	0.14	0	0	0	0	0

-1.041	0	0	0.125	0	0.18	0	0	0	0
-0.282	0	0	0	0.14	0	0	0	0	0
-0.133	0	0	0.25	0.14	0	0	0.096	0	0
-0.951	0	0.186	0.125	0	0	0	0	0	0
-0.106	0	0	0	0	0	0	0	0	0
-0.789	0	0	0.25	0	0	0	0	0	0
-0.451	0	0	0	0	0	0	0	0	0
-0.223	0	0	0	0	0	0	0	0	0
-0.203	0	0.093	0.125	0.28	0.09	0.272	0.096	0	0
-0.576	0	0	0	0	0	0	0	0	0
-0.028	0	0	0.125	0	0.09	0	0	0	0
-0.415	0	0	0	0.14	0	0	0	0	0
-0.331	0	0	0	0	0	0	0	0	0
-0.165	0	0	0	0	0	0	0	0	0
-0.735	0	0	0.125	0	0	0	0	0	0
-0.525	0	0.093	0.375	0.14	0	0.136	0	0.049	0
-0.892	0	0	0	0	0.09	0	0	0	0
-0.595	0	0	0	0	0	0.136	0	0	0
-0.23	0	0	0	0	0	0	0	0	0
-0.896	0	0	0.125	0	0	0	0	0	0
-1.135	0	0	0	0	0.09	0	0	0	0
-0.361	0	0.093	0	0.14	0.09	0	0	0	0
-0.045	0	0	0	0	0	0	0	0	0
-0.327	0	0	0	0	0	0	0.096	0	0
-0.127	0	0	0	0	0	0	0	0	0
-0.019	0	0	0.125	0	0.27	0	0	0	0
-0.319	0	0	0	0	0.09	0	0	0	0
-0.264	0	0	0	0	0	0	0	0	0
-0.064	0	0.093	0	0	0	0	0	0	0
-0.379	0	0	0	0	0	0	0	0	0
-0.22	0	0	0	0	0.09	0.068	0	0	0
-0.159	0	0	0.125	0	0	0	0	0.049	0
-0.523	0	0	0	0	0	0	0	0	0
-0.253	0	0	0	0	0	0	0	0	0
-0.115	0	0	0	0	0.18	0	0	0	0.091
-0.255	0	0	0	0	0	0	0	0.049	0
-0.05	0	0	0.5	0	0.63	0.068	0	0	0
-0.03	0	0	0	0	0	0	0	0	0
-0.039	0	0	0	0	0	0	0	0	0
-0.08	0	0.186	0.125	0.14	0.63	0	0	0.049	0
-0.181	0	0	0	0.14	0	0	0	0	0
-0.182	0	0	0	0	0.18	0	0	0	0
-0.387	0	0	0	0	0	0	0	0	0
-0.165	0	0.093	0	0	0.09	0	0	0	0
-0.486	0	0	0	0	0	0	0	0	0
-0.236	0	0	0	0	0	0	0	0	0
-0.214	0	0	0.125	0	0	0.068	0	0.049	0
-0.098	0	0	0.125	0	0.09	0	0	0	0
-0.504	0	0	0	0	0	0	0	0.098	0
-0.368	0	0	0	0	0.27	0.068	0.096	0.049	0

-0.075	0	0	0	0	0	0	0	0	0
-0.187	0	0	0.125	0.14	0.09	0.136	0	0.098	0
-0.238	0	0	0.125	0	0	0	0	0	0
-0.385	0	0	0	0	0.09	0	0	0	0
-0.201	0	0	0	0	0	0	0	0	0
-0.377	0	0	0	0	0	0	0	0	0
-0.155	0	0.093	0	0	0	0	0	0.049	0
-0.071	0	0	0.125	0.14	0.18	0	0.096	0.049	0
-0.19	0	0	0	0.14	0.09	0.068	0	0	0
-0.229	0	0	0	0	0.09	0	0	0	0
-0.456	0	0	0.125	0	0	0	0	0	0
-0.322	0	0	0	0	0.18	0	0	0	0
-0.234	0	0	0	0	0	0	0	0.098	0
-0.061	0	0	0.125	0	0	0	0	0	0
-0.151	0	0	0	0	0	0	0	0	0
-0.257	0	0	0	0	0	0	0	0	0
-0.028	0	0	0	0	0	0	0.096	0	0
-0.077	0	0	0.125	0	0	0.068	0	0.049	0
-0.086	0	0	0	0	0	0	0	0	0
-0.01	0	0	0	0	0	0.068	0.096	0	0
-0.069	0	0	0	0	0.09	0	0.096	0	0
-0.324	0	0	0	0	0	0	0	0	0
-0.737	0	0	0	0	0	0	0	0	0
-0.092	0	0	0	0	0.18	0	0	0.147	0
-0.874	0	0	0	0	0	0	0	0	0
-0.001	0.04	0.093	0	0.14	0.18	0	0	0.294	0
-0.157	0	0.186	0.25	0.14	0.72	0	0	0	0
-0.252	0	0	0.25	0.14	0.27	0	0	0	0
-0.052	0	0	0	0	0	0	0	0.147	0
-0.432	0	0.093	0	0.14	0	0	0	0.049	0
-0.095	0	0	0	0	0.18	0.136	0	0	0
-0.018	0	0	0	0	0	0	0	0	0
-0.101	0	0	0	0	0	0	0	0	0
-0.024	0	0	0	0	0	0	0	0	0
-0.089	0	0	0	0	0	0	0	0	0
-0.601	0	0	0	0	0	0.068	0	0	0
-0.931	0	0	0	0	0	0	0.096	0.245	0
-0.061	0	0.093	0.125	0	0	0	0	0	0
-1.485	0	0.093	0.25	0.56	0.99	0.068	0	0.049	0
-0.057	0	0	0.125	0	0	0	0	0	0
-0.198	0	0	0	0	0	0	0	0.049	0
-0.04	0	0	0	0	0.09	0	0	0.049	0
-0.013	0	0	0	0	0	0	0	0	0
-0.197	0	0	0.125	0	0.36	0	0	0	0
-0.348	0	0	0	0	0.09	0	0	0	0
-0.473	0	0	0	0.14	0	0	0	0	0
-0.109	0	0	0	0	0	0	0	0.049	0
-0.339	0	0	0	0	0	0	0	0	0
-0.406	0	0	0	0	0	0	0	0	0
-0.133	0	0	0	0	0	0	0	0	0

-0.155	0	0	0	0	0	0	0	0.147	0
-0.575	0	0	0	0	0	0	0.096	0	0
-0.625	0	0	0	0	0	0	0	0	0
-0.84	0	0	0	0.28	0	0	0	0.049	0
-0.045	0	0	0	0	0	0	0	0.049	0
-0.05	0	0	0	0	0	0	0	0	0
-0.059	0	0	0	0	0	0	0	0	0
-0.069	0	0.093	0	0.14	0	0	0	0	0
-0.089	0	0	0	0	0	0	0	0	0
-0.517	0	0	0	0	0	0	0	0	0
-1.012	0	0	0	0	0	0	0	0	0
-0.074	0	0	0	0	0	0	0	0	0
-0.341	0	0	0	0	0	0	0	0	0
-0.22	0	0	0	0	0	0	0	0	0
-0.045	0	0	0	0	0.09	0	0	0	0
-0.255	0	0	0	0	0.54	0	0	0	0
-0.295	0	0	0	0	0.09	0	0	0.049	0
-0.095	0	0	0.125	0.14	0.09	0	0	0.049	0
-0.348	0	0	0.125	0	0	0	0	0	0
-0.128	0	0	0	0	0	0	0	0.049	0
-0.31	0	0.093	0.125	0	0	0	0	0	0
-0.045	0	0.186	0.125	0.14	0.18	0	0	0	0
-0.099	0	0.093	0.125	0	0	0	0	0	0
-0.254	0	0	0.25	0	0	0	0	0	0
-0.028	0	0.186	0.125	0.14	0	0	0	0	0
-0.626	0	0.372	1.125	0.42	0.18	0	0	0	0
-0.059	0	0	0	0.28	0.45	0	0	0	0
-0.22	0	0.093	0.75	0	0.09	0	0	0	0
-0.359	0	0	0.25	0	0	0	0	0	0
-0.168	0	0	0.25	0	0	0	0	0	0
-0.178	0	0	0	0	0	0	0	0	0
-0.923	0	0	0	0	0	0	0	0	0
-0.382	0	0.093	0.25	0.28	0	0.068	0	0	0
-0.238	0	0	0	0	0	0	0	0	0
-0.121	0	0	0	0	0	0	0	0.049	0
-0.139	0	0	0.125	0.14	0.27	0	0	0	0
-0.075	0	0	0.125	0.14	0.18	0	0	0	0
-0.051	0	0	0.125	0	0	0	0	0	0
-0.152	0	0.093	0	0	0	0	0	0	0
-0.031	0	0	0	0.14	0	0	0	0	0
-0.173	0	0	0.125	0	0	0	0	0	0.091
-0.586	0	0	0	0	0	0	0	0	0
-0.11	0	0	0	0	0	0	0	0	0
-0.04	0	0	0	0	0.36	0	0	0	0.091
-0.068	0	0	0	0	0	0	0	0	0
-0.206	0	0.093	0.5	0.14	0.18	0	0	0	0
-0.098	0	0	0	0	0	0	0	0	0
-0.124	0	0.093	0	0.14	0	0	0	0	0
-0.131	0.04	0.093	0.125	0.14	0.09	0	0.192	0	0
-0.241	0	0	0	0	0	0	0	0	0.091

-0.375	0	0.372	0.5	0.84	0.54	0	0	0	0
-0.332	0	0	0	0	0	0	0	0	0
-0.068	0	0.093	0.5	0	0	0	0.096	0	0
-0.054	0	0	0	0	0	0	0	0	0
-0.082	0	0	0	0	0	0	0	0	0
-0.032	0	0	0	0.14	0.09	0	0	0	0
-0.26	0	0.186	0.25	0.98	0.09	0	0.288	0	0
-0.004	0	0.372	0.625	0.14	0	0	0.096	0	0
-0.08	0	0	0	0	0	0	0	0	0
-0.386	0.04	0.651	0.75	2.24	0.45	0.136	0.192	0	0.091
-0.579	0	0	0	0.28	0.09	0.068	0	0	0.091
-0.065	0	0	0	0	0	0	0	0	0
-0.182	0	0	0	0	0	0	0	0	0
-0.002	0	0	0	0	0	0	0	0	0
-0.157	0	0	0.25	0.14	0.09	0	0	0	0
-0.576	0	0.093	0	0.28	0.18	0	0	0	0
-0.009	0	0	0	0	0	0	0	0	0
-0.044	0	0	0.375	0	0.09	0.272	0	0.049	0
-0.306	0	0	0.125	0.14	0.27	0	0.096	0	0
-0.147	0	0	0	0	0	0	0	0.049	0
-0.111	0.08	0.186	0.375	0	0.18	0	0	0	0
-0.192	0	0.465	0.375	0.14	0.09	0	0	0	0
-0.213	0	0	0	0	0	0	0	0	0
-0.246	0.04	0.093	0.375	0.42	0	0.136	0	0	0
-0.13	0	0	0	0	0	0	0	0	0
-0.066	0	0	0	0	0	0	0	0	0
-1.185	0	0	0.125	0.14	0.18	0	0	0	0
-0.167	0	0.093	0	0	0.09	0	0	0	0
-0.161	0	0	0	0	0	0	0	0	0
-0.008	0	0	0.125	0.14	0.09	0.068	0	0	0
-0.026	0	0	0	0	0	0	0	0	0
-0.301	0.04	0	0	0.14	0	0	0.096	0	0
-0.326	0	0	0.125	0	0	0	0	0	0
-0.021	0	0	0	0	0	0	0	0	0
-0.163	0	0	0.125	0.14	0	0	0	0	0
-0.027	0	0	0	0	0.09	0	0	0	0
-0.105	0	0.186	0.125	0.14	0	0.068	0	0	0
-0.019	0	0	0.25	0.14	0.09	0	0	0	0
-0.039	0	0	0.125	0	0	0.136	0	0	0
-0.226	0	0	0	0.14	0	0	0	0	0
-0.175	0	0	0.125	0	0.27	0.136	0	0	0
-0.018	0	0	0	0	0	0	0	0	0
-0.011	0	0	0	0	0	0	0	0	0
-0.062	0	0	0	0	0	0	0.096	0	0
-0.37	0	0.372	0.375	0	0.27	0.204	0	0	0
-0.57	0	0	0	0	0	0	0	0	0
-0.031	0	0.279	0.5	0.28	0	0	0.192	0.098	0
-0.373	0	0.186	0	0	0	0.068	0.096	0	0
-0.465	0	0	0.25	0.42	0.18	0	0	0	0
-0.04	0	0	0.375	0.14	0	0	0	0	0

-0.195	0	0.093	0.5	0	0.18	0	0	0.049	0
-0.145	0	0	0.5	0.28	0.09	0	0.096	0	0
-0.05	0	0	0	0	0	0	0	0	0
-0.218	0	0	0	0	0	0	0	0	0
-0.031	0	0	0	0	0.09	0	0	0	0
-0.256	0	0	0.25	0.14	0	0	0	0	0
-0.406	0	0	0.875	0.28	0	0.068	0.288	0	0
-0.066	0	0	0	0	0	0	0	0	0
-0.115	0	0	0.125	0	0	0	0	0	0
-0.422	0	0	0	0	0	0	0	0	0
-0.142	0	0	0.25	0	0	0	0	0	0
-0.125	0	0.093	0	0.14	0	0	0	0	0
-0.212	0	0.093	0.125	0	0	0	0.096	0	0
-0.255	0	0	0	0.14	0	0	0.096	0	0
-0.095	0	0.372	0.625	0.28	0.45	0	0.096	0	0
-0.286	0	0	0	0	0	0	0	0	0
-0.355	0	0	0.125	0	0.09	0	0.096	0	0
-0.289	0	0	0.125	0.14	0.09	0	0.096	0	0
-0.264	0	0	0	0.14	0.09	0	0	0	0
-0.005	0	0.465	0.5	0.98	0.36	0	0	0	0
-0.039	0	0	0.375	0	0.27	0	0	0	0.182
-0.147	0	0.186	1.25	0.42	0.63	0	0	0	0
-0.275	0	0	0.125	0	0	0	0	0	0
-0.254	0	0.186	0.375	0.14	0.54	0.068	0	0	0
-0.102	0	0.093	0	0	0	0	0	0	0
-0.163	0	0	0.375	0	0	0	0	0	0
-0.075	0	0	0	0.14	0.09	0	0.096	0	0.091
-0.014	0	0	0	0	0	0	0	0	0
-0.089	0.04	0	0	0.14	0	0	0	0	0
-0.006	0	0.186	0.25	0.14	0.27	0	0	0	0
-0.182	0	0	0.25	0.14	0	0.068	0	0	0
-0.011	0	0	0	0	0	0	0	0	0
-0.051	0	0	0	0	0	0	0.096	0	0
-0.554	0	0.093	0	0.14	0.09	0	0	0	0
-0.092	0	0.372	0.625	0.98	0.18	0	0	0	0
-0.238	0	0	0	0	0	0	0.096	0.049	0
-0.023	0	0	0.25	0.14	0.09	0	0	0	0
-0.266	0	0	0	0.14	0	0	0	0	0
-0.057	0	0	0.125	0.42	0	0	0	0	0
-0.218	0	0.279	0.75	0.14	0.09	0.068	0	0	0
-0.888	0	0.093	0.25	0	0.45	0	0	0	0
-0.167	0.04	0	0	0	0	0	0	0	0
-0.117	0	0	0.125	0	0	0	0	0	0
-0.228	0.04	0	0.25	0.14	0	0	0	0	0
-0.111	0	0	0	0	0	0	0	0	0
-0.168	0	0	0	0	0	0	0	0	0.091
-0.204	0	0	0	0	0	0	0.096	0	0
-0.024	0	0	0	0	0	0	0	0.098	0
-0.05	0	0	0.125	0	0	0	0	0	0
-0.123	0	0	0	0	0	0	0	0	0

-0.315	0	0	0	0	0	0	0	0	0
-0.063	0	0	0	0	0	0	0	0	0
-0.027	0	0	0.25	0.14	0	0	0.096	0	0
-0.25	0	0	0	0	0	0	0	0	0
-0.097	0	0	0.125	0.14	0	0	0	0	0
-0.407	0	0.093	0.75	0.28	0.09	0	0	0	0
-0.403	0	0	0	0	0	0	0.096	0	0
-0.105	0	0.093	0	0.14	0	0	0	0	0
-0.012	0	0	0	0	0	0	0	0	0
-0.187	0.04	0	0.5	0.56	0	0	0.096	0.049	0
-0.005	0	0	0	0.28	0	0	0	0	0
-0.189	0.04	0	0.25	0	0.18	0	0	0	0
-0.565	0	0.093	0.875	0.7	0.54	0.136	0	0	0
-0.386	0	0	0	0	0.18	0	0	0	0
-0.004	0	0	0	0	0	0	0	0	0
-0.281	0	0.093	0.25	0	0.09	0	0.192	0.098	0.091
-0.119	0	0	0	0	0	0	0	0	0
-0.124	0	0	0.5	0	0.18	0	0	0	0
-0.151	0	0	0.125	0	0	0	0	0	0.091
-0.529	0	0	0	0	0	0	0	0	0
-0.097	0	0	0	0.14	0.09	0.068	0.096	0	0
-0.036	0	0	0	0	0	0	0	0	0
-0.077	0	0	0	0	0	0	0.096	0.098	0
-0.398	0	0.093	1	0.14	0.27	0.136	0.096	0	0
-0.02	0	0	0	0	0	0.068	0	0	0
-0.159	0	0	0	0	0	0	0	0	0
-0.278	0	0.279	0	0.14	0	0	0	0	0
-0.474	0	0.093	0.375	0.42	0.27	0	0.096	0	0
-0.358	0	0	0.125	0	0	0	0	0	0
-0.183	0	0	0.125	0	0	0	0.096	0	0
-0.001	0	0	0	0	0	0	0	0.049	0
-0.013	0	0	0	0	0	0	0	0	0
-0.05	0	0	0	0	0	0	0	0	0
-0.002	0	0	0	0	0	0	0	0	0
-0.051	0	0	0	0	0	0	0	0	0
-0.08	0	0.186	0	0	0	0	0	0	0
-0.409	0	0.093	0	0	0	0	0	0	0
-0.05	0	0	0	0	0	0	0	0	0
-0.352	0	0	0	0	0	0	0	0	0
-0.048	0	0	0.125	0	0	0	0	0	0
-0.143	0	0	0.125	0.14	0	0	0	0	0
-0.058	0	0	0	0	0	0	0	0	0
-0.102	0	0	0.125	0	0	0	0.096	0	0
-0.264	0	0	0.25	0	0	0	0	0	0.091
-0.03	0	0	0	0	0	0	0	0	0
-0.12	0	0	0.25	0	0	0	0	0.049	0
-0.137	0	0	0.125	0	0	0	0	0	0
-0.208	0	0	0	0	0	0	0	0	0
-0.166	0	0	0	0	0	0	0	0	0
-0.174	0	0	0	0	0	0	0	0	0

-0.702	0	0	0	0	0	0	0	0	0
-0.107	0	0	0	0	0	0	0	0	0
-0.236	0	0	0	0	0	0	0	0	0
-0.255	0	0	0	0	0	0	0	0.049	0
-0.158	0	0.093	0	0	0	0	0	0	0
-0.074	0	0.093	0.125	0	0	0.136	0	0	0
-0.18	0	0	0	0	0	0	0	0	0
-0.107	0	0	0	0	0	0	0	0	0
-0.295	0	0	0	0.14	0	0	0.192	0	0
-0.092	0	0	0	0	0	0	0	0	0
-0.355	0	0	0	0	0	0	0	0	0
-0.293	0	0	0	0	0	0	0	0	0.091
-0.083	0	0.093	0.375	0.42	0.18	0	0.096	0	0
-0.161	0	0.093	0.125	0.14	0.09	0	0	0.098	0
-0.234	0	0	0.5	0.14	0	0	0	0	0
-0.062	0	0	0	0	0	0	0	0	0
-0.054	0	0	0	0	0	0	0	0	0
-0.317	0	0.093	0.125	0.28	0.18	0	0	0	0
-0.084	0	0	0.125	0	0	0	0	0	0
-0.087	0	0	0.125	0.28	0	0	0.096	0	0
-0.018	0	0.093	0.25	0.14	0.27	0.068	0.096	0	0
-0.008	0	0	0.125	0	0.27	0	0	0.049	0
-0.577	0	0	0.125	0	0	0	0	0	0
-0.176	0	0.186	0.625	1.26	0.9	0	0	0	0
-0.255	0	0	0	0	0	0	0	0	0
-0.03	0	0.093	0.125	0	0.18	0	0.288	0	0
-0.168	0	0	0	0	0	0	0	0	0
-0.226	0	0	0.125	0	0.09	0	0	0	0
-0.011	0	0	0	0	0	0	0	0	0
-0.253	0	0.093	0.125	0.14	0	0	0	0	0
-0.053	0	0	0	0	0	0	0	0	0
-0.111	0	0.093	0.25	0.14	0	0.068	0	0	0
-0.231	0	0	0	0	0	0	0	0	0
-0.151	0	0	0	0	0	0	0	0	0
-0.055	0	0	0.375	0.28	0.09	0	0	0	0
-0.005	0	0	0	0	0	0	0.096	0	0
-0.03	0	0	0.125	0	0	0	0.096	0	0
-0.072	0	0	0.125	0	0	0	0	0	0
-0.053	0	0	0	0.14	0	0	0	0.098	0
-0.282	0	0	0	0	0	0	0	0	0
-0.077	0	0	0	0	0	0	0	0	0
-0.354	0	0	0	0	0	0	0	0	0
-0.536	0	0	0	0	0	0	0	0	0
-0.224	0	0	0	0	0	0	0	0	0
-0.036	0	0	0	0	0	0	0	0	0
-0.043	0	0	0	0	0	0	0	0	0
-0.388	0	0	0	0	0	0	0.096	0	0
-0.016	0	0	0	0	0	0	0	0	0
-0.132	0	0	0	0	0	0	0	0	0
-0.019	0	0.372	0	0.28	0	0	0	0	0

-0.358	0	0.186	0.25	0.42	0.18	0	0	0	0
-0.845	0	0	0	0	0	0	0	0	0
-0.056	0	0.093	0.25	0	0.18	0	0.096	0	0
-0.791	0	0.093	0.125	0	0	0	0.096	0	0
-0.04	0	0	0	0	0	0	0	0	0
-0.12	0.04	0	0	0	0.09	0	0	0	0
-0.082	0	0.093	0.625	0	0.09	0	0	0	0
-0.093	0	0	0	0	0	0	0	0	0
-0.118	0	0.093	0	0	0	0	0	0	0
-0.622	0	0	0	0	0	0	0	0	0
-0.112	0	0.186	0.375	0.14	0	0	0	0	0
-0.195	0	0	0	0	0	0	0	0	0
-0.023	0.12	0.093	0.25	0.28	0.27	0	0.096	0	0
-0.015	0	0	0	0	0	0	0	0	0
-0.343	0.04	0.186	1.25	0.42	0.27	0.068	0	0	0
-0.265	0	0	0.125	0.28	0.09	0	0	0	0
-0.148	0.08	0.093	0.625	0.56	0	0.068	0	0	0
-0.136	0	0.093	0.5	0.14	0.18	0	0	0	0
-0.001	0	0	0.25	0.56	0.18	0.068	0.096	0	0
-0.647	0	0	0	0	0	0	0	0	0
-0.028	0	0	0	0	0	0	0	0	0
-0.464	0	0	0	0	0	0	0	0	0
-0.518	0	0	0	0	0	0	0	0	0
-0.035	0	0	0.625	0.28	0	0	0	0.098	0
-0.207	0.04	0	0.375	0	0.18	0	0	0	0
-0.417	0	0	0.25	0	0	0	0	0	0
-0.454	0	0	0	0	0	0	0	0	0
-0.098	0	0	0	0	0	0	0	0	0
-1.05	0	0	0.125	0	0.09	0	0	0	0
-0.286	0	0	0	0	0	0	0	0	0
-0.258	0	0	0	0	0	0	0	0	0
-0.244	0	0	0	0	0	0	0	0	0
-0.027	0	0	0.375	0.14	0	0	0	0	0
-0.061	0	0	0	0	0.45	0	0	0	0
-0.186	0	0	0	0	0	0	0	0	0
-0.264	0	0	0.25	0	0.09	0.068	0	0	0
-0.024	0.04	0	0.125	0	0.27	0	0	0	0.091
-0.175	0	0	0	0	0	0	0	0	0
-0.05	0	0	0	0	0	0	0	0	0
-1.199	0	0	0	0	0	0	0	0	0
-0.089	0	0	0	0	0	0	0	0	0
-0.177	0	0	0	0	0	0	0	0	0
-0.028	0	0	0	0	0	0	0	0	0
-0.345	0	0	0.125	0	0	0	0.096	0	0
-0.071	0	0.186	0	0	0.27	0	0	0	0
-0.523	0	0	0	0	0	0	0	0	0
-0.011	0.04	0	0	0	0.09	0.068	0	0	0
-0.016	0	0	0	0	0	0	0	0	0
-0.051	0	0	0	0	0.27	0	0	0	0
-0.195	0.04	0	0	0	0	0	0	0	0

-0.042	0	0.093	0.625	0	0	0	0	0	0
-0.054	0	0	0	0	0	0	0	0	0
-0.059	0	0	0	0	0.18	0	0	0	0
-0.228	0	0	0	0	0	0	0	0	0
-0.032	0	0	0.25	0	0.18	0	0	0	0
-0.286	0	0	0	0	0	0	0	0	0
-0.27	0	0	0	0	0	0	0	0	0
-0.358	0	0	0	0	0	0	0	0	0
-0.091	0	0	0	0.14	0.09	0	0	0	0
-0.196	0	0	0	0	0	0	0	0	0
-2.8	0.04	0.186	1.125	0.28	0.54	0.34	0	0	0
-0.337	0	0	0	0.14	0.27	0	0	0	0
-0.75	0	0	0.125	0	0	0	0	0	0.091
-0.229	0	0	0	0	0	0	0	0	0
-0.201	0	0	0	0	0	0	0	0	0
-0.824	0	0	0.125	0	0.09	0	0.096	0	0
-0.186	0	0	0	0	0	0	0	0	0
-0.306	0	0	0.125	0	0	0	0	0	0
-0.152	0	0	0.25	0	0.09	0	0.096	0	0
-0.128	0	0	0	0	0	0	0	0	0
-0.023	0	0	0	0	0	0	0	0	0
-0.411	0.08	0	0.125	0	0.09	0	0	0	0
-0.2	0	0	0	0	0	0	0	0	0
-1.761	0	0	0	0	0	0	0.096	0	0
-0.554	0	0	0	0	0	0	0	0	0
-0.04	0	0	0.125	0	0.18	0	0	0	0
-0.357	0	0.186	0.375	0	0.09	0	0.096	0	0
-0.269	0	0	0	0	0	0	0	0.049	0
-0.431	0	0	0	0	0	0	0	0	0
-0.04	0	0	0	0	0	0	0	0	0
-0.959	0	0	0	0	0	0	0	0	0
-0.201	0	0	0	0	0	0	0	0	0
-0.371	0	0.093	0.125	0	0.18	0	0	0	0
-0.444	0	0.093	0	0	0	0	0	0	0
-0.352	0	0	0	0	0	0	0	0	0
-0.007	0	0	0	0	0	0	0	0	0
-0.488	0	0	0.125	0	0	0	0	0	0
-0.28	0	0	0	0	0	0	0	0	0
-0.274	0	0	0	0	0	0	0	0	0
-0.156	0	0	0	0	0	0	0	0	0
-0.572	0	0	0	0	0.18	0	0	0	0
-0.438	0	0	0	0	0	0	0	0	0
-0.08	0	0	0	0	0	0	0	0	0
-0.208	0	0	0	0	0	0	0	0	0
-0.204	0	0	0	0	0	0	0	0	0
-0.001	0	0	0	0	0	0	0	0	0
-0.114	0	0	0	0	0	0	0	0	0
-0.264	0	0	0	0	0	0.136	0	0	0
-0.783	0	0	0.5	0	0	0	0	0	0
-0.208	0	0	0	0	0	0	0	0	0

-0.44	0	0	0	0	0	0	0	0	0
-0.166	0	0	0.125	0	0	0	0	0	0.091
-0.233	0	0	0	0	0	0	0	0	0
-1.659	0	0.093	0	0.14	0.09	0.068	0	0	0
-0.544	0	0	0.25	0	0.09	0	0	0	0
-0.024	0	0	0	0	0.09	0	0	0	0
-0.165	0	0.093	0	0.28	0.09	0.068	0	0	0
-0.573	0	0	0	0	0	0	0	0	0
-0.03	0	0	0	0	0	0	0	0	0
-1.024	0	0	0	0	0	0	0	0	0
-0.001	0	0	0.125	0	0	0	0	0	0
-0.013	0	0	0	0	0	0	0	0	0
-0.715	0	0	0	0	0.09	0	0.096	0	0
-0.014	0	0	0.125	0	0	0.136	0	0	0
-0.2	0	0	0	0	0	0	0	0	0
-0.535	0	0	0	0	0	0	0	0	0
-0.615	0	0	0.375	0	0	0.068	0	0	0
-0.129	0	0	0	0	0	0	0	0	0
-0.043	0	0	0.125	0	0	0	0	0	0
-0.338	0	0	0	0	0	0	0	0	0
-0.596	0	0	0	0	0	0	0	0	0
-0.525	0	0	0	0	0	0	0	0	0
-0.008	0	0.093	0	0.14	0.09	0	0	0	0
-0.106	0	0	0.125	0.14	0	0.136	0.096	0	0
-0.648	0	0	0	0	0	0	0.096	0	0
-0.199	0	0	0	0.14	0	0	0.096	0	0
-0.008	0	0.093	0	0	0	0	0	0	0
-0.84	0	0	0	0	0	0	0	0	0
-0.624	0	0.093	0	0	0.09	0	0	0	0
-0.553	0	0	0	0	0.09	0	0	0	0
-0.057	0	0	0	0	0	0	0	0	0
-0.604	0	0	0.125	0	0	0.068	0	0	0
-0.342	0	0	0	0.14	0	0	0	0	0
-0.029	0	0	0	0	0	0	0	0	0
-0.161	0	0	0	0	0	0	0	0	0
-0.17	0	0	0	0	0	0	0	0	0
-0.134	0	0	0	0	0	0	0	0	0
-0.394	0	0	0	0.14	0	0	0	0	0
-0.107	0	0	0	0.14	0.09	0	0	0.049	0
-1.43	0	0	0.125	0	0.09	0	0	0	0
-0.03	0	0	0	0	0.09	0	0	0	0
-0.143	0	0	0	0	0	0	0.096	0.049	0
-0.698	0	0	0	0	0	0	0	0	0
-0.214	0	0.093	0	0	0	0	0	0	0
-0.028	0	0	0	0	0.09	0.068	0.096	0	0
-1.907	0	0.093	0.375	0.28	0.09	0.204	0	0	0
-1.683	0	0	0	0	0.09	0	0.096	0	0
-0.292	0	0	0	0	0	0	0	0	0
-0.165	0	0	0	0	0	0	0	0	0
-1.005	0	0	0	0	0	0	0	0	0

-0.077	0	0	0	0	0	0	0	0	0
-0.321	0	0	0	0	0	0	0	0	0
-1.021	0	0.093	0	0.14	0.18	0.068	0	0	0
-0.175	0.04	0	0	0	0	0	0	0	0
-1.155	0	0.093	0.25	0	0	0.068	0.096	0	0
-0.211	0	0	0	0	0	0	0	0.049	0
-0.157	0	0	0	0	0	0	0	0	0
-0.058	0	0	0	0	0	0	0	0	0
-0.038	0	0	0	0	0	0	0	0	0
-0.254	0	0	0	0	0	0	0	0	0
-0.792	0	0.093	0	0.28	0	0	0	0	0
-0.066	0	0	0	0	0	0	0	0	0
-0.44	0	0	0.125	0.14	0	0.068	0	0	0
-0.04	0	0	0	0	0	0	0	0	0
-0.526	0	0	0.25	0	0.09	0	0	0	0.091
-0.08	0	0	0	0	0	0	0	0	0
-0.238	0	0	0	0	0	0	0	0	0
-0.083	0	0	0	0	0	0	0	0	0
-0.011	0	0	0.125	0.14	0	0	0	0.049	0
-0.263	0	0	0	0	0	0	0	0	0
-0.776	0	0	0	0	0	0	0	0	0
-0.677	0	0	0	0	0	0	0	0	0
-0.022	0	0	0	0	0.09	0	0	0	0
-0.459	0	0.093	0	0	0	0	0	0	0
-0.407	0	0	0	0	0	0	0	0	0
-0.786	0	0	0.125	0.14	0	0	0	0.049	0
-0.714	0	0	0	0	0	0	0	0	0
-0.104	0	0	0	0	0	0	0	0	0
-0.585	0	0	0	0	0	0	0	0	0
-0.231	0	0	0	0	0	0	0.096	0	0
-0.063	0	0	0	0	0	0	0	0	0
-0.117	0	0	0	0	0	0	0	0	0
-0.3	0	0	0	0	0	0	0	0	0
-0.025	0	0	0	0	0	0	0	0	0
-0.549	0	0	0.125	0	0	0	0	0	0
-0.011	0	0	0	0	0	0	0	0	0
-0.244	0	0	0	0	0	0	0	0	0
-0.237	0	0	0.5	0	0.09	0	0.096	0.049	0
-0.266	0	0	0	0	0	0	0	0	0
-1.77	0.04	0.093	0	0	0	0.068	0.096	0	0
-0.112	0	0	0	0	0	0	0	0	0
-0.071	0	0	0	0	0	0	0	0	0
-0.193	0	0	0	0	0.09	0	0	0	0
-0.144	0	0	0.125	0	0	0	0	0	0
-0.481	0	0	0	0	0	0	0	0	0
-0.179	0	0	0.25	0	0	0	0	0	0
-0.06	0	0	0.125	0	0	0	0	0	0
-0.196	0	0	0	0	0	0	0	0	0
-0.034	0	0	0	0	0	0	0	0	0.091
-0.279	0	0	0.375	0	0	0	0.096	0	0

-0.428	0	0	0.25	0.14	0	0	0	0	0
-0.03	0	0	0.375	0	0	0	0	0	0
-0.207	0	0	0.125	0	0	0	0	0	0
-0.074	0	0	0	0	0.09	0.068	0	0	0
-0.083	0	0	0	0	0	0	0	0	0
-0.475	0	0	0	0	0	0	0	0	0
-0.529	0	0	0.25	0	0	0	0	0	0
-0.24	0	0.093	0.125	0	0	0	0	0	0
-0.164	0	0	0	0	0	0	0	0.098	0
-0.043	0	0	0	0	0	0	0	0	0
-0.19	0	0	0	0	0	0	0	0	0
-1.285	0	0	0	0	0	0	0	0	0
-0.143	0	0	0	0	0	0	0	0	0
-0.84	0	0.093	0.125	0	0.09	0	0	0	0
-0.019	0	0	0	0.14	0	0	0	0	0
-0.085	0	0	0	0	0	0	0	0	0
-0.111	0	0	0.125	0	0	0	0	0	0
-0.16	0	0.093	0	0	0	0.068	0	0	0
-0.183	0	0	0	0	0	0	0	0	0
-0.292	0	0	0	0	0.09	0	0	0	0
-0.536	0	0	0	0	0	0	0	0	0
-0.79	0	0	0	0	0	0	0	0	0
-0.383	0	0	0	0	0	0	0	0	0
-0.223	0	0	0	0	0	0	0	0	0
-0.079	0	0	0.125	0	0	0	0	0	0
-0.453	0	0	0.125	0.14	0	0	0	0	0
-0.783	0	0	0	0	0	0	0	0	0
-0.029	0	0	0	0.14	0	0	0	0	0
-0.141	0	0	0	0	0	0	0	0	0
-1.229	0	0	0	0	0	0	0	0	0
-0.642	0.04	0	0	0.14	0.09	0.068	0	0	0
-0.021	0	0	0	0	0	0	0	0	0
-0.332	0	0	0.25	0	0	0.136	0	0	0
-0.338	0	0	0	0.14	0	0	0.096	0	0
-1.222	0	0	0	0	0	0	0	0	0.091
-0.372	0	0	0	0	0	0	0	0	0
-0.743	0	0.093	0.25	0.28	0.09	0.068	0	0	0
-0.267	0	0	0	0	0	0	0	0	0
-0.016	0	0	0	0	0	0	0	0	0
-0.209	0	0	0.125	0	0.27	0.068	0	0	0
-0.019	0	0	0	0	0	0	0	0	0
-0.056	0	0	0.125	0	0	0	0	0	0
-0.195	0	0	0	0	0	0	0	0	0
-0.005	0	0.093	0.125	0.14	0	0	0	0	0
-0.505	0	0	0.375	0	0.09	0	0.096	0.098	0
-0.184	0	0	0	0	0	0	0	0	0
-0.576	0	0	0	0	0	0	0	0	0
-0.44	0.04	0.093	0.375	0	0.09	0	0	0	0
-1.485	0	0	0.125	0	0	0	0	0	0
-0.135	0	0.093	0.875	0.28	0.27	0.136	0	0	0

-0.198	0	0	0.125	0	0	0	0	0	0
-0.17	0	0	0	0	0	0	0	0	0
-0.053	0	0	0	0	0	0	0	0	0
-0.218	0	0	0.125	0	0	0	0.192	0	0
-0.753	0	0	0.375	0	0.09	0	0.096	0	0
-0.672	0	0	0	0	0	0	0	0	0
-0.836	0	0	0	0	0	0	0	0	0
-0.307	0	0	0	0	0	0.136	0	0.049	0
-0.263	0	0	0	0	0.09	0	0.096	0	0
-0.19	0	0	0	0	0	0	0	0	0
-0.237	0	0	0.25	0.14	0.09	0	0	0	0
-0.135	0	0	0.125	0	0.09	0	0.096	0	0
-2.343	0	0	0.125	0	0	0	0	0	0
-0.459	0	0	0	0	0	0	0	0	0
-0.464	0	0	0.125	0.14	0.09	0.068	0	0	0
-0.186	0	0	0	0	0	0	0	0	0
-0.236	0	0	0	0	0	0	0	0	0
-0.239	0	0	0	0	0	0	0	0	0
-0.098	0	0	0	0	0	0	0	0	0
-0.204	0	0	0.125	0	0	0	0	0	0
-0.786	0.04	0	0	0.14	0	0	0	0	0
-0.204	0	0	0.125	0.28	0	0	0	0	0
-0.03	0	0	0	0	0	0	0	0	0
-0.107	0	0.093	0.125	0	0	0	0	0	0
-1.601	0	0	0	0	0.09	0.068	0	0	0
-0.029	0	0	0	0	0	0	0	0	0
-0.214	0	0	0	0	0	0	0	0	0
-0.092	0	0	0	0	0	0	0	0	0
-0.591	0	0	0	0	0	0.068	0	0	0
-0.216	0	0	0	0	0	0	0	0	0
-0.107	0	0	0	0	0	0	0	0	0
-0.366	0	0	0.25	0	0	0	0	0	0
-0.547	0	0	0	0	0	0	0	0	0
-0.134	0	0	0	0	0	0	0	0	0
-0.996	0	0	0	0.14	0.09	0	0	0	0
-0.131	0	0	0	0	0	0	0	0	0
-0.876	0	0	0	0.14	0	0	0	0	0
-0.17	0	0	0	0	0	0	0.096	0	0
-0.239	0	0	0	0	0	0	0	0	0
-0.183	0	0	0	0	0	0	0	0	0
-0.247	0	0	0	0	0	0	0	0	0
-2.135	0.04	0.093	0	0	0	0	0	0	0
-0.184	0	0	0	0	0.09	0	0	0	0
-1.586	0	0	0.125	0	0	0	0	0	0
-0.054	0	0	0.125	0	0	0	0	0	0
-0.155	0	0	0.125	0	0	0	0	0	0
-0.132	0	0	0	0	0	0	0	0	0
-0.101	0	0	0	0.14	0	0	0	0	0
-0.319	0	0	0.125	0	0	0.068	0	0	0
-0.081	0	0	0	0	0	0	0	0	0

-0.262	0	0	0	0	0	0	0	0	0
-0.418	0	0	0.125	0	0.36	0	0	0	0
-0.057	0	0.093	0.125	0	0	0	0	0	0
-0.613	0	0	0	0	0	0	0	0	0
-0.207	0	0	0	0	0	0	0	0	0
-0.423	0	0	0	0	0	0	0	0	0
-0.147	0	0.093	0.125	0	0	0	0	0	0
-0.03	0	0	0	0	0	0	0	0	0
-0.155	0	0	0.125	0	0	0	0	0	0
-0.047	0	0	0	0	0	0	0	0	0
-0.153	0	0	0	0	0	0	0	0	0
-0.068	0	0	0.125	0	0	0	0	0	0
-0.561	0	0.093	0.125	0	0	0	0	0	0
-0.332	0	0	0	0	0	0	0.096	0	0
-0.074	0	0.093	0	0	0	0	0	0	0
-0.001	0	0	0	0	0	0	0	0	0
-0.19	0	0	0	0	0	0	0	0	0
-0.387	0	0	0	0	0.09	0	0	0	0
-0.524	0	0	0.25	0.14	0.27	0.136	0	0	0
-0.082	0	0	0.25	0.14	0.09	0	0.096	0	0
-0.132	0	0	0	0	0	0	0	0	0
-0.203	0	0	0	0	0	0	0	0	0
-0.017	0	0	0.125	0	0	0	0	0	0
-0.171	0	0	0.125	0	0	0	0.096	0	0
-0.147	0	0	0	0	0	0	0	0	0
-0.25	0	0	0	0	0	0	0	0	0
-0.012	0	0	0.375	0.14	0	0	0	0	0
-0.594	0	0	0	0	0	0	0	0	0
-0.089	0	0	0	0	0	0	0	0	0
-0.199	0	0	0.125	0	0	0	0	0	0
-0.318	0	0	0	0	0	0.068	0	0	0
-0.251	0	0	0	0.28	0	0	0	0	0
-0.181	0	0	0	0	0	0	0	0	0
-0.208	0	0	0	0	0	0	0	0	0
-0.271	0	0	0	0	0.27	0	0	0.049	0
-0.008	0	0	0	0	0.27	0	0	0.049	0
-0.479	0	0	0	0	0	0	0	0	0
-0.102	0	0	0	0	0	0	0	0	0
-0.303	0	0	0	0	0	0	0	0	0
-0.068	0	0	0	0	0	0	0.192	0	0
-0.017	0	0	0.125	0.14	0	0	0	0	0
-0.139	0	0	0	0	0	0	0.096	0	0
-0.654	0	0	0.125	0	0.09	0	0	0	0
-0.077	0	0	0	0	0	0	0	0.049	0
-0.2	0	0	0	0	0.09	0	0	0	0
-0.836	0	0.093	0	0	0.27	0	0	0.147	0
-0.017	0	0	0.25	0	0.27	0	0.096	0.049	0
-0.098	0	0.093	0.125	0.14	0.18	0	0.192	0.049	0
-0.103	0	0	0	0	0.18	0	0	0	0
-0.25	0	0	0	0	0	0	0	0	0

-0.004	0	0	0	0	0	0	0	0	0
-0.349	0	0	0	0	0	0	0	0.049	0
-0.314	0	0	0	0	0	0	0	0	0
-0.11	0	0	0	0	0	0	0	0	0
-0.196	0	0	0	0	0	0	0	0	0
-0.089	0	0	0.125	0.14	0.27	0	0	0.049	0
-1.846	0	0	0	0	0	0	0	0	0
-0.121	0	0	0	0	0	0.068	0	0	0
-0.738	0	0	0.375	0	0.27	0	0	0	0
-0.191	0	0.093	0	0.14	0.09	0	0	0	0
-0.048	0	0	0	0	0	0	0	0	0
-0.132	0	0.186	0	0	0.18	0.136	0	0	0
-1.244	0	0	0	0	0.18	0	0	0	0
-0.224	0	0	0	0	0	0	0	0	0
-0.219	0	0	0	0	0.09	0	0.096	0	0
-0.059	0	0	0	0	0.09	0	0.096	0	0
-0.036	0	0	0	0	0	0	0	0	0
-0.206	0	0	0	0	0	0	0	0.049	0
-0.22	0	0	0.125	0	0.27	0	0.096	0	0
-0.815	0	0	0	0	0	0	0	0.049	0
-0.193	0	0	0	0	0	0	0	0	0
-0.213	0	0	0	0	0	0	0	0	0
-0.069	0	0	0	0	0	0	0	0	0
-0.089	0	0	0	0	0.09	0	0	0	0
-0.218	0	0	0.125	0	0	0	0	0.049	0
-0.18	0	0	0	0	0.09	0.204	0.096	0	0
-0.03	0	0	0	0	0	0	0.096	0	0
-0.627	0	0	0	0	0	0	0	0	0
-0.612	0	0	0	0	0	0	0	0.049	0
-0.018	0	0	0	0	0.18	0	0	0.147	0
-0.098	0	0	0	0	0	0	0	0	0
-0.5	0	0	0	0	0	0	0	0	0
-0.402	0	0	0	0	0.09	0	0	0	0
-0.336	0	0	0	0	0	0	0	0	0
-0.223	0	0	0	0	0	0	0	0	0
-0.07	0	0	0	0	0	0	0	0	0
-0.204	0	0.093	0	0	0	0	0	0	0
-0.047	0	0.093	0.125	0.14	0.54	0	0	0	0
-0.116	0	0	0.125	0	0.18	0	0	0.049	0
-0.291	0	0	0	0	0	0	0	0	0
-0.056	0	0	0	0	0.36	0	0	0	0
-0.046	0	0	0	0	0	0	0	0	0
-0.253	0	0	0	0	0.09	0	0	0	0
-0.111	0	0	0	0	0	0	0	0	0
-0.121	0	0	0	0	0	0	0	0	0
-0.13	0	0	0	0	0	0	0	0	0
-0.023	0	0	0	0	0.18	0	0	0	0
-0.037	0	0	0	0	0.09	0	0	0	0
-0.104	0	0	0	0	0	0	0	0	0
-0.089	0	0	0	0	0	0	0	0.098	0

-0.085	0	0	0	0	0	0	0	0	0
-0.462	0	0	0	0	0.18	0	0	0	0
-0.024	0	0.093	0.125	0	0.09	0.068	0	0.049	0
-0.035	0	0	0	0	0	0	0	0	0
-0.148	0	0	0	0	0	0	0	0	0
-0.121	0	0	0.125	0	0	0	0	0	0
-0.05	0	0	0	0	0.09	0	0	0	0
-0.007	0	0	0	0	0	0	0	0	0
-0.347	0	0	0.25	0.42	0.54	0	0	0	0.091
-0.479	0	0	0	0	0	0	0	0	0
-0.224	0	0	0	0	0	0	0	0	0
-0.026	0	0	0	0	0	0.068	0	0	0
-0.37	0	0	0	0	0	0	0	0	0
-0.172	0	0	0.25	0.14	0.09	0.068	0	0.098	0
-0.48	0	0	0	0	0	0	0	0	0
-0.559	0	0	0.25	0	0.18	0.068	0	0.049	0
-0.479	0	0	0	0	0	0	0	0	0
-0.153	0	0.093	0	0	0.09	0	0	0	0.364
-0.131	0	0	0	0	0	0	0	0	0
-0.317	0	0	0	0	0.18	0	0	0.098	0
-0.885	0	0.093	0.125	0	0	0	0	0	0
-0.511	0	0	0	0	0.09	0	0	0	0
-0.072	0	0	0	0	0	0	0	0	0
-0.04	0	0	0.125	0	0	0	0	0	0
-0.866	0	0	0	0	0.09	0	0	0	0
-0.693	0	0	0	0	0	0	0	0	0
-0.85	0	0	0	0	0	0.204	0	0.049	0
-0.823	0	0	0	0	0	0	0	0	0
-0.121	0	0	0	0	0.09	0	0.096	0	0
-0.143	0	0	0	0	0.09	0	0	0	0
-0.159	0	0	0	0	0	0	0	0.049	0
-0.181	0	0.093	0	0	0	0	0	0	0
-0.259	0	0	0	0.14	0.09	0.136	0	0	0
-0.074	0	0	0	0	0	0	0	0	0
-0.088	0	0	0	0	0.09	0	0	0	0
-0.027	0	0	0	0.28	0.81	0	0	0	0
-1.05	0	0	0	0	0	0	0	0	0
-0.003	0	0	0	0	0	0	0	0	0
-0.126	0	0	0	0	0.18	0	0	0	0
-0.257	0	0	0	0	0.09	0	0	0.098	0
-0.154	0	0	0	0	0	0	0	0.049	0
-0.318	0	0	0	0	0.18	0	0	0	0
-0.018	0	0	0	0	0	0	0	0	0
-0.028	0	0	0	0	0	0	0.192	0	0
-0.071	0	0	0	0.14	0	0	0	0	0
-0.033	0	0	0	0	0	0	0	0.098	0
-0.055	0	0	0	0	0	0	0	0	0
-0.012	0	0	0	0	0	0	0	0	0
-0.342	0	0	0.125	0	0.18	0	0	0	0
-0.13	0	0	0	0	0	0	0	0	0

-0.308	0	0	0	0	0	0	0	0	0
-0.382	0	0	0	0.14	0	0	0	0	0
-0.511	0	0.093	0.125	0	0.18	0	0	0	0
-0.105	0	0	0	0	0	0	0	0.049	0
-0.305	0	0	0	0	0.18	0	0.192	0	0
-0.064	0	0	0	0	0	0	0	0.049	0
-0.192	0	0	0	0	0	0	0	0	0
-0.294	0	0	0	0	0	0	0	0	0
-0.079	0	0	0.125	0	0.18	0	0	0	0
-0.199	0	0	0	0	0	0	0	0	0
-0.397	0	0	0.125	0	0	0	0	0	0
-0.442	0	0.093	0.125	0	0.36	0	0	0	0
-0.057	0	0	0	0	0	0	0	0	0
-0.225	0	0	0	0	0.09	0.068	0	0	0
-0.13	0	0	0	0	0	0	0	0	0
-0.154	0	0	0	0	0	0	0	0	0
-0.151	0	0	0	0	0	0	0	0	0
-0.073	0	0	0	0	0	0	0	0	0
-0.378	0	0.186	0	0	0.09	0.272	0	0.196	0
-0.142	0	0.093	0	0.14	0	0.068	0.192	0.196	0
-0.389	0	0	0	0	0	0	0.096	0	0
-0.304	0	0	0	0.14	0	0	0	0	0
-0.185	0	0	0	0	0	0	0	0	0
-0.296	0	0	0	0	0.09	0	0	0	0
-0.213	0	0.093	0	0	0	0.068	0.096	0.049	0
-0.265	0	0	0	0	0.18	0	0	0	0
-0.07	0	0.279	0	0	0	0	0	0	0
-0.789	0	0	0	0	0	0	0	0.147	0
-0.037	0	0	0	0	0.27	0	0	0	0
-0.406	0	0	0	0	0.09	0	0	0	0
-0.09	0	0	0	0	0	0	0	0	0
-0.29	0	0	0	0	0	0	0	0	0
-0.012	0	0	0.125	0	0	0	0.096	0	0
-0.511	0	0	0.125	0	0.09	0.068	0	0	0
-0.502	0	0	0	0	0	0	0	0	0
-0.224	0	0	0.125	0	0	0	0	0	0
-0.141	0	0	0	0	0.09	0.136	0	0	0
-0.187	0	0	0	0	0	0	0	0	0
-0.787	0	0	0	0	0	0	0	0.049	0
-0.517	0	0	0	0	0	0	0	0	0
-0.433	0	0	0	0	0	0	0	0	0
-0.727	0	0	0.125	0	0.09	0	0	0	0
-0.151	0	0	0	0	0	0	0	0	0
-0.263	0	0	0	0	0	0	0	0	0
-0.455	0	0	0	0	0	0	0	0.098	0
-0.323	0	0	0	0	0	0	0	0	0
-0.455	0	0	0	0	0	0	0	0.049	0
-0.054	0	0	0	0.14	0	0	0	0	0
-0.582	0	0.093	0	0	0	0	0	0.098	0
-0.015	0	0	0	0	0	0	0	0	0

-0.03	0	0	0	0	0.27	0	0	0	0
-0.141	0	0	0	0.14	0.18	0	0	0	0
-0.189	0	0	0	0	0	0	0	0	0
-0.618	0	0	0	0	0.09	0	0	0.098	0
-0.488	0	0	0	0	0	0	0	0	0
-0.169	0	0	0.125	0	0.45	0	0	0	0
-0.214	0	0	0.125	0	0	0	0	0	0
-0.057	0	0	0	0	0	0	0	0	0
-0.147	0	0	0	0	0	0	0	0	0
-0.207	0	0	0	0	0	0	0	0	0
-0.152	0	0	0	0	0	0	0	0.098	0
-0.051	0	0	0	0	0	0	0	0	0
-0.062	0	0	0	0	0.09	0	0	0.049	0
-0.082	0	0	0	0	0	0	0	0	0
-0.198	0	0	0	0	0	0	0	0	0
-0.185	0	0	0	0	0	0	0	0	0
-0.243	0	0	0	0	0	0	0	0	0
-0.285	0	0	0	0.14	0.09	0	0	0.098	0
-0.13	0	0	0	0	0.36	0	0	0	0
-0.135	0	0	0.125	0	0	0	0	0.196	0
-0.312	0	0	0	0	0	0	0	0	0
-0.09	0	0.093	0	0	0	0	0	0.098	0
-0.128	0	0	0	0	0	0	0	0	0
-0.032	0	0	0	0	0	0	0.096	0	0
-0.178	0	0	0	0.14	0	0	0	0	0
-0.023	0	0	0	0	0	0	0	0	0
-0.053	0	0	0.125	0	0	0	0	0	0
-0.275	0	0.093	0	0	0.27	0	0	0	0
-0.482	0	0	0.25	0	0.54	0	0	0.147	0
-0.126	0	0	0	0	0	0	0	0	0
-0.034	0	0.093	0.125	0	0	0	0	0	0
-0.118	0	0	0	0	0	0	0	0	0
-0.141	0	0	0	0	0	0	0	0	0
-0.13	0	0	0	0	0	0	0	0	0
-0.1	0	0	0	0	0.09	0	0	0	0
-0.274	0	0	0	0	0.09	0	0	0	0
-0.054	0	0	0	0.14	0	0	0	0	0
-0.108	0	0	0.125	0.28	0.27	0	0.096	0	0
-0.167	0	0	0	0	0.45	0	0	0.049	0
-0.599	0	0	0	0	0.09	0.068	0	0	0
-0.047	0	0	0	0	0	0	0.096	0	0
-0.012	0	0	0	0	0	0	0	0.049	0
-0.289	0	0	0	0	0.36	0.204	0.096	0	0
-0.388	0	0	0	0	0	0	0	0	0
-0.338	0	0	0	0	0	0	0	0	0
-0.131	0	0	0.125	0	0	0	0.288	0.049	0
-0.057	0	0	0	0.14	0	0	0	0	0
-0.013	0	0	0	0	0.18	0	0	0	0
-0.135	0	0	0	0	0	0	0	0.049	0
-0.477	0	0	0	0	0	0	0	0.049	0

-0.108	0	0.186	0	0	0.18	0.136	0	0	0
-0.32	0	0	0	0	0	0	0	0	0
-0.038	0	0	0.125	0	0.18	0	0	0	0
-0.062	0	0	0	0	0	0	0	0.049	0
-0.031	0	0	0	0	0	0	0	0	0
-0.418	0	0	0.125	0	0.18	0	0	0.049	0
-0.018	0	0	0	0	0	0	0	0	0
-0.026	0	0	0	0	0	0	0	0.098	0
-0.178	0	0	0	0	0.18	0.136	0	0	0
-0.427	0	0	0	0.14	0	0	0	0	0
-0.282	0	0	0.125	0	0.45	0	0.192	0	0
-0.283	0	0	0	0	0	0	0	0	0
-0.24	0	0	0	0	0	0	0	0	0
-0.333	0	0	0	0	0	0	0	0	0
-0.163	0	0	0	0.14	0	0	0	0	0
-0.009	0	0	0	0	0.18	0	0.096	0	0
-0.174	0	0	0	0.14	0	0	0.192	0	0
-0.338	0	0	0.125	0.14	0	0	0	0.049	0
-0.055	0	0	0	0	0	0	0	0	0
-0.441	0	0	0	0	0	0	0	0.098	0
-0.646	0	0	0	0	0	0	0	0	0
-0.092	0	0	0	0	0.27	0	0	0.098	0
-0.486	0	0	0	0	0	0	0	0.049	0
-0.164	0	0	0	0	0.09	0	0	0.049	0
-0.273	0	0	0	0	0	0	0	0	0
-0.259	0	0	0	0	0	0	0.096	0	0
-0.042	0	0	0	0	0	0	0	0	0
-0.091	0	0.093	0	0	0	0	0	0	0
-0.349	0	0	0	0	0	0	0	0	0
-0.099	0	0	0	0	0	0	0	0.196	0
-0.022	0	0	0	0	0	0	0	0	0
-0.138	0	0	0	0	0	0	0	0	0
-0.441	0	0	0	0	0	0	0	0	0
-0.397	0	0	0	0.14	0.36	0	0	0.049	0
-0.069	0	0	0	0	0	0	0	0	0
-0.087	0	0	0	0	0	0	0	0.049	0
-0.042	0	0	0	0	0	0	0	0	0
-0.33	0	0	0	0	0	0	0	0	0
-0.031	0	0	0	0	0.09	0	0.096	0	0
-0.119	0	0	0	0	0	0	0	0	0
-0.135	0	0	0	0	0	0	0	0	0
-0.574	0	0	0	0	0	0	0	0	0
-0.005	0	0	0	0	0.18	0	0	0	0
-0.177	0	0	0	0.14	0	0	0	0	0
-0.085	0	0	0	0	0	0	0	0	0
-0.067	0	0.093	0.125	0	0.09	0	0	0.147	0
-0.042	0	0	0	0	0.18	0	0	0	0
-0.222	0	0	0	0	0	0	0	0	0
-0.341	0	0	0.125	0	0.18	0	0	0.098	0
-0.379	0	0	0	0	0	0	0	0.147	0

-0.182	0	0	0	0	0	0	0	0	0
-0.156	0	0	0	0	0	0	0	0	0
-0.308	0	0	0	0	0	0	0	0	0
-0.126	0	0	0.125	0	0	0	0	0	0
-0.067	0	0	0	0	0	0	0	0	0
-0.112	0	0.093	0	0	0	0	0	0	0
-0.179	0	0	0	0	0	0	0	0.098	0
-0.03	0	0	0	0	0.09	0	0	0	0
-0.265	0	0	0	0	0.18	0	0	0.049	0
-0.048	0	0	0	0	0	0	0	0.098	0
-0.069	0	0	0	0	0.18	0	0	0	0
-0.078	0	0	0	0	0	0	0	0	0
-0.128	0	0	0	0	0	0	0	0.098	0
-0.395	0	0	0	0	0.45	0	0	0	0
-0.107	0	0	0	0	0.09	0.068	0	0	0
-0.658	0	0	0	0	0	0	0	0	0
-0.628	0	0	0	0	0	0	0	0	0
-0.17	0	0	0	0	0	0	0	0	0
-0.104	0	0	0	0	0	0	0	0	0
-0.058	0	0	0	0	0	0	0	0	0
-0.397	0	0	0	0	0	0	0	0	0
-0.351	0	0	0	0	0.09	0	0	0	0
-0.035	0	0	0	0	0	0	0	0	0
-0.63	0	0	0	0	0	0	0	0.147	0
-0.021	0	0	0	0	0	0	0	0	0
-0.039	0	0	0	0	0	0	0	0.098	0
-0.497	0	0	0	0	0	0	0	0.147	0
-0.054	0	0	0	0	0	0	0	0.098	0
-0.675	0	0	0	0	0.27	0	0	0.049	0
-0.069	0	0	0	0	0	0	0	0	0
-0.301	0	0	0	0	0	0	0	0	0
-0.06	0	0	0	0	0	0	0	0	0
-0.053	0	0	0	0	0	0	0	0	0
-0.233	0	0	0	0	0.18	0.204	0	0.098	0
-0.052	0	0	0	0	0	0	0	0	0
-0.367	0	0	0	0	0	0	0.096	0.049	0
-0.769	0	0	0	0	0	0	0	0	0
-0.301	0	0	0	0	0.09	0	0	0	0
-0.01	0	0	0.125	0	0.09	0	0	0.049	0
-1.271	0	0	0	0	0	0	0	0	0
-0.17	0	0	0.25	0	0.27	0	0	0	0
-0.072	0	0	0	0.14	0.09	0	0	0	0
-0.253	0	0	0	0	0.09	0	0	0	0
-0.409	0	0	0	0	0.09	0	0	0.049	0
-0.015	0	0	0	0	0	0	0	0	0
-0.167	0	0.093	0	0	0	0	0	0	0
-0.232	0	0	0.125	0	0	0	0	0	0
-1.07	0	0	0	0	0	0	0	0	0
-1.453	0	0	0	0	0	0	0	0	0
-0.564	0	0	0	0	0	0	0	0	0

-0.012	0	0	0	0	0	0	0	0	0
-0.048	0	0	0.125	0	0.18	0	0	0	0
-0.03	0	0	0	0	0	0	0	0	0
-0.071	0	0	0	0	0	0	0	0.196	0
-0.266	0	0	0	0	0	0	0	0	0
-0.058	0	0	0	0	0	0	0	0	0
-0.026	0	0	0	0	0	0	0	0	0
-0.157	0	0	0	0	0.36	0	0	0	0
-0.008	0	0	0.125	0	0.09	0	0	0.049	0
-0.134	0	0	0.125	0	0	0	0	0.049	0
-0.119	0	0.186	0	0	0.99	0	0	0.147	0
-0.244	0	0	0	0	0.09	0	0	0	0
-0.312	0	0	0.25	0	0	0	0	0	0
-0.103	0	0	0.125	0	0.18	0	0	0	0
-0.024	0	0	0	0	0	0	0	0	0
-0.067	0	0	0	0	0	0	0	0	0
-0.515	0	0	0	0	0	0	0	0	0
-0.042	0	0	0	0	0	0	0	0.049	0
-0.117	0	0	0	0	0	0	0	0	0
-0.172	0	0	0	0	0	0	0	0	0
-0.063	0	0	0	0	0	0	0	0	0
-0.411	0	0	0	0	0	0	0.192	0	0
-0.389	0	0.093	0	0.28	0.63	0.204	0	0.049	0
-0.667	0	0	0	0.14	0.09	0	0	0	0
-0.047	0	0	0	0	0	0	0	0	0
Pfold	site1	site2	site3	site4	site5	site6	site7	site8	site9
-0.798	0	0	0.125	0	0	0	0	0	0
-0.48	0	0	0	0.14	0	0	0	0	0

Supplementary table S3d. Feature set (9 types of miRNA binding sites) for 153 miRNA-target pairs from Selbach dataset used for testing miRepress model

Pfold	site1	site2	site3	site4	site5	site6	site7	site8	site9
-1.03	0	0.279	0.625	0.42	0	0	0.192	0	0
-0.8	0	0	0.375	0	0	0	0	0	0
-0.76	0	0.279	0.375	0.28	0	0.068	0.672	0.147	0
-0.76	0	0.186	0.5	0.98	0.36	0	0.096	0	0
-0.75	0	0.093	0.5	0.42	0.09	0	0	0	0
-0.73	0	0.372	0.5	0.28	0	0	0	0.049	0.091
-0.69	0	0	0.25	0.56	0.09	0.068	0.192	0	0
-0.63	0	0.186	0.375	0.28	0.45	0	0.288	0	0
-0.62	0	0	0.25	0.14	0.63	0	0.192	0	0
-0.58	0	0.186	0.25	0	0.18	0	0	0	0
-0.58	0	0.186	1	0.7	0.18	0	0.096	0	0
-0.55	0	0	0	0	0	0	0.096	0	0
-0.54	0	0	0	0.14	0	0	0	0	0
-0.54	0	0	0	0	0	0	0.096	0	0
-0.54	0	0	0.25	0.42	0.36	0.068	0	0.049	0
-0.54	0	0	0.125	0.28	0	0	0.192	0	0

-0.53	0	0	0	0.14	0	0.068	0.096	0	0
-0.53	0	0	0.125	0	0	0	0	0	0
-0.52	0	0	0.25	0.14	0.18	0	0	0	0
-0.52	0	0	0.75	0.28	0.27	0	0.192	0.049	0
-0.52	0	0	0.25	0.28	0.09	0	0.096	0	0
-0.51	0	0	0	0	0	0	0.096	0	0
-0.49	0	0.093	0.375	0	0.81	0	0.096	0.098	0
-0.49	0	0	0.625	0.28	0	0.068	0.192	0	0
-0.49	0	0	0	0	0	0	0	0.049	0
-0.49	0	0	0	0	0	0	0	0	0.091
-0.49	0	0.093	0.625	0.14	0.09	0	0.096	0	0
-0.48	0	0	0.125	0.14	0	0	0.096	0	0
-0.48	0	0	0	0	0	0	0	0	0
-0.47	0	0.093	0.75	0	0	0	0.384	0	0
-0.47	0	0	1.25	0.28	0	0	0.192	0.049	0
-0.47	0	0.093	0.375	0	0.18	0	0.096	0	0
-0.46	0	0.093	0.375	0	0.36	0.136	0.096	0.098	0
-0.46	0	0	0.125	0.42	0.09	0	0.192	0	0.091
-0.46	0	0	0.25	0	0	0	0	0	0
-0.46	0	0.279	1.625	0.42	0.45	0.068	0.096	0	0
-0.45	0	0	0	0	0	0	0	0	0
-0.43	0	0	0	0.42	0	0	0	0	0.091
-0.43	0	0	0.375	0	0.18	0	0.096	0	0
-0.42	0	0	0	0	0	0	0.096	0	0
-0.42	0	0.093	1.25	0.14	0.36	0	0.192	0	0
-0.4	0	0	0.125	0	0.36	0	0	0	0
-0.4	0	0.186	0.875	0.42	0	0	0.288	0	0
-0.4	0	0	0.125	0	0	0	0	0	0
-1.46	0	0	0	0	0	0	0	0	0
-1.44	0	0.93	0.125	1.68	0	0.204	0	0	0
-1.41	0	0	0	0	0	0	0	0	0
-1.39	0	0.651	0	0.42	0.09	0.204	0	0	0
-1.15	0	0.837	0.25	0.42	0	0.204	0.192	0	0
-1.11	0	0.186	0	0	0	0	0	0.196	0
-1.11	0	0.465	0.25	0	0	0	0	0	0.091
-1.05	0	0	0	0	0	0	0	0	0
-1.04	0	0.279	0	1.26	0.09	0.068	0.096	0.098	0
-0.97	0	0.279	0	0	0	0	0	0	0
-0.96	0	0.186	0.25	0	0.09	0	0	0	0
-0.95	0	0	0.125	0.84	0	0	0	0	0
-0.92	0	0	0.125	0	0.36	0	0	0	0
-0.92	0	1.209	0.25	1.68	0	0.068	0	0	0
-0.91	0	0	0	0	0	0	0	0	0
-0.86	0	0.093	0.125	0	0	0	0	0	0
-0.82	0	0.186	0	1.26	0	0.136	0	0	0
-0.82	0	0.465	0.125	0.28	0	0	0	0	0
-0.81	0	0.372	0	1.68	0	0.068	0	0.049	0
-0.81	0	0	0	0	0	0	0	0	0
-0.8	0	0.093	0	0.98	0	0.068	0	0	0
-0.79	0	0	0	0	0	0	0	0	0

-0.78	0	0.558	0.625	0.14	0.72	0.136	0	0	0
-0.77	0	0.186	0.375	0.98	0.27	0.068	0	0	0
-0.76	0	0.186	0	0.14	0	0	0	0	0
-0.76	0	0	0	0	0	0	0	0	0
-0.74	0	0.279	0.25	1.96	0.45	0.476	0	0	0
-0.74	0	0	0	0	0	0	0	0	0
-0.73	0	0.279	0	1.12	0.09	0	0	0	0
-0.7	0	0.558	0.125	0.14	0.09	0	0	0	0
-0.68	0	0.372	0.375	1.26	0.45	0.34	0	0	0
-0.67	0	0.093	0.125	0.56	0.09	0	0	0	0
-0.67	0	0	0	0	0	0	0	0	0
-0.67	0	0.558	0.125	1.26	0	0.136	0.096	0	0
-0.66	0	0.372	0.125	0.42	0.18	0	0	0	0
-0.65	0	0	0	0	0	0	0	0	0
-0.65	0	0.186	0	0.28	0	0	0	0	0
-0.65	0	0.465	0	1.96	0.09	0.272	0.192	0	0
-0.65	0	0	0	0	0.36	0	0	0	0
-0.63	0	0	0	0	0	0	0	0	0
-0.61	0	0.093	0	0.28	0.18	0.272	0.192	0	0.091
-0.61	0	0	0	1.26	0.09	0.272	0	0	0
-0.6	0	0	0.125	0	0	0.068	0	0	0
-0.59	0	0	0	0.28	0.45	0.136	0	0	0
-0.59	0	0	0.125	0	0	0.068	0	0	0.091
-0.58	0	0.186	0	0	0.09	0.272	0	0	0
-0.58	0	0.186	0	0.42	0.18	0.204	0	0.049	0
-0.58	0	0	0	0	0	0	0	0	0
-0.57	0	0	0	0.28	0	0	0	0	0
-0.57	0	0.279	0	0.42	0	0	0	0	0
-0.56	0	0.465	0	0.28	0	0	0.48	0	0
-0.55	0	0	0	0	0	0.136	0	0	0
-0.55	0	0.279	0	0.42	0.36	0	0	0	0
-0.55	0	0.186	0	0.14	0.63	0	0	0	0
-0.55	0	0	0	0.28	0	0	0	0	0
-0.54	0	0	0	0	0	0	0	0	0
-0.54	0	0.279	0.125	0	0	0.204	0.096	0	0
-0.53	0	0	0	0	0.18	0	0	0	0
-0.52	0	0	0	0	0	0	0	0	0
-0.52	0	0	0	0	0	0	0	0	0
-0.52	0	0	0	0	0.09	0	0	0	0
-0.52	0	0	0	0	0.09	0	0	0	0
-0.51	0	0	0.125	0	0	0.068	0	0	0
-0.51	0	0	0	0	0	0	0	0	0
-0.5	0	0.279	0	0.84	0.45	0.68	0	0	0
-0.5	0	0	0.125	0	0	0.068	0	0	0
-0.5	0	0.186	0.125	1.12	0.81	0	0	0	0
-0.5	0	1.209	0.375	0.28	0.63	0	0	0	0
-0.5	0	0	0	0	0.09	0	0	0	0
-0.5	0	0.093	0	0	0	0	0	0	0
-0.5	0	0	0	0	0	0.068	0	0	0
-0.49	0	1.674	0.5	0.7	1.26	0.748	0	0	0

-0.49	0	0.558	0.25	1.82	0	0.816	0	0	0
-0.49	0	0	0	0	0	0	0	0	0
-0.48	0	0	0	0	0	0	0	0	0
-0.48	0	0.186	0	0.98	0.18	0.204	0	0	0
-0.48	0	0	0	0	0	0	0	0	0
-0.47	0	0	0	0	0	0	0	0	0
-0.47	0	0.651	0	0.56	0.63	0.204	0	0	0
-0.47	0	0.186	0.375	0.28	0.18	0.204	0	0	0.091
-0.47	0	0.372	0.25	0.84	0.45	0.068	0	0	0
-0.47	0	0	0	0	0	0	0	0	0.273
-0.47	0	0.093	0	1.54	0	0	0	0	0
-0.47	0	0	0	0	0	0	0	0	0
-0.47	0	0	0	0.7	0	0	0.096	0.049	0
-0.46	0	0.744	0.125	1.4	0	0	0	0	0
-0.46	0	0	0	0	0	0	0	0	0
-0.46	0	0	0	0	0	0.068	0	0	0
-0.45	0	0	0	0	0	0	0.096	0	0
-0.45	0	0	0	0	0	0	0	0	0
-0.45	0	0.186	0	0	0	0	0	0	0
-0.44	0	0.372	0	0.42	0	0	0	0	0
-0.44	0	0	0	0.14	0.36	0.272	0	0	0
-0.43	0	0	0	0	0	0	0	0	0
-0.43	0	0.093	0	1.12	0.09	0.136	0.192	0.049	0
-0.43	0	0	0	0	0.18	0.068	0	0	0
-0.43	0	0.186	0.125	0	0.09	0	0	0	0
-0.42	0	0	0	0	0	0	0	0	0
-0.42	0	0.279	0.125	0.28	0	0	0	0	0
-0.42	0	0.651	0.25	1.12	0.18	0.068	0	0	0
-2.54	0	0	0.25	0	0	0.068	0.384	0	0
-2.41	0	0	0	0	0	0	0.096	0	0
-2.1	0	0.186	0.25	0	0.27	0	0.096	0	0.091
-1.65	0	0.186	0.625	0.42	0.27	0.068	0	0	0
-1.56	0	0.093	0.25	0	0	0	0.288	0	0
-1.33	0	0	0	0.14	0.09	0	0	0	0
-1.23	0	0	0	0	0	0	0	0	0
-1.13	0	0.186	0.375	0	0	0	0.096	0	0
-1.12	0	0	0	0.14	0	0	0	0	0
